# Effects of preconception care and periconception interventions on maternal nutritional status and birth outcomes in low‐ and middle‐income countries: A systematic review

**DOI:** 10.1002/cl2.1156

**Published:** 2021-05-05

**Authors:** Zohra S. Lassi, Sophie G. E. Kedzior, Wajeeha Tariq, Yamna Jadoon, Jai K. Das, Zulfiqar A. Bhutta

**Affiliations:** ^1^ Robinson Research Institute University of Adelaide Adelaide Australia; ^2^ Faculty of Health and Medical Sciences, Robinson Research Institute University of Adelaide Adelaide Australia; ^3^ Aga Khan University Karachi Pakistan; ^4^ Department of Paediatrics Aga Khan University Hospital Karachi Pakistan; ^5^ Division of Women and Child Health Aga Khan University Hospital Karachi Pakistan; ^6^ Centre for Global Child Health The Hospital for Sick Children Toronto Canada

## Abstract

**Background:**

The preconception period is an ideal time to introduce interventions relating to nutrition and other lifestyle factors to ensure good pregnancy preparedness, and to promote health of mothers and babies. In adolescents, malnutrition and early pregnancy are the common challenges, particularly among those who live in low‐ and middle‐income countries (LMIC) where 99% of all maternal and newborn deaths occur. These girls receive little or no attention until their first pregnancy and often the interventions after pregnancy are too late to revert any detrimental health risks that may have occurred due to malnutrition and early pregnancy.

**Objectives:**

To synthesise the evidence of the effectiveness of preconception care interventions relating to delayed age at first pregnancy, optimising inter‐pregnancy intervals, periconception folic acid, and periconception iron‐folic acid supplementation on maternal, pregnancy, birth and child outcomes.

**Search Methods:**

Numerous electronic databases (e.g., CINAHL, ERIC) and databases of selected development agencies or research firms were systematically searched for all available years up to July 2019. In addition, we searched the reference lists of relevant articles and reviews, and asked experts in the area about ongoing and unpublished studies.

**Selection Criteria:**

Primary studies, including large‐scale programme evaluations that assessed the effectiveness of interventions using randomised controlled trials (RCTs) or quasi‐experimental designs (natural experiments, controlled before‐after studies, regression discontinuity designs, interrupted time series [ITS]), that targeted women of reproductive age (i.e., 10–49 years) during the pre‐ and periconceptional period in LMICs were included. Interventions were compared against no intervention, standard of care or placebo.

**Data Collection and Analysis:**

Two or more review authors independently reviewed searches, selected studies for inclusion or exclusion, extracted data and assessed risk of bias. We used random‐effects model to conduct meta‐analyses, given the diverse contexts, participants, and interventions, and separate meta‐analyses for the same outcome was performed with different study designs (ITS, RCTs and controlled before after studies). For each comparison, the findings were descriptively summarised in text which included detailing the contextual factors (e.g., setting) to assess their impact on the implementation and effectiveness of each intervention.

**Main Results:**

We included a total of 43 studies; two of these were included in both delaying pregnancy and optimising interpregnancy intervals resulting in 26 studies for delaying the age at first pregnancy (14 RCTs, 12 quasi‐experimental), four for optimising interpregnancy intervals (one RCT, three quasi‐experimental), five on periconceptional folic acid supplementation (two RCTs, three quasi‐experimental), and 10 on periconceptional iron‐folic acid supplementation (nine RCTs, one quasi‐experimental). Geographically, studies were predominantly conducted across Africa and Asia, with few studies from North and Central America and took place in a combination of settings including community, schools and clinical. The education on sexual health and contraception interventions to delay the age at first pregnancy may make little or no difference on risk of unintended pregnancy (risk ratio [RR], 0.42; 95% confidence internal [CI], 0.07–3.26; two studies, =490; random‐effect; *χ* ^2^ *p* .009; *I* ^2^ = 85%; low certainty of evidence using GRADE assessment), however, it significantly improved the use of condom (ever) (RR, 1.54; 95% CI, 1.08–2.20; six studies, *n* = 1604; random‐effect, heterogeneity: *χ* ^2^ *p* .004; *I* ^2^ = 71%). Education on sexual health and and provision of contraceptive along with involvement of male partneron optimising interpregnancy intervals probably makes little or no difference on the risk of unintended pregnancies when compared to education on sexual health only (RR, 0.32; 95% CI, 0.01–7.45; one study, *n* = 45; moderate certainty of evidence using GRADE assessments). However, education on sexual health and contraception intervention alone or with provision of contraceptive showed a significant improvement in the uptake of contraceptive method. We are uncertain whether periconceptional folic acid supplementation reduces the incidence of neural tube defects (NTDs) (RR, 0.53; 95% CI, 0.41–0.77; two studies, *n* = 248,056; random‐effect; heterogeneity: *χ* ^2^ *p* .36; *I* ^2^ = 0%; very low certainty of evidence using GRADE assessment). We are uncertain whether preconception iron‐folic acid supplementation reduces anaemia (RR, 0.66; 95% CI, 0.53–0.81; six studies; *n* = 3430, random‐effect; heterogeneity: *χ* ^2^ *p* < .001; *I* ^2^ = 88%; very low certainty of evidence using GRADE assessment) even when supplemented weekly (RR, 0.70; 95% CI, 0.55–0.88; six studies; *n* = 2661; random‐effect; heterogeneity: *χ* ^2^ *p* < .001; *I* ^2^ = 88%; very low certainty of evidence using GRADE assessments),and in school set‐ups (RR, 0.66; 95% CI, 0.51–0.86; four studies; *n* = 3005; random‐effect; heterogeneity: *χ* ^2^ *p* < .0001; *I* ^2^ = 87%; very low certainty of evidence using GRADE assessment). Data on adverse effects were reported on in five studies for iron‐folic acid, with the main complaint relating to gastrointestinal side effects. The quality of evidence across the interventions of interest was variable (ranging from very low to moderate) which may be attributed to the different study designs included in this review. Concerning risk of bias, the most common concerns were related to blinding of participants and personnel (performance bias) and whether there were similar baseline characteristic across intervention and comparison groups.

**Authors' Conclusions:**

There is evidence that education on sexual health and contraception interventions can improve contraceptive use and knowledge related to sexual health, this review also provides further support for the use of folic acid in pregnancy to reduce NTDs, and notes that weekly regimes of IFA are most effective in reducing anaemia. However the certainty of the evidence was very low and therefore more robust trials and research is required, including ensuring consistency for reporting unplanned pregnancies, and further studies to determine which intervention settings (school, community, clinic) are most effective. Although this review demonstrates promising findings, more robust evidence from RCTs are required from LMICs to further support the evidence.

## 1 PLAIN LANGUAGE SUMMARY

### 1.1 Do early interventions on sexual health for girls and young women in developing countries benefit mother and child health?

Education on sexual health and contraceptive interventions can improve contraceptive use and knowledge related to sexual health. Folic acid use before and during pregnancy can reduce neural tube defects (NTDs), and iron‐folic acid use before pregnancy can reduce anaemia.

#### 1.1.1 What is this review about?

The preconception period is an ideal time to introduce interventions relating to nutrition and other lifestyle factors to ensure good pregnancy preparedness, and to promote health of mothers and babies. In adolescents, malnutrition and early pregnancy are the common challenges, particularly among those who live in low‐ and middle‐income countries (LMICs) where 99% of all maternal and newborn deaths occur. These girls receive little or no attention until their first pregnancy, and often the interventions after pregnancy are too late to reverse any detrimental impacts on health.

This review aims to synthesise the evidence of the effectiveness of preconception care interventions relating to delayed age at first pregnancy, optimising inter‐pregnancy intervals, periconception folic acid and periconception iron‐folic acid supplementation on maternal, pregnancy, birth and child outcomes.

**What is the aim of this review?**

This Campbell systematic review summarises the evidence from 43 studies. Included studies examine the effect of interventions to delay, and increase the time between, pregnancies compared with no intervention or the standard care. The review also includes studies that compared folic‐acid or iron‐folic acid supplementation with placebo or no supplementation.

**What studies are included?**

Eligible studies had to be randomised control trials or quasi‐experimental trials to evaluate the impact of preconception intervention to delay the age at first pregnancy, optimise interpregnancy interventions and periconceptional folic acid and iron‐folic acid supplementation compared to control/placebo or standard care, among girls/women living in LMICs.

Forty‐three studies are included in the review. Of these, 26 were on delaying the age at first pregnancy, four on optimising interpregnancy intervals, five on periconceptional folic acid supplementation, and 10 on periconceptional iron‐folic acid supplementation.

**What are the findings of this review?**

Overall, interventions to delay the age at first pregnancy and optimising inter‐pregnancy intervals have a positive effect on the uptake and usage of contraceptives. Folic‐acid supplementation and iron‐folic acid supplementation have shown beneficial impacts on reducing neural tube defects and anaemia, respectively. In all cases, the evidence is of very low to moderate quality.

*Delay the age at first pregnancy*: Education on sexual health and contraception interventions to delay the age at first pregnancy may show improvements in the use of condoms. However, it did not show any improvement in reducing the risk of unintended pregnancy.

*Optimising inter‐pregnancy intervals*: Education on sexual health and provision of contraceptives, along with involvement of male partners on optimising interpregnancy intervals showed improvement in the uptake of contraceptive method. However, it makes little or no difference on the risk of unintended pregnancies when compared to education on sexual health only.

*Periconceptional folic‐acid supplementation* may reduce the incidence of neural tube defects.

*Periconceptional iron‐folic acid supplementation* may reduce anaemia when supplemented weekly and in school set‐ups. However, gastrointestinal side effects were commonly reported.

**What do the findings of this review mean?**

Our review highlights improvements in the uptake of contraceptives through education on sexual health interventions to delay the age at first pregnancy and increase the interval between pregnancies. Similarly, the review underscores a reduction in neglected tropical diseases and anaemia through periconceptional folic‐acid and iron‐folic acid supplementation.

However, the evidence was of very low to moderate quality and therefore further good quality research is recommended.

## 2 BACKGROUND

### 2.1 Description of the condition

Interest in preconception health for maximising gains for mothers and babies started with the release of the seminal report from Centre for Disease Control (Johnson et al., 2006). Further, in 2011, the World Health Organization (WHO) convened a meeting of experts where there was an overwhelming agreement on the potential for preconception care to have a positive impact on maternal and child health outcomes (WHO, 2013). Since then there is growing awareness of the importance of the preconception period and efforts have been made to increase awareness and promote reproductive health from adolescents onwards.

Preconception care, defined as a set of interventions that aim to identify and modify the biomedical, behavioural and social risks to the woman's health or pregnancy outcome through prevention and management (WHO, 2013), is important for healthy maternal, birth and neonatal health outcomes (Dean et al., 2013). Optimising a woman's health before planning and conceiving pregnancy is increasingly recognised as an important strategy to enhance maternal and child health (Dean, 2013b). The preconception and periconception (i.e., before and during early pregnancy) period is an ideal time to introduce interventions relating to nutrition and other lifestyle factors to promote health and for ensuring good pregnancy preparedness. Since 99% of all maternal and newborn deaths occur in LMICs (WHO, 2017), early start of preconception care particularly for girls living in LMICs is very crucial. At present, policies and guidelines on preconception care are scarce, and care starts when the woman becomes pregnant which then extends to childbirth and postnatal period (for mothers and babies). There is a clear gap in the continuum of care, particularly for young girls who enter the reproductive years and women who are not pregnant. These girls and women receive little to no attention until their first pregnancy. Evidence also suggests that antenatal care is often too late to revert the detrimental health risks and issues that may have impacted the developing foetus (Dean et al., 2013).

Adolescents face multiple challenges to their health and social well being if they become pregnant early in life. Approximately 13% of all maternal mortality occurs in adolescents (WHO, 2014). The risk of maternal mortality is approximately five times higher for adolescents under the age of 15 years and twice as high for adolescents between 15 and 19 years of age compared to women aged 20–29 years (Nove et al. 2014). These girls are at a higher risk for developing hypertension during pregnancy, severe anaemia, bleeding and infection. Because their pelvises have not developed enough for the baby to pass through the birth canal, adolescent girls have a higher risk of obstructed labour, stillbirths; and their newborns are also more likely to be born prematurely, have low birth weight, or die in the 1st month of life (Gibbs et al., 2012; Paranjothy, 2009; WHO, 2007). These risks are further exacerbated by factors such as poverty, illiteracy, limited access to health care, lack of social support from family, and absence of autonomy for decision making (Nove et al., 2014). Apart from direct health consequences to the mother and baby, early motherhood is often linked to school drop‐out, social difficulties and poor socioeconomic status (Penman‐Aguilar, 2013). They also have higher odds of experiencing depressive symptoms including loneliness, sleep disorders, loss of appetite and even thoughts of harming oneself or the baby within the 3 months after birth (Reid & Meadows‐Oliver, 2007). The evidence further details that children born to teenage mothers tend to have poorer health, poor cognitive development, behavioural problems and poor educational outcomes; they also have a high probability of becoming a teen parent themselves (Black et al., 2002). Therefore it is important to encourage the use of contraceptives and educate the importance of planning pregnancy and delaying first pregnancy until the woman is at least 18 years of age which allows a woman's body to fully mature.

Maternal nutritional deficiencies particularly iron and folate are common in LMICs. Anaemia in women from LMICs is due to low dietary intake of bioavailable iron combined with endemic infectious diseases such as helminthiasis, which puts women at increased risk during pregnancy. Low preconception haemoglobin and ferritin levels increase the risk of poor foetal growth and low birth weight (Dean et al., 2014a). Similarly, folate deficiency can lead to the development of NTDs in the foetus. Other micronutrients such as zinc, vitamin B and calcium have been found to improve maternal and newborn outcomes when supplementation is provided during pregnancy; however, their impact during the preconception period has not been established (Ramakrishnan, 2012). Improved reproductive health and planning is the fundamental component of preconception care and starting early interventions, such as providing essential nutritional supplements in the preconception period, can help women begin pregnancy in their best health.

### 2.2 Description of the intervention

Each year an estimated 140 million births take place (WHO, 2018a). Of these 16 million occur to adolescents between the ages of 15–19 years and approximately 2.5 million to girls <16 years of age (WHO, 2018b). It is therefore important to delay the age at first pregnancy and optimise the interpregnancy intervals as well as providing preconception supplements of the essential micronutrients to promote a healthy pregnancy.

Many adverse maternal, neonatal and pregnancy outcomes may be avoidable if the age at first pregnancy is optimal or that appropriate intervals between pregnancies are achieved. Previous evidence has shown the benefits of delayed childbearing, specifically in adolescence, as adolescent pregnancy is known to be associated with an increased risk of preterm birth, stillbirth, small‐for‐gestational age, neonatal mortality and complications during labour and delivery (Haldre et al., 2007; Paranjothy, 2009; WHO, 2007). However, there is variable evidence related to prolonging inter‐pregnancy intervals. In a systematic review, Conde‐Agudelo et al. (2012) identified that compared with inter‐pregnancy intervals of 18–23 months, inter‐pregnancy intervals shorter than 6 months were associated with increased risks of preterm birth, low birth weight, and small‐for‐gestational age babies. While delaying the age of first pregnancy ensures the maturation and growth of the mother's body, optimising pregnancy intervals gives time for body to recover and prepare itself for another pregnancy. This review considered interventions to delay the age of first pregnancy or to optimise birth intervals. Interventions can include education on sexual health, contraception education and distribution, individual counselling or sex education and can be either population‐based, community based, school based, hospital/clinic based as well as target specific groups such as teenagers and be delivered by health professionals or workers.

On the other hand, the benefits of micronutrient supplementation during pregnancy are well‐established, particularly for iron and folic acid. There are numerous nutrition related interventions targeting different vitamins and nutrients to improve maternal and neonatal outcomes. While folic acid may be one of the most widely known, there is evidence that multivitamins and other nutrients have a critical role in brain and nervous system development as well as impact the immune system during pregnancy, specifically relating to the inflammatory response (Ramakrishnan, 2012). Specifically, interventions have shown that vitamin A received during pregnancy may reduce maternal anaemia in women who likely have a vitamin A deficiency, however this review also demonstrated that vitamin A did not reduce maternal or newborn mortality (McCauley et al., 2015). Another review investigating the use of supplementing pregnant women with vitamin D, demonstrated that vitamin D may reduce the risk of pre‐eclampsia, low birthweight and preterm birth (De‐Regil et al., 2016). There is also evidence for the use of multivitamin supplementation with iron and folic acid to reduce the risk of miscarriage (Balogun et al. 2016).

However, there is limited evidence for micronutrient supplementation specifically during pre‐ and periconception apart from the use of folic acid, which has shown to reduce NTDs (De‐Regil et al., 2015). The intermittent utilisation of iron and folic acid prior to conception has shown to reduce the risk of anaemia in reproductive age women, though additional evidence is needed to support improvements in other maternal and newborn outcomes (Fernández‐Gaxiola & De‐Regil, 2011). Ideally many of these interventions would have a preventative focus, for example, iron and folic acid supplementation, food fortification or dietary diversification to decrease the incidence of anaemia in women before they become pregnant.

### 2.3 How the intervention might work

Pregnancy in the teenage years is associated with multiple risks and delaying the age of first pregnancy can reduce these risks. Interventions such as sex education and counselling at school and community settings by peers and community health workers have shown impact (Brieger 2001; García et al., 2012). Such interventions improve knowledge and promote attitudinal and behaviour change among young adolescents. These interventions also promote use of condoms and other birth control mechanism including abstinence (Cabezón et al., 2005). Interventions to delay pregnancy can include health education, contraception education and distribution, skills building and different forms of counselling (Oringanje et al. 2009). One intervention commonly utilised in LMICs is cash transfer programmes to encourage adolescent women to stay in school for longer and as a consequence avoid early marriage or sexual initiation (Baird et al., 2010). A systematic review by Khan et al. (2016) assessed the evidence for using conditional and unconditional cash transfers as a method to encourage contraceptive use in LMICs. The majority of the included studies utilised cash transfers to encourage school attendance or aimed to improve overall health and nutrition. While there were few available studies specifically targeting contraception, some studies did demonstrate a positive impact on contraceptive use and a decrease in fertility outcomes (i.e., number of pregnancies resulting in live births).

Similarly, optimising the birth interval has shown positive impacts for mothers and babies (Afeworki et al., 2015). Studies have shown that inter‐pregnancy intervals of <12 or >60 months have an adverse effect on perinatal outcomes such as preterm birth, low birth weight, small for gestational age babies and congenital defects in babies (Dean et al., 2014b). While short pregnancy intervals (<12 months) are associated with anaemia, puerperal endometritis, and premature rupture of membrane, longer intervals (>60 months) are associated with preeclampsia, third trimester bleeding and foetal death (Dean et al., 2014b). Furthermore the risks for folate and other nutritional deficiencies, cervical insufficiency, suboptimal breastfeeding, incomplete healing of uterine scar from previous caesarean delivery, and abnormal remodelling of endometrial blood vessels are higher for closely‐spaced pregnancies (Conde‐Agudelo et al., 2012). Therefore, it is important to intervene to delay the age of first pregnancy and optimise the intervals between the two pregnancies. There are numerous approaches that an intervention to promote birth spacing for women of reproductive age may undertake. Strategies may involve policies or population‐based interventions, or a combination of school and community‐based approaches (Aslam et al., 2015). Much like interventions focusing on delaying pregnancy, strategies that encourage women and couples to employ suitable spacing between births may involve health education, skills building and contraception education and distribution to ensure appropriate and consistent use of contraceptives (Aslam et al., 2015; Dean et al., 2014b). As per Aslam et al. (2015), these interventions may work by encouraging mothers to pursue educational avenues or work related accomplishments, in order to develop self‐confidence, self‐esteem and autonomy. A recent review on birth spacing interventions in low‐, middle‐ and high‐income countries found studies with high quality evidence for a positive impact for spacing on repeat pregnancy/birth (Norton et al., 2017). Successful interventions included those that targeted adolescents to teach them planning skills, including activities that involve preparing contraceptive plans.

Folate plays an important role in protein synthesis and metabolism and other processes related to cell multiplication and tissue growth. Its deficiency during pregnancy causes megaloblastic anaemia and accumulates homocysteine in the serum which is associated with an increased risk in cardiovascular disease, late pregnancy complications such as pre‐eclampsia, and NTDs around the time of conception (Lassi & Bhutta, 2012). The literature shows that iron supplementation during pregnancy can be a protective factor against low birth weight, and given alone or with folic acid it is effective in increasing iron stores and preventing anaemia during later gestation (Fernández‐Gaxiola & De‐Regil, 2011). Since women might not know when they become pregnant, it is important to ensure iron and folic acid sufficiency from early in life. Health promotion campaigns are a common strategy to increase a population's knowledge and awareness on the benefits and importance of nutritional supplementation. Two systematic reviews (Chivu et al., 2008; Rofail et al., 2012) found that while these campaigns were successful in increasing overall awareness, knowledge and consumption of folic acid before and during pregnancy, the increase in folic acid consumption was nowhere near optimal despite women having significantly increased knowledge. Interventions specific to adolescent nutrition are often conducted in school‐based settings, one meta‐analysis conducted by Salam et al. (2016) demonstrated that school‐based delivery significantly reduced anaemia.

### 2.4 Why it is important to do this review

Preconception, the time prior to pregnancy, and periconception, the time before a woman conceives which continues early pregnancy, are two significant periods which have been shown to impact a range of maternal and neonatal health outcomes. While there is increasing evidence to support the provision of preconception care, the effectiveness of interventions to delay the age of first pregnancy, optimise birth intervals, and to provide periconception iron and folic acid supplementation require further investigation. Determining the most effective delivery mechanisms across different settings is vital to successfully implementing pre‐ and periconception interventions in LMIC settings (Poels, 2016). While there are existing reviews examining the effects of various interventions on preventing teen pregnancies (Dean et al., 2014b; Oringanje et al., 2009), they have mainly included randomised controlled trials (RCTs). In this review, we also included evidence from large‐scale quasi‐studies in addition to relevant trials, as randomisation is not always possible for all settings and populations. We wanted to ensure that our review is comprehensive and includes nonrandomised studies as contextual and supplementary evidence for the included RCTs (Schünemann et al., 2013). These studies can benefit systematic reviews by demonstrating whether an intervention works in different populations, whether there are possible interaction effects and can describe long‐term outcomes.

We did not identify any review of interventions to optimise pregnancy intervals. Existing reviews have only examined pregnancy intervals from observational studies (Brown et al., 2013; Conde‐Agudelo et al., 2012; Dean et al., 2014b), therefore it is important to review the evidence of interventions to prolong inter‐pregnancy intervals and their impact on maternal nutrition and birth outcomes.

Existing reviews have also evaluated the effects of periconception folic acid use (De‐Regil et al., 2015; Dean et al., 2014a; Ramakrishnan, 2012) and iron folic acid use (Fernández‐Gaxiola & De‐Regil, 2011) but some of these reviews are outdated and focus exclusively on RCTs, therefore there is a need to update the evidence from RCTs and other large scale quasi study.

This review aimed to synthesise the evidence on the effectiveness of preconception care interventions relating to delayed age at first pregnancy, optimising inter‐pregnancy intervals, periconception folic acid, and periconception iron‐folic acid supplementation on maternal and neonatal outcomes by systematically reviewing the primary studies, along with rigorous evaluations of existing programmes. This approach enabled a comprehensive assessment of the effectiveness of these interventions for improving maternal, neonatal and related outcomes (e.g., nutritional). For example, the primary maternal outcomes for this review included (unintended) pregnancy, anaemia and iron deficiency anaemia, while the primary neonatal outcomes included NTDs, stillbirth, perinatal/neonatal mortality and low birth weight. Secondary outcomes for maternal and neonatal health were also investigated and are listed in the methods section.

## 3 OBJECTIVES

The overall objective was to assess the effectiveness of the following pre‐ and periconception interventions when compared with no intervention or standard of care in LMICs.

1. Interventions to delay age at first pregnancy
2. Interventions to optimise interpregnancy intervals
3. Periconception folic acid supplementation
4. Periconception iron‐folic acid supplementation.

## 4 METHODS

### 4.1 Criteria for considering studies for this review

#### 4.1.1 Types of studies

The review is based on a published protocol (Lassi et al., 2019). We included primary studies, including large‐scale programme evaluations, to assess the efficacy and/or effectiveness of interventions using the following study designs:

- RCTs, where participants were randomly assigned, individually or in clusters, to intervention and comparison groups. Cross‐over designs were eligible for inclusion.
- Quasi‐experimental designs, which include:
  a. Natural experiments: studies where nonrandom assignment is determined by factors that are out of the control of the investigator. One common type includes allocation based on exogenous geographical variation.
  b. Controlled before‐after studies (CBA), in which measures were taken of an experimental group and a comparable control group both before and after the intervention. We also required that appropriate methods were used to control for confounding, such as statistical matching (e.g., propensity score matching, or covariate matching) or regression adjustment (e.g., difference‐in‐differences, instrumental variables).
  c. Regression discontinuity designs; allocation to intervention/control is based upon a cut‐off score.
  d. Interrupted time series (ITS) studies, in which outcomes were measured in the intervention group at least three time points before the intervention and after the intervention.

Pre‐post studies *without* a control group were not included.

It was noted whether any studies were ongoing or awaiting classification and language was not included as a restriction for inclusion.

#### 4.1.2 Types of participants

The target population were women of reproductive age (i.e., 10–49 years) including adolescent girls, regardless of health status, living in LMICs. Country income was classified according to the 2018 World Bank list of country economies (World Bank, 2018). Interventions were aimed at nonpregnant women, and the outcomes were measured before and during pregnancy and on their children. For optimising birth intervals we considered interventions given while they were pregnant to optimise birth intervals for the next pregnancy.

We compared the World Bank list of country economies over years and consider all those studies conducted in countries which were part of LMICs before 2018.

#### 4.1.3 Types of interventions

The following interventions targeting women of reproductive age (10–49 years) including adolescent girls (10–19 years) during the pre‐ and periconception period in LMICs were included:

- Interventions to delay age at first pregnancy such as curriculum based sex education, abstinence alone programmes, interactive computer based interventions, and so forth.

a. Education on sexual health and contraceptive promotion provided at the community, school or household level by parents, colleagues, teachers, health workers or social workers to adolescents and young women

- Interventions to optimise interpregnancy intervals such as introducing family planning methods, abstinence alone programmes and so forth.

a. Education on sexual health interventions and contraceptive promotion provided at the community, school or household level by parents, colleagues, teachers, health workers or social workers to mothers of reproductive age.

- Periconception folic acid

a. Pubescent or menstruating women who received any folic acid supplementation before conception and continued using until the first trimester of pregnancy.

- Periconception iron folic acid

a. Pubescent or menstruating women who received any iron folic acid supplementation before conception and continued using until the first trimester of pregnancy.

These interventions were compared against no intervention, standard of care (whatever is applicable in the setting the study was conducted), or placebo. Folic acid and iron‐folic acid use only during pregnancy was not included. We excluded multiple micronutrient powders for point‐of‐use fortification of foods, fortification of staple foods, water, condiments or seasonings with folic acid or iron and other micronutrients or the provision of oral contraceptives that contain folic acid in this review. We excluded fortification programmes because they are employed universally and no exact period of start and end of intake was known to generate evidence for recommendation. We also excluded oral contraceptives that contain folic acid because they merit a separate review.

#### 4.1.4 Types of outcome measures

##### Primary outcomes

Maternal

- Unintended pregnancy
- Anaemia
- Iron deficiency anaemia.

Neonatal

- Neural tube defects
- Stillbirth
- Perinatal mortality
- Neonatal mortality
- Low birth weight.

##### Secondary outcomes

Maternal

- Reported changes in knowledge and attitudes about the risk of unintended pregnancies
- Initiation of sexual intercourse
- Use of birth control methods
- Serum folate
- Adverse effects
- Adherence to folic acid or iron folic acid supplementation
- Abortion
- Miscarriage
- Maternal mortality.

Neonatal

- Preterm birth
- Small‐for‐gestational age
- Other congenital anomalies
- Admission to special care for any cause.

###### Duration of follow‐up

For interventions to delay the age at first pregnancy: we considered studies that provided intervention at anytime during preconception period.

For interventions to optimise interpregnancy intervals: we considered studies that have provided interventions to optimise inter‐pregnancy intervals at any time during the previous pregnancy, as well as included interventions implemented after giving birth to the last child.

For folic acid and iron‐folic acid supplementation: we considered studies if folic acid and iron‐folic acid were supplemented during both the pre‐ and periconception period.

###### Types of settings

Any setting in LMICs.

### 4.2 Search methods for identification of studies

We did not impose any restrictions, for example, language or publication status or the publication dates, on the literature searches described below.

#### 4.2.1 Electronic searches

The search was performed in the following electronic databases: CABI's Global Health, CINAHL,Cochrane Controlled Trials Register (CENTRAL), Dissertation Abstracts International, EMBASE, Epistemonikos, ERIC, HMIC (Health Management Information Consortium), MEDLINE, Popline, PsycINFO, Scopus, Social Science Index from Web of Science, Sociofiles, WHO's Global Health Library, WHO Reproductive Health Library, and the WHO nutrition databases (http://www.who.int/nutrition/databases/en/). We also searched the web sites of selected development agencies or research firms (e.g., Journal of Librarianship and Information Science (https://journals.sagepub.com/home/lis), International Food Policy Research Institute (http://www.ifpri.org/), National Bureau of Economic Research (https://www.nber.org/), United States Agency for International Development (https://www.usaid.gov/), World Bank (https://www.worldbank.org/)) and Google Scholar (https://scholar.google.com/). The last search was performed on July 31, 2019, and there was no restriction placed on publication date.

**Figure 1:**
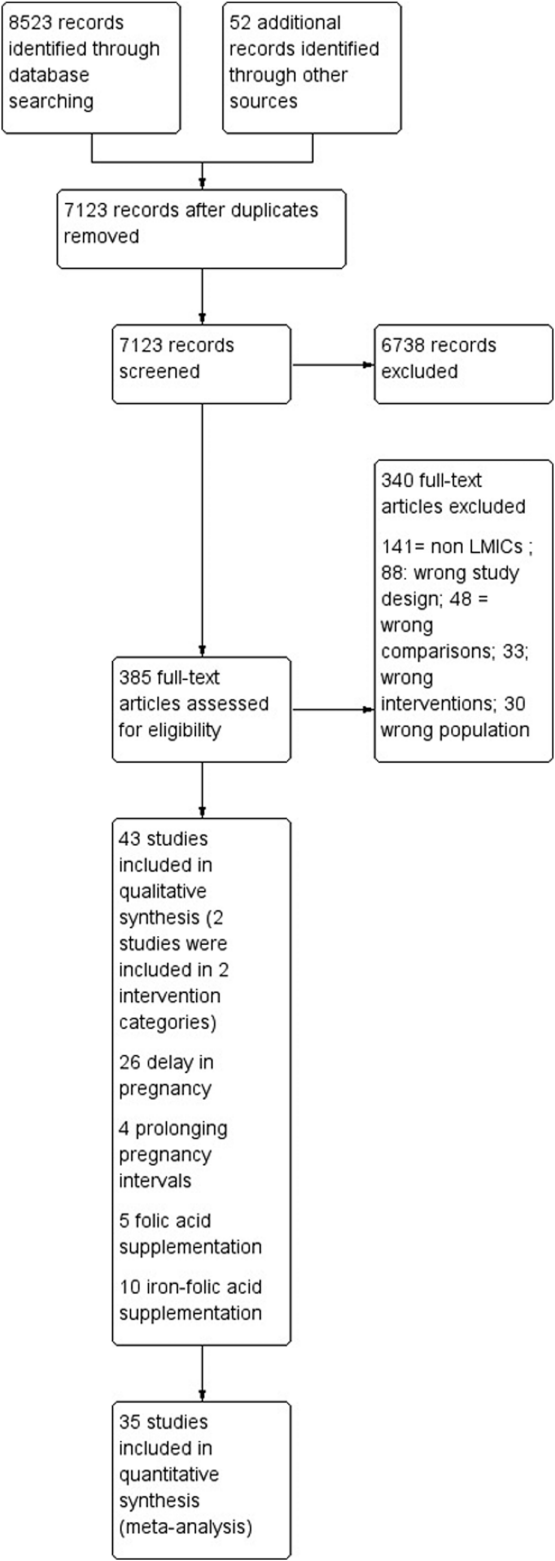
Study flow diagram

The search strategy can be found in Appendix A.

#### 4.2.2 Searching other resources

We checked the reference lists of all included studies and systematic reviews for additional references. We attempted to make every effort to contact relevant organisations and experts in the field to identify unpublished or ongoing studies. References of included articles, relevant reviews, and annotated bibliographies were scanned for eligible studies.

### 4.3 Data collection and analysis

We conducted data collection and analysis in accordance with the Cochrane Handbook for Systematic Reviews of Interventions (Higgins et al. 2011).

#### 4.3.1 Description of methods used in primary research

For this review, the primary research designs of interest were experimental and quasi‐experimental study designs as well as nonrandomised studies with a control group, including CBA. We also accepted ITS studies with at least three time points before and three time points after the intervention. The studies included women in the preconception period for the intervention to delay age at first pregnancy or optimise pregnancy intervals. Studies also included women in the periconceptional period for the supplementation of iron and iron‐folic acid.

#### 4.3.2 Criteria for determination of independent findings

Before initiating the synthesis (detailed below), we ensured that all articles reporting on the same study were appropriately linked. To ensure independence and appropriate combination of outcome constructs, syntheses were conducted according to the type of interventions specified above. If multiarm studies were included, intervention groups were combined or separated into different forest plots, and we ensured that there was no double counting of participants. If an outcome is reported in several different metrics, we performed unit conversions in order to pool the data. We anticipated differences in the types of literature and therefore ensured that any analysis took possible sources of dependency into account by grouping papers into studies and ensuring that no double counting of evidence took place when synthesising across studies.

#### 4.3.3 Details of study coding categories

Two review authors in pairs (S. K., W. T., and Y. J.) extracted data independently and a third review author (Z. S. L.) checked for reliability and resolved any conflict. We extracted the primary data for the study characteristics including details of the populations, setting, sociodemographic characteristics, interventions, comparators, outcomes and study design in duplicate. We checked primary study data for accuracy. Disagreements were resolved by discussion or consultation with a third reviewer.

The following information was extracted for each included study:

- Background: time period when study took place, type of publication (e.g., full‐text journal article, abstract, conference paper, thesis), study country or countries, funding source(s), and conflicts of interest
- Population and setting: population age and setting
- Methods: Study design, description of study arms, unit of allocation, sample or cluster size per study arm (for individually or cluster randomised trials respectively), start and end date, follow‐up
- Participants: total number randomised/allocated, sociodemographic data
- Intervention group details: number randomised/allocated to group, description of intervention, duration and follow‐up, timing, delivery of intervention, providers and their training. We described all the study intervention arms in the tables of included studies, however, we only reported the intervention arms that met the review inclusion criteria.
- Comparison group details: number randomised to group, description of comparison, duration and follow‐up, timing, providers and their training
- Outcomes: measurement tool, validation of the tool, total number in intervention and comparison groups, change indicated at each time point
- Other information

#### 4.3.4 Selection of studies

Two review authors (Z. S. L. and S. K.) independently screened titles and abstracts of all retrieved references. We retrieved the full‐text study reports for all citations that at least one review author considered potentially relevant. Two review authors (Z. S. L. and S. K.) independently screened the full text articles and identified studies for inclusion, as well as recorded reasons for exclusion of ineligible studies in a “Characteristics of excluded studies” table. We resolved any disagreement through discussion or, if required, we consulted a third review author. We identified and excluded duplicates and collated multiple reports of the same study so that each study, rather than each report, is the unit of interest in the review. We recorded the selection process in sufficient detail to complete a Preferred Reporting Items for Systematic Reviews and Meta‐Analyses (PRISMA) flow diagram (Moher et al., 2009) (Figure 1).

#### 4.3.5 Data extraction and management

Two review authors in pairs (S. K., W. T., Y. J.) independently extracted data on data extraction sheet. We resolved any disagreement through discussion or, if required, we consulted a third review author (Z. S. L.). We used a piloted data collection form for study characteristics and outcome data. If any information from the study was unclear or missing, we contacted the authors of the original papers for further details. Data were recorded on a data extraction form that summarised key characteristics of the review/studies including

- Methods: study design, study duration;
- Details of study participants (including their age, socioeconomic status, parity): numbers randomised, inclusion and exclusion criteria
- Interventions: content of intervention, duration of intervention, timing of intervention, comparisons
- Outcomes and time point

#### 4.3.6 Assessment of risk of bias in included studies

Two review authors (W. T. and Y. J.) independently assessed the risk of bias for each included study. We resolved any disagreements by discussion or by involving a third review author (Z. S. L.).

For RCTs including cluster RCTs, we used the Cochrane Collaboration Risk of Bias tool (Higgins et al., 2011). We assessed the risk of bias according to the following domains. Each criterion was rated as high, low and unclear risk.

- Random sequence generation
- Allocation concealment
- Blinding of participants and personnel
- Blinding of outcome assessment for each outcome
- Incomplete outcome data
- Selective outcome reporting
- Other bias such as validity of outcome measure and baseline comparability.

For CBA and ITS, we used EPOC methods (EPOC, 2017). Each criterion was rated as high, low or unclear risk.

- Random sequence generation
- Allocation concealment
- Baseline outcome measurements
- Baseline characteristics
- Incomplete outcome
- Knowledge of the allocated interventions adequately prevented during the study
- Protection against contamination
- Selective outcome reporting
- Other risks of bias.

We constructed “Summary of findings” tables for all of the primary outcomes using the Grading of Recommendations Assessment, Development and Evaluation (GRADE) criteria (Guyatt et al., 2011) which takes into consideration risk of bias and other criteria. This enabled us to determine the quality of evidence for each outcome and ensures whether these findings should be considered with caution.

#### 4.3.7 Measures of treatment effect

We uploaded the outcome data for each study into the data tables in RevMan to calculate the treatment effects (RevMan, 2014). We used risk ratio (RR) for dichotomous outcomes. We used the mean difference (MD) for continuous outcomes reported on the same scale, and the standardised mean difference (SMD) for continuous outcomes reporting the same outcome but measured on different scales in different studies included in the same meta‐analysis. We expressed uncertainty with 95% confidence intervals (CIs) for all effect estimates. When means and *SD*s were not reported, we used other available data (e.g., confidence intervals, *t* statistic values, *p* values) to calculate the appropriate effect sizes as described in the Cochrane Handbook for Systematic Reviews of Interventions (Higgins & Green, 2011) Where other available data were not sufficient to calculate *SD*s, we contacted the study authors. When we were unable to enter the results in either way, we described them in the “Characteristics of included studies” tables or entered the data into the “Additional tables” section. We also considered the possibility and implications of skewed data when analysing continuous outcomes as they can provide misleading results due to small sample size. We also examined any relevant retraction statements and errata for information. Outcomes from studies with multiple groups arms were analysed in an appropriate way to avoid double counting of participants by adding them to different subgroups within the same plot. In such scenario, we did not report the overall pooled estimate and we only reported subgroup pooled estimate.

#### 4.3.8 Unit of analysis issues

We performed a separate meta‐analysis for the same outcome with different study designs (ITS, RCT, and CBA). We also performed a separate meta‐analyses for each topic mentioned as a separate objective. We assessed the effectiveness of each intervention as a subgroup. Where studies have used clustered randomisation, we anticipated that the study investigators presented their results after appropriately controlling for clustering effects (e.g., variance inflated standard errors, hierarchical linear models). If it was unclear whether a cluster‐RCT has appropriately accounted for clustering, the study investigators were contacted for further information. Where appropriate controls for clustering were not used, we requested an estimate of the intra‐class correlation coefficient (ICC). In the unlikely event that authors did not respond to our request for ICC estimates, we took ICC reported in similar studies from similar context. Following this, effect sizes and standard errors were meta‐analysed in RevMan using the generic inverse method (Higgins & Green, 2011). They were combined with estimates from individual level studies.

#### 4.3.9 Dealing with missing data

We contacted study authors to verify key study characteristics and obtained missing numerical outcome data where possible (e.g., when we identified a study as an abstract only). If numerical outcome data were missing, such as *SD*s or correlation coefficients (for cluster designs to adjust for clustering effect), we calculated them from other available statistics, such as *p* values, according to the methods described in the Cochrane Handbook for Systematic Reviews of Interventions (Higgins et al., 2011).

#### 4.3.10 Assessment of heterogeneity

Statistical heterogeneity were assessed using *τ* ^2^, *I* ^2^ and significance of the *χ* ^2^ test; we also assessed heterogeneity visually using forest plots. Based on prior theory and clinical knowledge, we expected clinical and methodological heterogeneity in effect sizes in this literature. Therefore, we attempted to explain any observed statistical heterogeneity using subgroup analysis.

#### 4.3.11 Assessment of reporting biases

If sufficient studies were found, funnel plots were drawn to investigate any relationship between effect size and study precision. Ten studies are usually considered sufficient to draw a funnel plot. As a direct test for publication bias, we compared results extracted from published journal reports with results obtained from other sources (including correspondence). While funnel plot asymmetry may indicate publication bias, this is not inevitably the case, and possible explanations for any asymmetry found was considered and discussed in the text of the review.

#### 4.3.12 Data synthesis

We carried out statistical analysis using RevMan (2014) software.

We prepared a matrix of all studies for each intervention which outlines all the differences in the studies at the intervention, duration, timing, and so forth, and examined how to pool them together. We used random effects meta‐analyses, given the diverse contexts, participants and interventions.

For each comparison, we descriptively summarised the findings from the contextual factors such as setting, timings of intervention, duration of intervention, people delivering interventions, to assess their impact on the implementation and effectiveness of each intervention.

#### 4.3.13 “Summary of findings” tables

We constructed “Summary of findings” tables for all of the primary outcomes using the Grading of Recommendations Assessment, Development and Evaluation (GRADE) criteria (Guyatt et al., 2011). Two review authors (W. T., Y. J.) undertook GRADE assessments independently and compared results. If there was a disagreement on GRADE assessments, consensus was reached through discussion (Z. S. L.). We justified all decisions to downgrade the certainty of the evidence using footnotes and we made comments to aid readers' understanding of the review where necessary. We considered the following factors when we assessed the certainty of evidence.

- Limitations in the study design and conduct (i.e., risk of bias)
- Inconsistency of results
- Indirectness of evidence
- Imprecision
- Publication bias.

We downgraded the certainty of the evidence for a specific outcome by one level according to the performance of the included studies against each of the five factors.

#### 4.3.14 Subgroup analysis and investigation of heterogeneity

When there was sufficient number of studies included in the outcomes, we conducted the subgroup analyses on the following domains.

- Setting (home, facility based, community level, school, work)
- Timing of intervention (preconception, periconception, prenatal, postpartum)
- Type of intervention (school based education, abstinence only programme, contraceptive promotion, and so forth).

The subgroup analyses was conducted using Review Manager 5.3 with a test for interaction.

#### 4.3.15 Sensitivity analysis

We planned to undertake sensitivity analyses to assess the potential biasing effects of using the ICC that have been derived in different ways. We were unable to perform these analyses due to insufficient number of studies per outcome.

#### 4.3.16 Treatment of qualitative research

Qualitative research was outside the scope of this review.

## 5 RESULTS

### 5.1 Description of studies

Refer to Characteristics of included studies; Characteristics of excluded studies; Characteristics of studies awaiting classification; Characteristics of ongoing studies. No studies were identified for the categories of awaiting classification or ongoing.

#### 5.1.1 Results of the search

We identified a total of 8523 papers from the different search engines, all of the included data is from published literature. After removing duplicates, 7123 abstracts were reviewed. Of those 323 full texts were reviewed and finally 43 studies were included (Figure 1), two studies were included for both delaying pregnancy in optimising interpregnancy intervals. Of these, 26 were on delay in the age of pregnancy, four on optimising interpregnancy birth intervals, five on folic acid supplementation, and 10 on iron‐folic acid supplementation.

#### 5.1.2 Included studies

##### Delaying pregnancy

###### Included studies

We included a total of 26 studies for interventions for delaying pregnancy (Baird et al., 2012; Cabezón et al., 2005; Cowan et al., 2010; Daniel et al., 2008; Diop et al., 2004; Duflo et al., 2015; Dupas, 2011; Erulkar & Muthengi, 2007; Gallegos et al., 2008; Handa et al., 2015; James et al., 2005; Jewkes et al., 2006; Kaljee et al., 2005; Kanesathasan et al., 2008; Klepp et al., 1997; Lou et al., 2004; Martiniuk et al., 2003; Meekers, 2000; Mmbaga et al., 2017; Okonofua et al., 2003; Pandey et al., 2016; Ross et al., 2007; Shuey et al., 1999; Speizer et al., 2001; Walker et al., 2006; Ybarra et al., 2013); 14 were RCTs and 12 were quasi‐experimental (9 natural experiments and 3 were CBA). All of the studies focused on maternal outcomes, including unintended pregnancy, reported changes in knowledge and attitudes about the risk of unintended pregnancies, initiation of sexual intercourse, use of birth control methods, and abortion.

###### Outcomes

The primary outcome that has been reported is unintended pregnancy and the secondary outcomes that were reported are changes in knowledge and attitudes about the risk of unintended pregnancies, initiation of sexual intercourse and use of birth control methods, and abortion.

###### Settings

Nineteen studies were conducted in Africa; one in Cameroon (Speizer et al., 2001), one in Ethiopia (Erulkar & Muthengi, 2007), three in Kenya (Duflo et al., 2015; Dupas, 2011; Handa et al., 2015), one in Malawi (Baird et al., 2010), one in Nigeria (Okonofua et al., 2003), one in Senegal (Diop et al., 2004), three in South Africa (James et al., 2005; Jewkes et al., 2006; Meekers, 2000), three in Tanzania (Klepp et al., 1997; Mmbaga et al., 2017; Ross et al., 2007), two in Uganda (Shuey et al., 1999; Ybarra et al., 2013), and one in Zimbabwe (Cowan et al., 2010). Five studies took place in Asia; one in China (Lou et al., 2004), three in India (Daniel et al., 2008; Kanesathasan et al., 2008; Pandey et al., 2016), one in Vietnam (Kaljee et al., 2005). Three in North America: Mexico (Gallegos et al., 2008; Walker et al., 2006) and one in Belize (Martiniuk et al., 2003). One in South AMerica: Chile (Cabezón et al., 2005).

The interventions for the studies in this review took place in varying combinations of communities, schools and clinics. Most of the studies were conducted in schools only (Cabezón et al., 2005; Duflo et al., 2015; Dupas, 2011; Erulkar & Muthengi, 2007; Gallegos et al., 2008; James et al., 2005; Klepp et al., 1997; Martiniuk et al., 2003; Okonofua et al., 2003; Shuey et al., 1999; Walker et al., 2006; Ybarra et al., 2013). One study was conducted in both community and schools (Baird et al., 2010), while there were seven studies had only community based interventions (Daniel et al., 2008; Handa et al., 2015; Jewkes et al., 2006; Lou et al., 2004; Meekers, 2000; Pandey et al., 2016; Speizer et al., 2001). Two studies were conducted at community sites and in clinics (Kaljee et al., 2005; Kanesathasan et al., 2008). One study took place in a combination of school and clinics (Mmbaga et al., 2017), and three in community (Diop et al., 2004), and school (Cowan et al., 2010; Ross et al., 2007).

###### Participants

All the studies had participants from either low income, lower middle income or upper middle income countries (Baird et al., 2010; Cabezón et al., 2005; Cowan et al., 2010; Daniel et al., 2008; Diop et al., 2004; Duflo et al., 2015; Dupas, 2011; Erulkar & Muthengi, 2007; Gallegos et al., 2008; Handa et al., 2015; James et al., 2005; Jewkes et al., 2006; Kaljee et al., 2005; Kanesathasan et al., 2008; Klepp et al., 1997; Lou et al., 2004; Martiniuk et al., 2003; Meekers, 2000; Mmbaga et al., 2017; Okonofua et al., 2003; Pandey et al., 2016; Ross et al., 2007; Shuey et al., 1999; Speizer et al., 2001; Walker et al., 2006; Ybarra et al., 2013). The minimum population size was 366 participants in Ybarra et al. (2013) and the maximum population was 19,289 participants in Duflo et al. (2015). The minimum included age was 10 years (Diop et al., 2004; Erulkar & Muthengi, 2007) and the maximum was “30 years and older” in Kanesathasan et al. (2008).

###### Interventions

Sixteen studies had education on sexual health alone as the intervention (Cabezón et al., 2005; Cowan et al., 2010; Daniel et al., 2008; Diop et al., 2004; Dupas, 2011; Erulkar & Muthengi, 2007; Gallegos et al., 2008; James et al., 2005; Jewkes et al., 2006; Kaljee et al., 2005; Klepp et al., 1997; Martiniuk et al., 2003; Mmbaga et al., 2017; Pandey et al., 2016; Shuey et al., 1999; Speizer et al., 2001; Ybarra et al., 2013). In six studies, education on sexual health was combined with other strategies; provision of contraceptives (Lou et al., 2004; Meekers, 2000; Walker et al., 2006), peer referrals to health care providers and training of health care providers (Okonofua et al., 2003), training of health workers and peer condom marketing (Ross et al., 2007), referrals, family members' education and improvement of contraceptive services (Pandey et al., 2016), skills training, referrals to micro savings and credit groups and training of health care providers (Kanesathasan et al., 2008), development of youth partnership groups, and education subsidies (Duflo et al., 2015). Cash transfers were given in two studies (Baird et al., 2010; Handa et al., 2015); they were conditional in one study (Baird et al., 2010), and unconditional in the other (Handa et al., 2015).

###### Comparison groups

In 25 studies, the comparison group received no intervention (Baird et al., 2010; Cabezón et al., 2005; Cowan et al., 2010; Daniel et al., 2008; Diop et al., 2004; Duflo et al., 2015; Dupas, 2011; Erulkar & Muthengi, 2007; Gallegos et al., 2008; Handa et al., 2015; James et al., 2005; Jewkes et al., 2006; Kaljee et al., 2005; Kanesathasan et al., 2008; Klepp et al., 1997; Lou et al., 2004; Martiniuk et al., 2003; Meekers, 2000; Mmbaga et al., 2017; Okonofua et al., 2003; Pandey et al., 2016; Ross et al., 2007; Shuey et al., 1999; Speizer et al., 2001; Walker et al., 2006; Ybarra et al., 2013). However, in Cowan et al. (2010) the participants in the control group received the same intervention after follow up data had been collected.

##### Optimising interpregnancy intervals

###### Included studies

We included four studies in this comparison which involved a total of 15,718 participants. Three were quasi experimental studies (one CBA and two natural experiments) (Baqui et al., 2018; Daniel et al., 2008; Pandey et al., 2016) and one RCT (Zhu et al., 2009).

###### Outcomes

The primary outcome reported by one of the included studies is unintended pregnancy (Zhu et al., 2009).

The secondary outcomes reported include changes in knowledge and attitudes about the risk of unintended pregnancies (Pandey et al., 2016), initiation of sexual intercourse (Pandey et al., 2016), use of birth control methods (Daniel et al., 2008; Pandey et al., 2016; Zhu et al., 2009), and abortion (Zhu et al., 2009).

###### Settings

All four studies were conducted in Asia: one in Bangladesh (Baqui et al., 2018), one in China (Zhu et al., 2009), and two in India (Daniel et al., 2008; Pandey et al., 2016). All the studies were conducted in community settings.

###### Participants

Of the four studies (Baqui et al., 2018; Daniel et al., 2008; Pandey et al., 2016; Zhu et al., 2009), the minimum population size was 2336 participants in Zhu et al. (2009) and the in Pandey et al. (2016).

###### Interventions

Education about sexual health and family planning was the main aspect of the intervention in three studies (Daniel et al., 2008; Pandey et al., 2016; Zhu et al., 2009). There were also additional elements contraceptives supplies, family and parental support and provision of youth friendly health system in Pandey et al. (2016), and referrals, free provision of contraceptive materials, and involvement of the male partner in Zhu et al. (2009). The postpartum family planning along with maternal and newborn care for birth spacing was the intervention in one study (Baqui et al., 2018).

###### Comparison group

In Daniel et al. (2008), and Pandey et al. (2016), the intervention arm was compared to a controlled, nonrecipient arm. While in Zhu et al. (2009), the difference between intervention arm also received contraceptives and involved male partner in education.

###### Timing of intervention

The timing of the intervention was preconception in the included studies (Daniel et al., 2008; Pandey et al., 2016; Zhu et al., 2009). In two studies (Daniel et al., 2008; Pandey et al., 2016) pregnant participants were also included. In one study (Baqui et al., 2018) only pregnant adolescents were included.

##### Periconceptional folic acid

###### Included studies

We included five studies with a total of 255,212 women in this comparison. The included studies mainly focused on maternal outcomes and few reported outcomes related to neonatal health. Sample sizes ranged from 140 (Rosenthal 2008) to 247,831 (Berry et al., 1999). There were two RCTs (Li, 2014; Rosenthal 2008) and three quasi experimental natural experiments (two natural experiments and one CBA) (Berry et al., 1999; Vergel et al., 1990; Wehby et al., 2012).

###### Outcomes

The primary outcomes reported were NTD (Berry et al., 1999; Vergel et al., 1990), and stillbirth (Wehby et al., 2012). The secondary outcomes reported were miscarriage (Wehby et al., 2012), serum folate (Rosenthal 2008), adherence to folic acid or iron folic acid supplementation (Wehby et al., 2012) and other congenital anomalies (Wehby et al., 2012).

###### Settings

Of the five included studies, two took place in Asia: China (Berry et al., 1999; Li, 2014), one study took place in North America; in Honduras (Rosenthal, 2008), and one in South America: Brazil (Wehby et al., 2012). One study in Carribean: Cuba (Vergel et al., 1990).

###### Participants

All participants were nonpregnant women with ages ranging from 16 to 49 years. One study (Vergel et al., 1990) had women with a history of a child with NTDs. Li (2014) recruited participants who met the inclusion criteria at the rural and the urban maternal health centre from four districts of the Tongliao city. Berry et al. (1999) included participants from Hebei (a northern province of China) and Zhejiang and Jiangsu (two southern provinces). Women who underwent premarital examination during a specific time frame were identified and given folic acid to consume till the end of the first trimester of their pregnancy. Of these, women with pregnancies where NTD could be confirmed or ruled out were included in this study. Rosenthal (2008) was carried out in factories in Choloma, Honduras. An Information fair was held in one of the factories and all the women between the ages of 18–49 who volunteered were recruited. Vergel et al. (1990) included patients registered at the Provincial Genetic Department in Havana City who had had a previous pregnancy with NTD. Wehby et al. (2012) was conducted at six craniofacial clinics in Brazil. Women who themselves had an oral cleft or had a pregnancy complicated with oral clefts were asked to enrol in the study.

###### Intervention

**Micronutrient composition**

Five studies (Berry et al., 1999; Li, 2014; Rosenthal, 2008; Vergel et al., 1990; Wehby et al., 2012) used folic acid supplementation alone

**Dose of folic acid**

Three studies supplemented women with 0.4 mg of folic acid per day (Berry et al., 1999; Li, 2014; Wehby et al., 2012) while in the remaining studies, women were supplemented with 1 mg (Rosenthal, 2008), 4 mg (Wehby et al., 2012) and 5 mg (Vergel et al., 1990) of folic acid daily. One study also had a 5 mg weekly arm (Rosenthal, 2008). Wehby et al. (2012) had multiple intervention arms where one group was given 0.4 mg and other arm was given 4 mg.

**Folate compound**

All studies used folic acid.

**Regimen**

In all studies, participants were supplemented daily. In one study, there was an additional arm in which a group of participants were also supplemented weekly (Rosenthal 2008).

The supplementation started before pregnancy and continued until first trimester.

**Timing of supplementation**

All studies gave the supplements in the periconceptional period except two, which were during preconception only (Li, 2014; Rosenthal 2008).

###### Comparison group

Two of the studies had two study arms (Rosenthal, 2008; Wehby et al., 2012). Two studies first divided the participants according to region (Berry et al., 1999) or ethnicity (Li, 2014). Berry et al. (1999) further divided the participants according to pattern of use of folic acid, that is, periconceptional use, late use, early discontinuation or no use. Li (2014) subdivided according to their assigned supplement, that is, folic acid + milk, only folic acid, only milk, and control. We compared only folic acid with control in our analysis since the usage of milk was out of the scope of this study.

One study (Vergel et al., 1990) had three arms: fully supplemented (received full course of folic acid supplementation), partially supplemented (received folic acid but not the full regimen) and un supplemented participants.

##### Periconceptional iron folic acid

###### Included studies

We included 10 studies with a total of 8439 participants. All of the studies reported maternal outcomes. Sample size ranged from 406 (Shobha & Sharada, 2003) to 3616 (Soekarjo et al., 2004). There were nine RCTs (Agarwal et al., 2003; Ahmed et al., 2001; Februhartanty et al., 2002; Gilgen, 2001; Hall et al., 2002; Muro et al., 1999; Shah & Gupta, 2002; Shobha & Sharada, 2003; Soekarjo et al., 2004) and one quasi experimental natural experiment (CBA) (Kanani 2000).

###### Outcomes

The primary outcome reported was anaemia (Agarwal et al., 2003; Ahmed et al., 2001; Gilgen, 2001; Hall et al., 2002; Muro et al., 1999; Shah & Gupta, 2002) The secondary outcomes reported were adverse effects (Gilgen, 2001).

###### Settings

Of the 10 included studies, eight took place in Asia: two in Bangladesh (Ahmed et al., 2001; Gilgen, 2001), two in India (Agarwal et al., 2003; Shobha & Sharada, 2003), three Indonesia (Februhartanty et al., 2002; Kanani, 2000; Soekarjo et al., 2004), and one in Nepal (Shah & Gupta, 2002). Two studies took place in Africa: one in Mali (Hall et al., 2002), and one in Tanzania (Muro et al., 1999).

Seven studies were conducted in schools (Agarwal et al., 2003; Februhartanty et al., 2002; Hall et al., 2002; Muro et al., 1999; Shah & Gupta, 2002; Shobha & Sharada, 2003; Soekarjo et al., 2004). Soekarjo et al. (2004) also included home intervention during school holidays. Of the remaining studies, one was conducted in garment factories (Ahmed et al., 2001), two in community settings (Gilgen, 2001; Kanani, 4).

###### Participants

All of the studies supplemented participants with iron‐folic acid in the preconception period.

###### Interventions

**Micronutrient composition:** Several different dosages of iron and folic acid were used in these studies. Ahmed et al. (2001) supplemented 120 mg iron and 3.5 mg folic acid, Agarwal et al. (2003) supplemented 100 mg iron and folate 500 µg two groups in daily and weekly manner, Februhartanty et al. (2002), Hall et al. (2002), and Soekarjo et al. (2004) supplemented 60 mg elemental iron and 0.25 mg folic on a weekly basis, Kanani (2000) supplemented 60 mg of elemental iron 0.5 mg folic acid per day, Muro et al. (1999) supplemented 65 mg of elemental iron with 0.25 mg of folic acid weekly, Shah and Gupta (2002) supplemented 350 mg iron and 1.5 mg folic acid once daily or weekly, Shobha and Sharada (2003) supplemented 60 mg iron and 0.5 mg folic acid daily or twice weekly, and Gilgen 2001 supplemented 200 mg ferrous fumarate and 200 mg folic acid on a weekly basis.

**Regimen:** Kanani (2000) had daily supplementation group. Ahmed et al. (2001), Februhartanty et al. (2002), Hall et al. (2002), Gilgen (2001), Muro et al. (1999) and Soekarjo et al. (2004) had weekly arms. Agarwal et al. (2003), Shah and Gupta (2002) had a daily arm as well as a weekly one. Shobha and Sharada (2003) had daily and twice weekly supplementation groups.

**Timing of supplementation:** All studies provided the supplementation during the preconception period.

**Duration of supplementation:** Muro et al. (1999) supplemented for 8 weeks, Hall et al. (2002) and Shah and Gupta (2002) supplemented for 10 weeks, seven studies provided supplementation for more than 12 weeks (Agarwal et al., 2003; Ahmed et al., 2001; Februhartanty et al., 2002; Gilgen, 2001; Kanani, 2000; Shobha & Sharada, 2003; Soekarjo et al., 2004).

###### Comparison

All these studies compared iron‐folic acid supplementation with placebo.

#### 5.1.3 Excluded studies

##### Refer to characteristics of excluded studies

###### Delaying pregnancy

We excluded 105 studies in total: 79 because they were from high income countries; 11 because they had data that was not disaggregated by sex (Agha et al., 2004; Doubova et al., 2017; Fawole et al., 1999; Jemmott III, 2010; Kim et al., 2001; Kinsler et al., 2004; Kyrychenko 2006; Mathews et al., 2016; Mba et al., 2007; Munodawafa et al., 1995; Taylor et al., 2001); eight because they had no specific intervention to delay pregnancy (Antunes et al., 2002; Baptiste, 2005; Decat et al., 2015; Gaughran, 2013; Kamali et al., 2002; Pereira et al., 2017; Sebastian et al., 2012); three because they targeted participants that were not relevant to this review (García et al., 2012; Marcell et al., 2013; Villarruel, 2008); three because they had no control group to compare the interventional group to (Brieger et al., 2001; Magnani et al., 2005; Ozcebe et al., 2004); one because it did not directly measure the impact of the intervention (Meekers et al., 2005); one because it focused on childbearing (Palermo et al., 2016) and one, because it was related to the preintervention status of participants (Cowan et al., 2008).

###### Optimising interpregnancy intervals

We excluded 11 studies: seven because they were from high income countries (Black et al., 2006; Kan, 2012; Quinlivan et al., 2003; Sims & Luster, 2002; Solomon & Liefeld, 1998; Wagner & Clayton, 1999; Wiggins et al., 2005); one because the article was originally in Spanish and only a rough English translation could be obtained which made it not possible to interpret the paper clearly; moreover the tables were still in Spanish (Rocha, 2004); one compared one type of contraceptive with another (Hubacher 2012), and one because there was no intervention relevant to prolong pregnancy (Bandiera et al., 2012), rather it provided life skills and vocational skills to the participants which did not serve the specific purpose of this review. Drayton (2000) only had abstract and Rosenberg et al. (2015) was a cohort study.

###### Periconceptional folic acid

We excluded 12 studies: four because they were from high income countries (Czeizel and Dobo, 1994; Laurence et al., 1981; Kirke 1992; MRC, 1991); three because of flour fortification (Calvo & Biglieri, 2008; López‐Camelo et al., 2005; Sayed et al., 2008); two because all arms received same dose of folic acid (Angeles‐Adgeppa et al., 2005; Gunaratna et al., 2015); one because the folic acid use was during pregnancy only (Manizheh et al., 2009); one because it compared folic acid along with other micronutrients to a control group (Chen et al., 2008) and one because folic acid dosage was not mentioned and none of the outcomes were of interest to this review (Westphal et al., 2004).

###### Periconceptional Iron folic acid

Twenty‐seven studies were excluded, of which seven only had iron supplementation without folic acid (Beasley 2000; Gonzalez‐Rosendo, 2002; Kianfar et al., 2000; Leenstra et al., 2009; Lopes, 1999; Mozaffari‐Khosravi et al., 2010; Zavaleta et al., 2000); nine did not have any or an appropriate comparison group (Ahmed et al., 2005, 2010; Angeles‐Agdeppa et al., 1997; Crape et al., 2005; Deshmukh, 2008; Dongre et al., 2011; Gunaratna et al., 2015; Horjus et al., 2005; Jayatissa, 1999; Kotecha et al., 2009; Nguyen et al., 2012; Pasricha, 2009; Tee, 1999; Vyas et al., 2010); three studies were not segregated by gender (Roschnik et al., 2004; Taylor et al., 2001; Yusoff et al., 2012); two studies were summaries of others (Aguayo et al., 2013; Roschnik et al., 2008); and one looked at post pregnancy intervention (Nguyen et al., 2012).

### 5.2 Risk of bias in included studies

#### 5.2.1 Randomised controlled trials

##### Delaying pregnancy

There were 14 RCTs related to delaying pregnancy (Baird et al., 2010; Cabezón et al., 2005; Cowan et al., 2010; Duflo et al., 2015; Gallegos et al., 2008; Handa et al., 2015; Jewkes et al., 2006; Kaljee et al., 2005; Martiniuk et al., 2003; Mmbaga et al., 2017; Okonofua et al., 2003; Ross et al., 2007; Walker et al., 2006; Ybarra et al., 2013).

##### Optimising interpregnancy intervals

There was one RCT (Zhu et al., 2009) related to prolonging inter‐pregnancy intervals.

##### Periconceptional folic acid

There were two RCTs (Li, 2014; Rosenthal 2008).

##### Periconceptional iron folic acid

There were nine RCTs (Agarwal et al., 2003; Ahmed et al., 2001; Februhartanty et al., 2002; Gilgen, 2001; Hall et al., 2002; Muro et al., 1999; Shah & Gupta, 2002; Shobha & Sharada, 2003; Soekarjo et al., 2004).

#### Allocation (selection bias)

##### Delaying pregnancy

*Sequence generation*: Adequate randomisation was done in nine studies (Cabezón et al., 2005; Duflo et al., 2015; Handa et al., 2015; Jewkes et al., 2006; Martiniuk et al., 2003; Mmbaga et al., 2017; Ross et al., 2007; Walker et al., 2006; Ybarra et al., 2013), due to which the risk of bias was low. Methods used included simple balloting (Cabezón et al., 2005), computer generated number sequences (Handa et al., 2015; Jewkes et al., 2006; Mmbaga et al., 2017) and coin tossing (Martiniuk et al., 2003). Handa et al. (2015) employed lottery generation of for locations and then further, households were selected via computer generated number sequences.

The randomisation method was not clearly mentioned in five studies (Baird et al., 2010; Cowan et al., 2010; Gallegos et al., 2008; Kaljee et al., 2005; Okonofua et al., 2003), making the risk of bias unclear.

*Allocation concealment:* The risk for bias for allocation concealment was low in five studies (Cowan et al., 2010; Handa et al., 2015; Jewkes et al., 2006; Mmbaga et al., 2017; Ybarra et al., 2013) and unclear in nine others (Baird et al., 2010; Cabezón et al., 2005; Duflo et al., 2015; Gallegos et al., 2008; Kaljee et al., 2005; Martiniuk et al., 2003; Okonofua et al., 2003; Ross et al., 2007; Walker et al., 2006).

##### Optimising interpregnancy intervals

*Sequence generation*: One study (Zhu et al., 2009) adequately randomised participants; this was achieved by coin tossing which was done by a neutral party who was not involved with the study at one of the research centres.

*Allocation concealment:* In the same study, the risk of bias was low because the unit of allocation was randomised clusters (Zhu et al., 2009).

##### Periconceptional folic acid

*Sequence generation*: One study adequately randomised the participants (Rosenthal, 2008), using a data centre to make a randomisation sequence. One study did not mention the method of randomisation (Li, 2014).

*Allocation concealment: One* study adequately concealed allocation (Rosenthal, 2008) as they reported using identical pills or packaging. Li (2014) did not mention any method of allocation concealment.

##### Periconceptional iron folic acid

*Sequence generation:* Allocation sequence was adequately generated in two studies (Gilgen, 2001; Hall et al., 2002). All of them used a computer generated programme. One study was at high risk (Muro et al., 1999). Muro et al. (1999) added a school which was supposed to receive the intervention into the nonintervention group because the parents did not approve of the intervention. Six studies did not mention method of sequence generation (Agarwal et al., 2003; Ahmed et al., 2001; Februhartanty et al., 2002; Shah & Gupta, 2002; Shobha & Sharada, 2003; Soekarjo et al., 2004).

*Allocation concealment: Four* studies reported adequate allocation concealment (Agarwal et al., 2003; Ahmed et al., 2001; Hall et al., 2002; Soekarjo et al., 2004). Three of these studies were randomised at cluster level and so, risk of selection bias was considered unlikely (Agarwal et al., 2003; Hall et al., 2002; Soekarjo et al., 2004). In one (Ahmed et al., 2001), the code was not broken till the end of the study or till all the data had been entered in the computer. Muro et al. (1999) did not perform allocation concealment. The rest of the studies had an unclear method or did not perform allocation concealment (Februhartanty et al., 2002; Gilgen, 2001; Shah & Gupta, 2002; Shobha & Sharada, 2003).

#### 5.2.2 Blinding (performance bias and detection bias)

##### Delaying pregnancy

**Blinding of participants and personnel:** Three RCTs (Okonofua et al., 2003; Ross et al., 2007; Walker et al., 2006) were deemed at high risk of bias with regards to blinding of participants/personnel. Eight studies (Baird et al., 2010; Cabezón et al., 2005; Cowan et al., 2010; Duflo et al., 2015; Handa et al., 2015; Jewkes et al., 2006; Kaljee et al., 2005; Mmbaga et al., 2017) were at low risk due to adequate blinding or because blinding was not required due to the type of intervention occurring. Three RCTs which did not mention blinding of participants/personnel distinctly were at unclear risk of bias (Gallegos et al., 2008; Martiniuk et al., 2003; Ybarra et al., 2013).

**Blinding for outcome assessors:** Two RCTs (Jewkes et al., 2006; Ross et al., 2007) had low risk of bias for blinding of outcome assessment while most of them were at unclear risk as this was not specifically mentioned in 12 articles (Baird et al., 2010; Cabezón et al., 2005; Cowan et al., 2010; Duflo et al., 2015; Gallegos et al., 2008; Handa et al., 2015; Kaljee et al., 2005; Klepp et al., 1997; Martiniuk et al., 2003; Mmbaga et al., 2017; Okonofua et al., 2003; Walker et al., 2006).

##### Optimising interpregnancy intervals

**Blinding of participants and personnel:** There was high risk of bias concerning blinding in one study as the interviewers were not blinded to the interventions that the women in different arms of the study would be receiving (Zhu et al., 2009).

**Blinding of outcome assessors:** There was high risk of bias concerning blinding in one study as the assessors were not blinded to the interventions that the women in different arms of the study would be receiving (Zhu et al., 2009).

##### Periconceptional folic acid

**Blinding of participants and personnel:** One study had adequate blinding of participants and personnel (Rosenthal 2008) while one was at high risk of bias because participants and personnel were not blinded (Li, 2014).

**Blinding of outcome assessor:** Rosenthal (2008) performed adequate blinding and was at low risk of bias. One study had insufficient information to permit judgement (Li, 2014).

##### Periconceptional iron folic acid

**Blinding of participants and personnel:** Out of nine studies, three performed adequate blinding of participants and personnel (Ahmed et al., 2001; Februhartanty et al., 2002; Gilgen, 2001). Five studies were at high risk of bias due to inadequate blinding (Agarwal et al., 2003; Muro et al., 1999; Shah & Gupta, 2002; Shobha & Sharada, 2003; Soekarjo et al., 2004). One study did not mention blinding of participants or personnel (Hall et al., 2002).

**Blinding of outcome assessors:** Six of the studies were at low risk of bias. Three had objective outcomes which were not affected by outcome assessors knowing the intervention (Agarwal et al., 2003; Ahmed et al., 2001; Soekarjo et al., 2004). Six studies did not mention blinding of outcome assessor and hence, had an unclear risk of bias (Februhartanty et al., 2002; Gilgen, 2001; Hall et al., 2002; Muro et al., 1999; Shah & Gupta, 2002; Shobha & Sharada, 2003).

#### 5.2.3 Incomplete outcome data (attrition bias)

##### Delaying pregnancy

The issue of incomplete outcome data were not addressed adequately in one study (Walker et al., 2006) putting them at an unclear risk of bias. One study was at high risk of attrition bias due to significant loss to follow up from both intervention and control groups (Cowan et al., 2010).

Studies that were at low risk addressed incomplete outcome data adequately in 12 studies (Baird et al., 2010; Cabezón et al., 2005; Duflo et al., 2015; Gallegos et al., 2008; Handa et al., 2015; Jewkes et al., 2006; Kaljee et al., 2005; Martiniuk et al., 2003; Mmbaga et al., 2017; Okonofua et al., 2003; Ross et al., 2007; Ybarra et al., 2013). In Cabezón et al. (2005) and Gallegos et al. (2008), the missing outcome data were balanced across groups. In Handa et al. (2015) the effect of attrition was accounted for.

##### Optimising interpregnancy intervals

This issue was addressed reasonably well due to low attrition rates (Zhu et al., 2009).

##### Periconceptional folic acid

In two studies (Li, 2014; Rosenthal 2008) incomplete outcome data were matched across groups.

##### Periconceptional iron folic acid

Two studies did not mention if incomplete outcome data were adequately addressed (Gilgen, 2001; Shobha & Sharada, 2003). Imbalanced losses were mentioned in one study (Ahmed et al., 2001). In the rest of the studies, the missing outcome data were matched across groups (Agarwal et al., 2003; Februhartanty et al., 2002; Hall et al., 2002; Muro et al., 5; Shah & Gupta, 2002; Soekarjo et al., 2004).

#### 5.2.4 Selective reporting (reporting bias)

##### Delaying pregnancy

Eleven of the studies had unclear risk of bias as there was insufficient evidence to disregard the notion of selective reporting (Baird et al., 2010; Duflo et al., 2015; Gallegos et al., 2008; Handa et al., 2015; Jewkes et al., 2006; Kaljee et al., 2005; Martiniuk et al., 2003; Okonofua et al., 2003; Ross et al., 2007; Walker et al., 2006; Ybarra et al., 2013). Two study was deemed at low‐risk due to the outcomes being pre‐specified in another paper (Cowan et al., 2010) and protocol (Mmbaga et al., 2017).

##### Optimising interpregnancy intervals

There were no available protocols to determine whether selective reporting occured.

##### Periconceptional folic acid

In two studies (Li, 2014; Rosenthal 2008) assessing selective reporting bias was difficult as we did not have access to study protocols and therefore these were rated as unclear.

##### Periconceptional iron folic acid

Assessing selective reporting was difficult as we did not have study protocols for the majority of the studies, therefore these were deemed unclear for risk of selective reporting.

#### 5.2.5 Other potential sources of bias

##### Delaying pregnancy

Eleven studies were free from other sources of bias (Cabezón et al., 2005; Cowan et al., 2010; Duflo et al., 2015; Gallegos et al., 2008; Handa et al., 2015; Jewkes et al., 2006; Kaljee et al., 2005; Okonofua et al., 2003; Ross et al., 2007; Walker et al., 2006; Ybarra et al., 2013). The remaining three RCTs had an unclear risk of bias (Baird et al., 2010; Martiniuk et al., 2003; Mmbaga et al., 2017). The possibility of bias was due to baseline differences between participants (Baird et al., 2010; Martiniuk et al., 2003) and measurement bias (Mmbaga et al., 2017).

##### Prolonging interpregnancy intervals

There was a possible risk of bias as baseline outcome differences existed between the two interventional arms of the study (Zhu et al., 2009).

##### Periconceptional folic acid

In one study (Rosenthal 2008), there was a different time lag between last pill taken and blood drawn for the two groups; unmetabolized folic acid in the blood and dietary folic acid intake was also not taken into account. Li (2014) appear free from other bias.

##### Periconceptional iron folic acid

Two of the studies were at high risk for sources of bias (Februhartanty et al., 2002; Shah & Gupta, 2002). In Februhartanty et al. (2002), there was a higher prevalence of anaemia in the weekly supplemented group. Only the weekly group was supervised in the Shah and Gupta (2002) study. In one study it was not clear if there was a bias (Ahmed et al., 2001). In Ahmed et al. (2001), there was variability in when the supplements were administered depending on the factory (before or after a meal etc).

##### Quasi experimental studies

###### Delaying pregnancy

There were 12 quasi experimental studies related to delaying pregnancy (Daniel et al., 2008; Diop et al., 2004; Dupas, 2011; Erulkar & Muthengi, 2007; James et al., 2005; Kanesathasan et al., 2008; Klepp et al., 1997; Lou et al., 2004; Meekers, 2000; Pandey et al., 2016; Shuey et al., 1999; Speizer et al., 2001).

###### Optimising interpregnancy intervals

There were three quasi experimental studies related to prolonging inter‐pregnancy intervals (Baqui et al., 2018; Daniel et al., 2008; Pandey et al., 2016).

###### Periconceptional folic acid

There were three quasi experimental studies (Berry et al., 1999; Vergel et al., 1990; Wehby et al., 2012).

###### Periconceptional iron folic acid

There one quasi experimental study (Kanani 2000).

##### Was the allocation sequence adequately generated?

###### Delaying pregnancy

Adequate randomisation was done in two studies (Kanesathasan et al., 2008; Shuey et al., 1999), due to which the risk of bias was low. The randomisation method was not mentioned in five studies (Diop et al., 2004; Dupas, 2011; James et al., 2005; Klepp et al., 1997; Pandey et al., 2016), making the risk of bias unclear.The allocation sequence was not adequately generated in seven studies (Daniel et al., 2008; Erulkar & Muthengi, 2007; Lou et al., 2004; Meekers, 2000; Speizer et al., 2001), making their risk of bias high.

###### Optimising interpregnancy intervals

One study did not clearly state the method of randomisation (Pandey et al., 2016). One study was deemed to have inadequate sequence generation as an index household was chosen in a quasi‐random manner from each selected village, and the investigator moved from household to household, in a predetermined direction, interviewing eligible women (Daniel et al., 2008). One study did not employ randomisation methods. In one study (Baqui et al., 2018) there was clearly no randomisation done.

###### Periconceptional folic acid

One study (Wehby et al., 2012) adequately generated allocation sequence. Two studies (Berry et al., 1999; Vergel et al., 1990) were at high risk of bias since allocation sequence was not adequately generated.

###### Periconceptional iron folic acid

In Kanani 2000 method of sequence generation is not described.

##### Was the allocation adequately concealed?

###### Delaying pregnancy

The risk for bias for allocation concealment was low in six studies (Dupas, 2011; James et al., 2005; Lou et al., 2004; Meekers, 2000; Pandey et al., 2016; Speizer et al., 2001) and unclear in six others (Daniel et al., 2008; Diop et al., 2004; Erulkar & Muthengi, 2007; Kanesathasan et al., 2008; Klepp et al., 1997; Shuey et al., 1999).

###### Optimising interpregnancy intervals

In two studies the risk of bias for allocation concealment was unclear as it was not mentioned in the respective text (Baqui et al., 2018; Daniel et al., 2008). The risk of bias was low in two studies. In one it was because the unit of allocation consisted of individual villages allowing adequate allocation concealment (Pandey et al., 2016).

###### Periconceptional folic acid

Two studies were at low risk of bias: allocation was according to provinces in Berry et al. (1999) while in Wehby et al. (2012) allocation was adequately concealed. Vergel et al. (1990) was at high risk of bias since allocation was not concealed and analysis was done according to compliance.

###### Periconceptional iron folic acid

It is unclear if allocation was adequately concealed in Kanani (2000).

##### Were baseline outcome measurements similar?

###### Delaying pregnancy

Baseline outcomes were similar across groups in four studies (Dupas, 2011; James et al., 2005; Klepp et al., 1997; Lou et al., 2004). They were not significantly similar in six studies (Daniel et al., 2008; Diop et al., 2004; Erulkar & Muthengi, 2007; Meekers, 2000; Shuey et al., 1999; Speizer et al., 2001). The remaining two studies did not have sufficient information to make a judgement on the similarity of baseline outcomes between groups (Kanesathasan et al., 2008; Pandey et al., 2016).

###### Optimising interpregnancy intervals

There was insufficient information to make a judgement regarding this in Pandey et al. (2016). There was a significant difference in baseline outcomes between groups in one study (Daniel et al., 2008). In one study (Baqui et al., 2018), baseline outcomes were similar.

###### Periconceptional folic acid

Out of three studies, one had similar baseline outcomes (Wehby et al., 2012), one did not have any baseline outcome measured (Berry et al., 1999), and one did not have similar baseline outcome measurements (Vergel et al., 1990).

###### Periconceptional iron folic acid

In one of the studies baseline outcome measurements were similar (Kanani, 2000)

##### Were baseline characteristics similar?

###### Delaying pregnancy

Baseline characteristics were similar across groups in six studies (Daniel et al., 2008; Diop et al., 2004; Dupas, 2011; Kaljee et al., 2005; Klepp et al., 1997; Shuey et al., 1999). They were not significantly similar in six studies (Erulkar & Muthengi, 2007; James et al., 2005; Lou et al., 2004; Meekers, 2000; Pandey et al., 2016; Speizer et al., 2001). The remaining study did not have sufficient information to make a judgement on the similarity of baseline characteristics between groups (Kanesathasan et al., 2008).

###### Optimising interpregnancy intervals

Baseline characteristics were similar in one study (Daniel et al., 2008). There were significant differences between groups regarding these in two studies (Baqui et al., 2018; Pandey et al., 2016).

###### Periconceptional folic acid

Baseline characteristics were similar in one of the studies (Wehby et al., 2012). Vergel et al. (1990) did not provide any baseline characteristics. In Berry et al. (1999), baseline characteristics of the women in the folic acid group varied from those who did not take supplementation.

###### Periconceptional iron folic acid

The baseline characteristics were similar Kanani (2000).

##### Were incomplete outcome data adequately addressed?

###### Delaying pregnancy

The issue of incomplete outcome data were not addressed adequately in 10 studies (Daniel et al., 2008; Diop et al., 2004; Dupas, 2011; Erulkar & Muthengi, 2007; Kanesathasan et al., 2008; Lou et al., 2004; Meekers, 2000; Pandey et al., 2016; Shuey et al., 1999; Speizer et al., 2001) putting them at an unclear risk of bias. The two studies that were at low risk addressed incomplete outcome data adequately (James et al., 2005; Klepp et al., 1997).

###### Optimising interpregnancy intervals

There was either unclear or low risk of bias perceived regarding incomplete outcome data. There was insufficient information available to make an adequate assessment in two studies (Daniel et al., 2008; Pandey et al., 2016). The remaining study addressed this issue reasonably well due to low attrition rates (Baqui et al., 2018).

###### Periconceptional folic acid

In one study incomplete outcome data were matched across groups (Berry et al., 1999). Vergel et al. (1990) did not mention if no participants were lost to follow up. Wehby et al., 2012 had a substantial attrition rate. In the 0.4 mg folic acid supplementation group, a total of 1106 withdrew or were lost to follow up. A similar number of women (1093) dropped out of the study for the 4.0 mg folic acid supplementation group.

###### Periconceptional iron folic acid

Kanani 2000 does not mention incomplete outcome data.

##### Was knowledge of the allocated interventions adequately prevented during the study?

###### Delaying pregnancy

There was unclear risk for all of the studies relevant to this question as this was not distinctly mentioned in the respective texts (Daniel et al., 2008; Diop et al., 2004; Dupas, 2011; Erulkar & Muthengi, 2007; James et al., 2005; Kanesathasan et al., 2008; Klepp et al., 1997; Lou et al., 2004; Meekers, 2000; Pandey et al., 2016; Shuey et al., 1999; Speizer et al., 2001).

###### Optimising interpregnancy intervals

There was an unclear risk of bias with regards to whether knowledge of the allocated interventions adequately prevented in three studies (Baqui et al., 2018; Daniel et al., 2008; Pandey et al., 2016).

###### Periconceptional folic acid

All three studies were at low risk of bias: two studies adequately prevented knowledge of allocated interventions (Berry et al., 1999; Wehby et al., 2012) and one study had objective outcomes which were not affected by knowledge of allocated interventions (Vergel et al., 1990).

###### Periconceptional iron folic acid

In Kanani 2000, the outcomes were objectives and so, unlikely to be influenced by blinding.

##### Was the study adequately protected against contamination?

###### Delaying pregnancy

With regards to contamination, adequate measures were taken in 10 studies (Daniel et al., 2008; Dupas, 2011; Erulkar & Muthengi, 2007; James et al., 2005; Klepp et al., 1997; Lou et al., 2004; Meekers, 2000; Pandey et al., 2016; Shuey et al., 1999; Speizer et al., 2001). There was insufficient data to make a judgement in one case (Kanesathasan et al., 2008). There was a high risk of bias with regards to the possibility of contamination in one study (Diop et al., 2004). In this study, the control group partially received some component of the intervention.

###### Optimising interpregnancy intervals

Three studies showed that adequate measures against contamination had been taken (Baqui et al., 2018; Daniel et al., 2008; Pandey et al., 2016).

###### Periconceptional folic acid

In one study (Berry et al., 1999), allocation was by province and county and hence, therefore the study was adequately protected against contamination. Two studies (Vergel et al., 1990; Wehby et al., 2012) did not mention any measures to prevent contamination.

###### Periconceptional iron folic acid

The study was protected against contamination (Kanani & Poojara, 2000) and had allocation by community while one study had two different intervention arms (iron‐folic acid supplementation and leaf concentrate) and hence it was unlikely that there was contamination.

##### Was the study free from selective outcome reporting?

###### Delaying pregnancy

Eight of the studies had low risk of bias when there was sufficient evidence to disregard the notion of selective reporting (Diop et al., 2004; Dupas, 2011; Erulkar & Muthengi, 2007; Kanesathasan et al., 2008; Lou et al., 2004; Pandey et al., 2016; Shuey et al., 1999; Speizer et al., 2001) while four had unclear risk of bias when there was not (Daniel et al., 2008; James et al., 2005; Klepp et al., 1997; Meekers, 2000).

###### Optimising interpregnancy intervals

Three studies were at low risk of selective outcome reporting (Baqui et al., 2018; Daniel et al., 2008; Pandey et al., 2016).

###### Periconceptional folic acid

In one study, there is no evidence that outcomes were selectively reported (Berry et al., 1999) while in two studies it is unclear if the study is free from selective outcome reporting (Vergel et al., 1990; Wehby et al., 2012). Wehby et al. (2012) mentioned all the outcomes though did not extensively discuss them.

###### Periconceptional iron folic acid

The outcomes mentioned in the methods section were reported in the results (Kanani 2000).

##### Was the study free from other risks of bias?

###### Delaying pregnancy

Ten studies were free from other sources of bias (Daniel et al., 2008; Dupas, 2011; Erulkar & Muthengi, 2007; James et al., 2005; Kanesathasan et al., 2008; Klepp et al., 1997; Lou et al., 2004; Meekers, 2000; Pandey et al., 2016; Speizer et al., 2001). Two quasi experimental studies had an unclear risk of bias (Shuey et al., 1999) or a high risk of bias (Diop et al., 2004). The possibility of bias was due to measurement bias (Diop et al., 2004; Shuey et al., 1999).

###### Optimising interpregnancy intervals

There is no evidence to suggest other risks of bias in three of the included studies (Daniel et al., 2008; Pandey et al., 2016). The risk was unclear with regards to one study (Baqui et al., 2018) due to a possible recall error.

###### Periconceptional folic acid

Two studies appeared free from other risks of bias (Berry et al., 1999; Vergel et al., 1990). Wehby et al. (2012) introduced changes in the methodology during the study and so, it is unclear if it is free from other risks of bias.

###### Periconceptional iron folic acid

Kanani (2000) appears free from other risks of bias.

**Figure 2:**
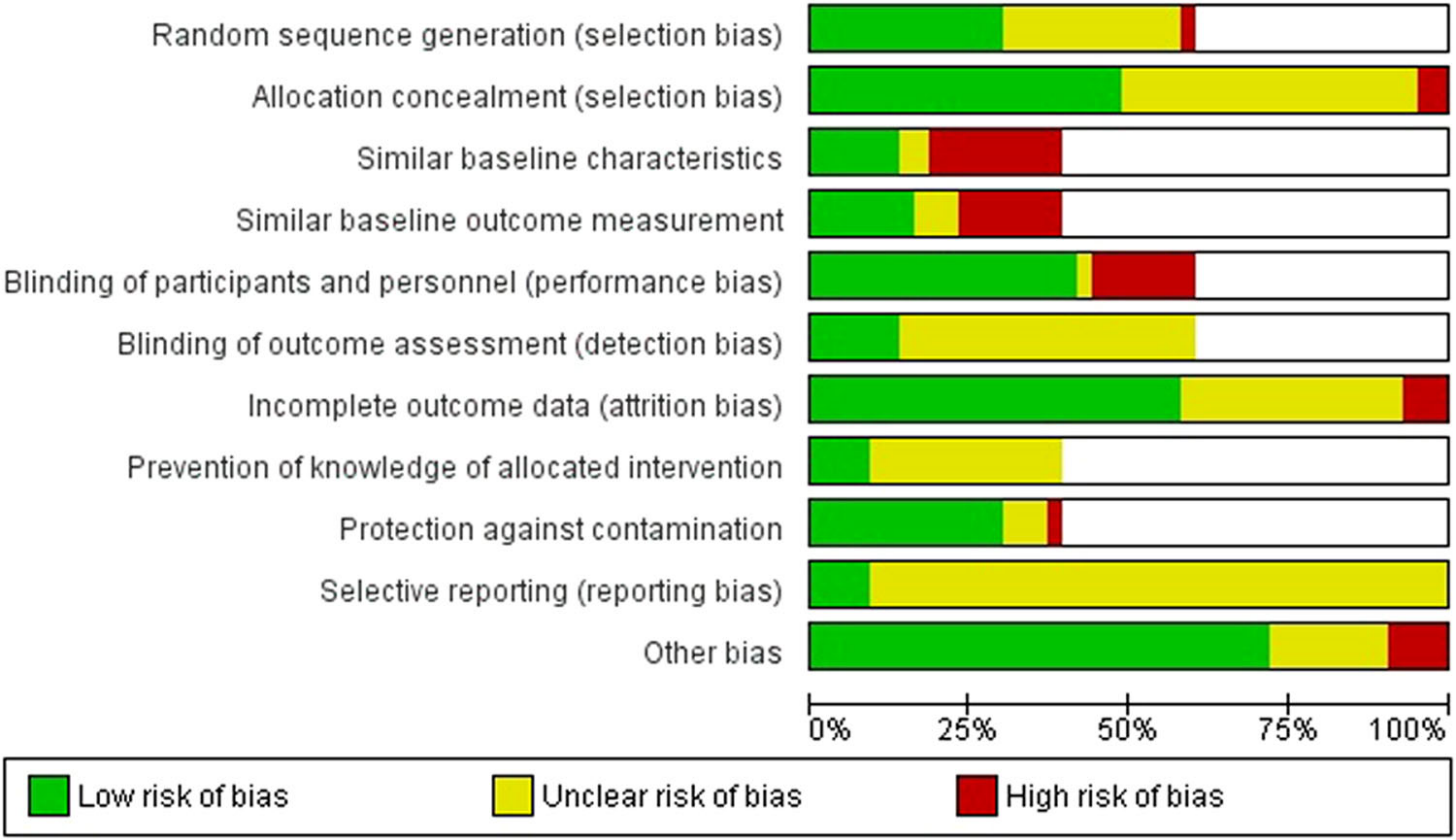
Risk of bias graph: review authors' judgements about each risk of bias item presented as percentages across all included studies

**Figure 3:**
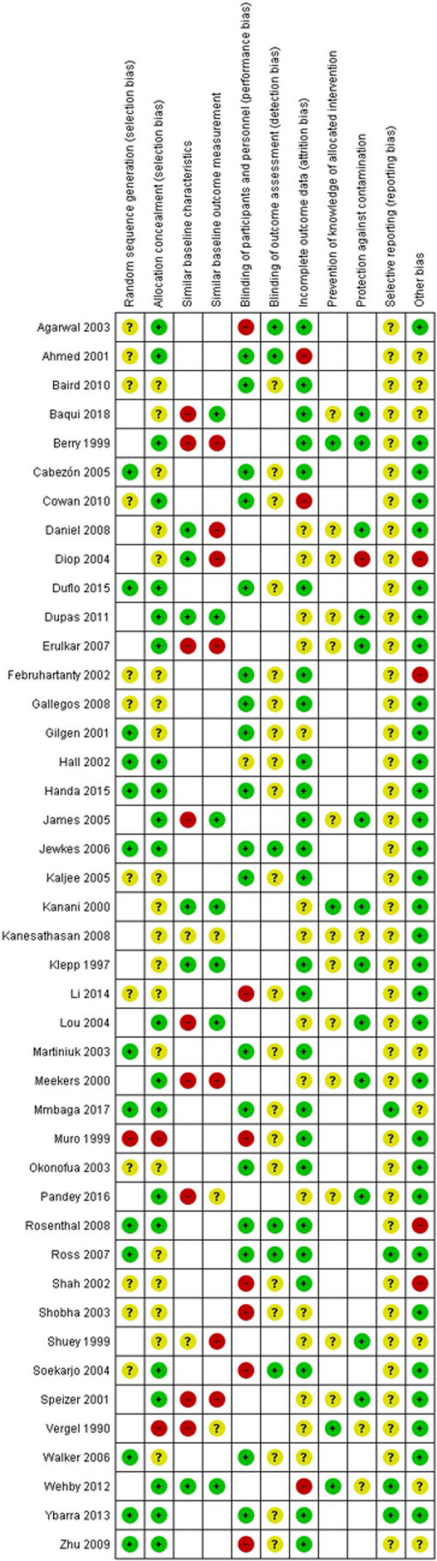
Risk of bias summary: review authors' judgements about each risk of bias item for each included study

Refer to Figures 2 and 3.

#### 5.2.6 Effects of interventions

##### Delaying the age at first pregnancy

1. **Education on sexual health and contraception versus no intervention (24 studies)**

A total of 23 studies compared education on sexual health and contraception with no intervention (Cabezón et al., 2005; Cowan et al., 2010; Daniel et al., 2008; Diop et al., 2004; Duflo et al., 2015; Dupas 2011; Erulkar & Muthengi, 2007; Gallegos et al., 2008; James et al., 2005; Jewkes et al., 2006; Kaljee et al., 2005; Kanesathasan et al., 2008; Klepp et al., 1997; Martiniuk et al., 2003; Meekers, 2000; Mmbaga et al., 2017; Okonofua et al., 2003; Pandey et al., 2016; Ross et al., 2007; Shuey et al., 1999; Speizer et al., 2001; Walker et al., 2006; Ybarra et al., 2013).

##### Primary outcomes

###### Maternal—Unintended pregnancy

The pooled analysis on education on sexual health and contraception showed that intervention may make little or no difference to the reduction in the risk of unintended pregnancy when compared with no education (RR, 0.42; 95% CI, 0.07–3.26; two studies, *n* = 490; random‐effect; *χ* ^2^ *p* = .009; *I* ^2^ = 85%; low certainty of evidence using GRADE assessment) (Analysis 1.1; Table 1).

**Table 1:** Delay in age of first pregnancy—Sexual and reproductive health education versus no intervention

Education compared to no intervention for delaying pregnancy						
Patient or population: delaying at the age at first pregnancy						
Setting: LMICs						
Intervention: Education						
Comparison: no intervention						
Outcomes	Anticipated absolute effects^a^ (95% CI)		Relative effect (95% CI)	No. of participants (studies)	Certainty of the evidence (GRADE)	Comments
	Risk with no intervention	Risk with education				
Unintended pregnancy	Study population		RR 0.42 (0.07–2.36)	490 (2 studies)	⊕⊕⊝⊝ LOW^b^, ^c^	
	122 per 1000	132 per 1000 (41–420)			

*Note*: GRADE Working Group grades of evidence: High certainty: We are very confident that the true effect lies close to that of the estimate of the effect. Moderate certainty: We are moderately confident in the effect estimate: The true effect is likely to be close to the estimate of the effect, but there is a possibility that it is substantially different. Low certainty: Our confidence in the effect estimate is limited: The true effect may be substantially different from the estimate of the effect. Very low certainty: We have very little confidence in the effect estimate: The true effect is likely to be substantially different from the estimate of effect.

Abbreviations: CI, confidence interval; OR, odds ratio; RR, risk ratio.

^a^ The risk in the intervention group (and its 95% CI) is based on the assumed risk in the comparison group and the relative effect of the intervention (and its 95% CI).

^b^ There is high risk of attrition bias due to >20% patients lost to follow up from both intervention and control arms.

^c^ High risk of selection bias.

##### Secondary outcomes

###### Maternal—Reported changes in knowledge and attitudes about the risk of unintended pregnancies (Table 2)

**Table 2:** Outcomes that could not be included in meta‐analysis (delay in the age of first pregnancy)

Name of study	Type of intervention	Outcome			
		Unintended pregnancy	Reported changes in knowledge and attitudes about the risk of unintended pregnancies	Initiation of sexual intercourse	Use of birth control methods
Baird et al. (2010)	Conditional cash transfers				Average condom use for schools girls relative to the post treatment indicator was −0.136 (0.075) and 0.031 (0.201) relative to the round 2 indicator
Cabezón (2005)	Abstinence education	Over 4 years, there were a total of six pregnancies in the intervention group and 35 in the control group. In the 1997 cohort, the pregnancy rates were 3.3% in the intervention and 18.9%, in the control group (RR: 0.176, CI: 0.076–0.408). For the 1998 cohort, the pregnancy rates were 4.4% in the intervention and 22.6%, in the control group (RR 0.195, CI: 0.099–0.384)			
Diop (2004)	Education		This involves one study (Diop 2004). At end line, 185 out of 728 girls knew the risks when an adolescent has sexual intercourse compared to 92 out of 328 girls in the control group. At baseline 179 out of 691 in the intervention group and 83 out of 353 girls in the control group knew this. At end line, 163 out of 212 girls knew the health risks for girls who become pregnant and the child compared to 88 out of 100 girls in the control group. At baseline 144 out of 214 in the intervention group and 80 out of 122 girls in the control group knew this		
Duflo (2015)	Education subsidies and HIV prevention education	While the standalone education subsidy did not decrease unwed pregnancies, it did significantly reduce teenage pregnancies with a 17% difference between the intervention and control groups. Unwed pregnancies were seen to be 1.4% points lower in the standalone HIV education group. This can be extrapolated to give a 30% decrease in 3 years and 18% in 5 years		Age at first sex (if ever had sex): Stand‐alone education subsidy—0.069 (0.095), Stand‐alone HIV education—0.096 (0.092), Joint Programme—0.061 (0.089)	Used a condom at last sexual intercourse (if ever had sex): Stand‐alone education subsidy—0.017 (0.021), Stand‐alone HIV education—0.010 (0.022), Joint Programme‐ −0.021(0.023).
Dupas (2011)	Teacher training/relative risk education/teacher training and relative risk education	The RR information reduced the incidence of childbearing by 1.5 percentage points among treated girls relative to girls in the comparison group (Table 4, column 1). The childbearing rate in the comparison group is 5.4 percent, and thus the RR treatment effect corresponds to a 28 percent decrease in the incidence of childbearing. In contrast, the TT programme had no impact on the incidence of childbearing (row 3)		Table 6—Panel A. Girls Ever had sex (5)— RR information: 0.101 (0.031) TT on HIV/AIDS curriculum: −0.028 (0.023) Observation: 2173 Mean of dependent variable (RR = 0): 0.191	Table 6—Panel A. Girls Used a condom at last sexual intercourse (7)— RR information: 0.118 (0.073) TT on HIV/AIDS curriculum”: 0.012 (0.067) Observations: 307 Mean of dependent variable (RR = 0) 0.360
Gallegos et al. (2008)	Education		There was no significant difference between the experimental and control groups, in the intentions of having sex. However, participants in the experimental group reported higher level of intentions to use condoms [difference of means = 0.15, 95% CI = (0.09, 0.20), *p* < .001] and to use contraceptives [difference of means = 0.16, 95% *IC* = (0.07, 0.24), *p* < .001] in the following 3 months, than those in the control group. Additionally, a linear hierarchical regression model was adjusted to analyse if the effects of the intervention on the outcome variables differed according to the moderating variables, theoretically predictors of the behaviour: age, gender and social compliance. None of these factors interacted with the groups to influence the intentions of having sex or using condoms or contraceptives. This means that the intervention was equally effective for all ages, genders and independent of the tendency to give socially acceptable answers		
James et al. (2005)	Education				42.4% of participants who had reported having sex (in the last 6 months) had consistent condom use. The intervention did not have a significant impact on condom use 6 weeks post intervention
Kaljee et al. (2005)	Education				In regards to intention to engage in sexual intercourse, there was no significant difference between control and intervention groups at either post intervention (*F*1, 459 = .04, *p* = .836) or at 6 months (*F*1, 460 = .29, *p* = .589). However, in regards to the item “if you have sex, would use a condom,” there was an increase in %age of intervention youth who stated that they would “likely” use a condom from baseline (74/240; 30.8%) to postintervention (132/230; 57.4%) and at 6‐month follow‐up (123/228; 53.9%). These increases were significant when compared to control youth at both the post intervention [*F*1, 459 = 12.81, p < .001] and the 6‐month follow–up [*F*1, 459 = 7.82, *p* = .005]
Kanesathasan et al. (2008)	Referrals to micro savings and credit groups and training of health care providers				Current use of a modern contraceptive among married females at end line was 27% in Bihar and 35% I Jharkand
Klepp et al. (1997)	Education		Twelve months after the implementation of the Ngao HIV/AIDS educational programme, students from the intervention schools reported being exposed to AIDS information and discussing HIV/AIDS significantly more frequently than did students from the comparison schools. Students exposed to the programme demonstrated a significant increase in their AIDS‐related knowledge level and reported significantly more positive attitudes toward people with AIDS than did pupils from the comparison schools. Furthermore, 12 months after the implementation of the programme, significant programme effects were seen for subjective norms and intentions with regard to engaging in sexual intercourse. Students attending the intervention schools also reported more restrictive attitudes toward engaging in sexual intercourse than did students from the comparison schools, but this finding was not statistically significant	Finally, we found a nonsignificant trend indicating that fewer students from the intervention schools than from the comparison schools had had their sexual debut during the previous year (7% vs. 17%). This trend was seen for both boys (14% vs 35%) and girls (3% vs 6%)	
Martiniuk et al. (2003)	Education		Difference in knowledge and attitude which was attributable to the intervention was given. Difference was given as difference in change scores, posttest minus pretest, between the two trial arms. For females' knowledge, the crude score was 2.11 with a 95% CI of 0.23–3.99. For females' attitude, the crude score was 0.05 with a 95% CI of −2.60–2.70		
Shuey et al. (1999)	Education		Knowledge that AIDS exists may be a prerequisite to taking decisions to avoid AIDS. In 1994, 270 of 286 (94.4%) of students in the intervention group and 96 of 113 (85.0%) in the control group had heard of AIDS. In 1996, 268 of 279 (96.1%) of students in the intervention group and 114 of 120 (95.0%) in the control group had ever heard of AIDS or slim. The differences between the groups in 1996 in knowing that AIDS exists were not statistically significant and cannot explain the behaviour differences	Using these methods, in the intervention group in 1994, the average age of first intercourse was 11.3 years (range, 6–19; *SD*, 2.9), while in the control group it was 12.4 years (range, 9–18; *SD*, 2.5). In 1996, the mean age of first intercourse in the intervention group was 10.9 years (range, 6–17; *SD*, 2.9), while in the control group it was 12.5 years (range, 7–17; *SD*, 2.7). These changes were not statistically significant	

The outcomes were reported in (Cowan et al., 2010; Daniel et al., 2008; Diop et al., 2004; Martiniuk et al., 2003; Meekers, 2000; Pandey et al., 2016; Ross et al., 2007).

Five studies (Cowan et al., 2010; Daniel et al., 2008; Diop et al., 2004; Meekers, 2000; Ross et al., 2007) reported the impact of education on changing the knowledge regarding pregnancy. Education intervention had a nonsignificant impact on improving the knowledge of pregnancy prevention when compared with no education (RR, 1.02; 95% CI, 0.87–1.21; four studies, *n* = 1433; random‐effect; heterogeneity: *χ* ^2^ *p* = .001; *I* ^2^ = 78%) (Analysis 1.2). The pooled analysis included two RCTs (Cowan et al., 2010; Ross et al., 2007) and three quasi RCTs (Daniel et al., 2008; Diop et al., 2004; Meekers, 2000). On subgroup analysis based on study design, we found education had a significant impact on improving knowledge on pregnancy prevention in RCTs (RR, 1.49; 95% CI, 1.10–2.03; two studies, *n* = 178 random‐effect; heterogeneity: *χ* ^2^ *p* = .6; *I* ^2^ = 0%) whereas it was insignificant in quasi RCTs (RR, 0.94; 95% CI, 0.79–1.12; three studies, *n* = 1355; random‐effect; heterogeneity: *χ* ^2^ *p* = .002; *I* ^2^ = 85%) (Analysis 2.1).

In Martiniuk et al. (2003), the outcome was reported as difference in knowledge and attitude which was attributable to the intervention in change scores, posttest minus pretest, between the two studies arms. For females' knowledge, the crude score was 2.11 with a 95% CI of 0.23–3.99. For females' attitude, the crude score was 0.05 with a 95% CI of −2.60–2.70.

In Pandey et al. (2016), this was reported as the percentage of young women who reported different risks of early child bearing to the mother or child. There was an improvement in knowledge and awareness, with a range of 1.2%–98.4% of young women in the intervention group being aware of specific knowledge related to health outcomes of adolescent pregnancy as noted below.

- Almost 99.6% were aware of risks that a girl may face if she gives birth (at age 15–16) during her adolescence
- 98.4% were aware of risks that a child of an adolescent mother may face
- 92.5% of young unmarried women intended to practice contraception to delay their first pregnancy
- 90.6% were aware about the risk of a weak child
- 86.6% were aware about ill health of mother
- 50.2% were aware about increased possibilities of complications during pregnancy and labour/delivery
- 43.0% were aware about increased risk of maternal mortality
- 39.5% were aware about the risk of infant death
- 38.8% were aware that underdeveloped reproductive organs lead to prolonged or obstructed labour
- 32.0% were aware about the risk of a LBW baby
- 16.0% were aware about the risk of a disabled child
- 10.5% were aware about increased possibility of an underdeveloped child
- 6.1% were aware about miscarriage/still birth
- 4.2% were aware about premature birth/baby
- 1.2% were aware about anaemia in women (Table 2).

##### Maternal—Initiation of sexual intercourse

**Figure 4:**
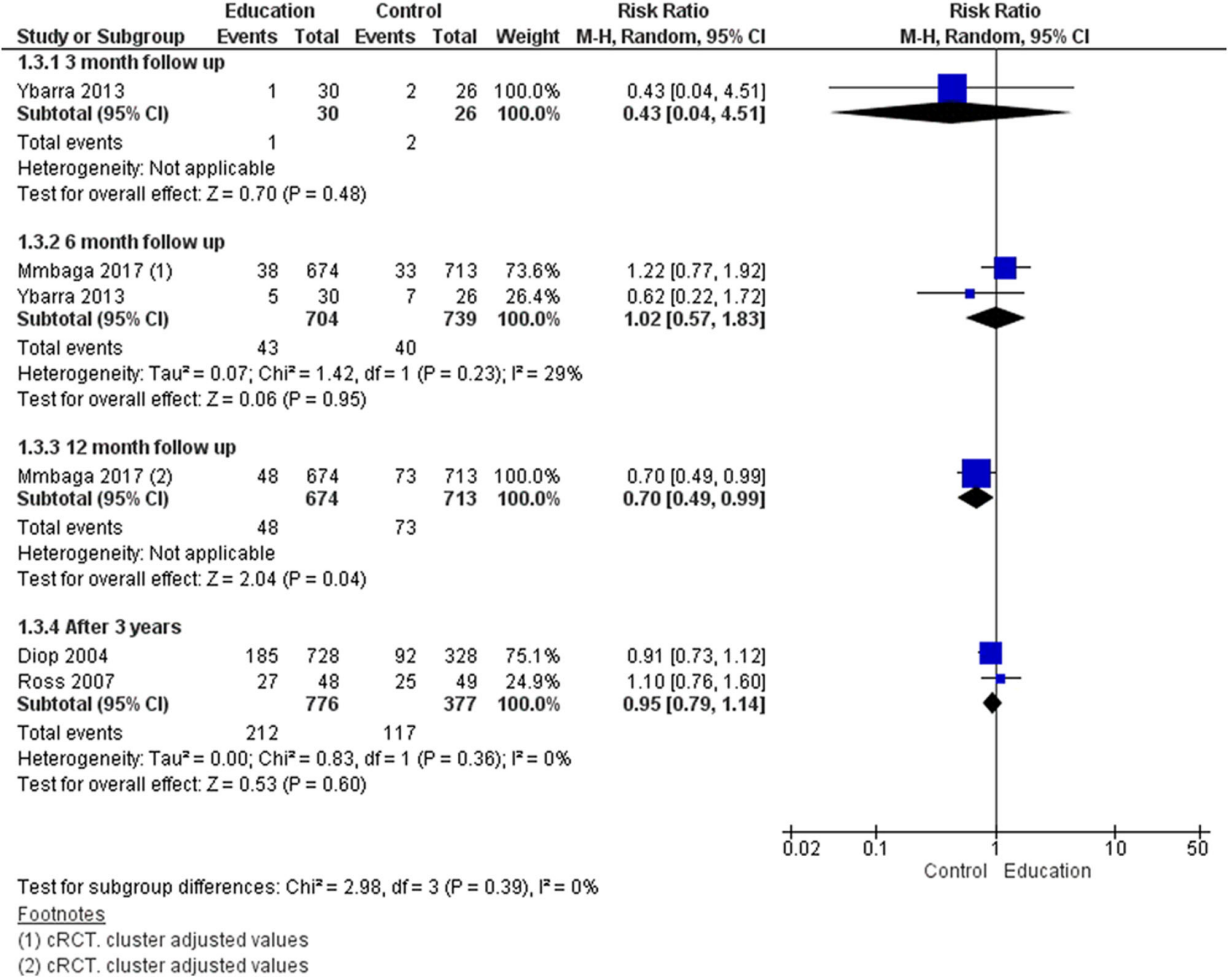
Forest plot of comparison: 1 delay in pregnancy—education versus no intervention, outcome: 1.3 initiation of sexual activity

This outcome was reported in Diop et al. (2004), Mmbaga et al. (2017), Ross et al. (2007), and Ybarra et al. (2013). It was pooled based on the time of follow up (3, 6, and 12 months and at 3 years). Education on reducing sexual risks did not have a significant impact on age of sexual debut at the 3 month (RR, 0.43; 95% CI, 0.04–4.51; one study, *n* = 56), at 6 month (RR, 1.02; 95% CI, 0.57–1.83; two studies, *n* = 1443; random‐effect; heterogeneity: *χ* ^2^ *p* = .23, *I* ^2^ = 29%), or at 3 years follow up (RR, 0.95; 95% CI, 0.79–1.14; two studies, *n* = 1153; random‐effect; heterogeneity: *χ* ^2^ *p* = .36, *I* ^2^ = 0%). There was a significant decrease in the age of sexual initiation at the 12 month follow up (RR, 0.70; 95% CI, 0.49–0.99; one study, *n* = 1387) (Analysis 1.3; Figure 4).

When considering subgroups based on setting and study design, separate syntheses were conducted based on the time of follow up (separated into 3‐, 6‐ and 12‐month follow‐ups).

For the 3 month follow up outcome, there was one study (Ybarra et al., 2013) which was an RCT which took place in a school setting. For the 6 month follow up outcome, there were two studies (Mmbaga et al., 2017; Ybarra et al., 2013). The first study was a cRCT and so the effect of clustering was controlled. All of the studies were RCTs so there was a single sub group for study type. For the 12 month follow up outcome, there was one study (Mmbaga et al., 2017). It was an RCT which was a cluster randomised and so clustering was taken into account and controlled. lt took place in combined school and clinic settings. For 3 years follow up, there was one study (Ross et al., 2007) and one quasi RCT (Diop et al., 2004).

One study took place in a school setting (Ybarra et al., 2013), while one occurred in a combined school and clinic setting (Mmbaga et al., 2017). The effect of intervention was not significant when compared to no education when it took place in schools (RR, 0.62; 95% CI, 0.22–1.72; one study, *n* = 56) or in a combination of school and clinics (RR, 1.22; 95% CI, 0.77–1.92; one study, *n* = 1387 Table 3).

**Table 3:** Matrix—Delay in the age of first pregnancy

Study	Study design	Intervention	Duration	Dosage	Moderators of delivery	Setting	Timing of intervention
Cabezón (2005)	Cluster RCT	Sex education	1 year (study for 4 years)		Teachers	Schools	Preconception
Cowan (2010)	Cluster RCT	Regai Dzive Shiri intervention (an in‐school teaching programme, training of nurses and raising awareness and improving communication in the community with regards to HIV prevention)	2003–2007 (this time period includes both the early and delayed implementation)		Research staff, nurses, school leavers	Community, schools and clinics	Preconception
Diop (2004)	Quasi‐experimental design based in three urban communities in northern Senegal. pre‐ and posttest control group design	Sensitisation on adolescent reproductive health for community and religious leaders, reaching parents through women's groups, and education sessions led by peer educators using a life skills curriculum providers and peer educators were trained to offer youth‐friendly services trained teachers and peer educators to provide reproductive health information through a reproductive health curriculum tailored to in‐school youth	3 years		Providers, peer educators, teachers	Community‐based intervention, school‐based and clinic based. The communities of Louga and Saint‐Louis served as intervention sites, while Diourbel served as a control site. Both intervention sites offered the community‐ and clinic‐based interventions; and Saint‐Louis also introduced the school‐based intervention	Preconception
Duflo (2015)	Randomised evaluation—RCT	The first programme reduced the cost of education by providing free school uniforms. The second programme trained teachers on how to deliver the national HIV/AIDS prevention curriculum to upper primary school students. We also evaluate a small add‐on component to the government‐run teacher training designed to foster the discussion of condoms, in order to check whether an explicit discussion of condoms in a curriculum otherwise focused on abstinence and fidelity could affect behaviour	Education subsidy—2 uniforms one each year in 2003 and 2004. 2 years of HIV education (please recheck)		Teachers	School	Preconception
Dupas (2011)	Randomised experiment	Four groups of schools: (1) teacher training programme and relative risk information, (2) only teacher training, (3) only relative risk information, and (4) neither programmes	Rr group had a 40 min talk with 10 min video		Teachers, facilitators, project officers	School	Preconception
Erulkar (2007)	Programme evaluative reports	(1) Social mobilisation and group formation by adult female mentors, (2) participation in non formal education and livelihoods training for out of school girls, or support to remain in school, and (3) “community conversations”	2 years		Mentors, facilitators	Community	
Handa et al. (2015)	Cluster RCT	Cash transfer to take care of orphan and vulnerable children in the household	2007–2011 (4 years)		Location OVC committees	Community	
James et al. (2005)	Pre‐ and posttest follow up design	systematically developed photo‐novella (Laduma) on knowledge, attitudes, communication and behavioural intentions with respect to sexually transmitted infections	One reading (about 1 h)		Laduma was given to read. The questionnaire was individually filled in by learners and was supervised by a fieldworker and researcher during a normal class lesson	School	Preconception
Jewkes et al. (2006)	Cluster RCT	Behavioural intervention	17 sessions (50 h) over a period of 3–12 weeks control arm communities attended a single session of about 3 h on HIV and safer sex		Trained facilitators	Community	
Kanesathasan et al. (2008)	Quasi‐experimental study design	(1) Mass or generalised: limited engagement with youth, (2) targeted or individualised: youth groups (provided young people with health information on a range of topics, including adolescence, gender and sexuality, fertility awareness, contraception, HIV and AIDS, safe motherhood, and reproductive health services), peer education (volunteer peer educators—married and unmarried males and females—to provide information, counselling, support and referrals to their peers through youth groups and individual sessions.), livelihoods (income generating opportunities and skills)	2005–2007		Peer educators, youth depot holders and health service providers	Community, institutional	
Klepp et al. (1997)	RCT	Education programme designed to reduce children's risk to HIV and improve tolerance of and care for people with AIDS	A 2‐ to 3‐month period, averaging about 20 school hours of class		Teachers and local health workers	School	Preconception
Lou et al. (2004)	Quasi experimental study	The intervention intended to build awareness and offer counselling and services related to sexuality and reproduction among unmarried youths, in addition to the routine programme activities, which were exclusively provided in the control site	20 months		Research staff, professional educator, health counsellor	Community	Preconception
Meekers (2000)	Quasi experimental	Targeted social marketing programme on reproductive health beliefs and behaviour	May 1997 to April 1997 (12 months)		Trained adolescents	Community	Preconception
Mmbaga et al. (2017)	Randomised controlled trial	The PREPARE project. It was a curriculum based intervention targeting students in grade 5–6 (aged 12–14 years). Topics covered included “self‐awareness,” “my sexuality,” “relationships,” “what influences my sexuality,” “risk taking sexual behaviours and consequences,” “self‐ protection,” “decision making skills” and “puberty”	9 weeks. The in‐school portion consisted of nine lessons were. Six were taught by teachers (total 11 h) and consisted of 16 sessions lasting 60–90 min each. Three were peer led (total 8 h) that consisted of weekly lessons lasting 60–90 min. Total time for the in‐school portion was 19 h. The study also included visits to health facilities but specific timings are not mentioned		Trained teachers, peer‐educators and health care providers	Public primary schools and youth friendly health service clinics	?
Okonofua et al. (2003)	Randomised controlled trial	It consisted of community participation, peer education, public lectures, health clubs in the schools, and training of STD treatment providers, including those with no formal training. It had three main components: Establishment of a reproductive health club at each school that organised STD prevention and treatment activities including a series of health awareness campaigns led by health professionals, distribution of educational material as well as organising debates, dramas, essay writing, symposia and film screening. Training some club members to become peer educators, over a period of 4 weeks, who then had one on one or group sessions with students, distributed relevant material and referred students to health care providers. Training local adolescent friendly health providers about the diagnosis and treatment of STDs	11 months. The intervention began in early September 1997 and the post intervention survey took place in July 1998		Researchers, health care providers, reproductive health club members and peer educators	Secondary schools in Benin city. Classes 4–5 were included	?
Pandey et al. (2016)	Quasi‐ experimental programme study	PRACHAR's adolescent training programme, which imparted knowledge on a range of issues related to reproduction, family planning, and responsible decision‐making in the area of reproductive health	3 days (5 h/day)		Two pairs of female trainers for participants that were girls and two teams of 1 male and 1 female trainer for participants who were boys	Rural communities	
Speizer et al. (2001)	Quasi‐experimental study	Peer‐based adolescent reproductive health education	18 months		Peer‐educators. They were trained by health professional with experience in health education and reproductive health	Communities	
Walker et al. (2006)	Cluster RCT	(1) HIV education, skills‐ building, cultural values, contraceptive promotion (condoms), (2) HIV education, skills‐ building, cultural values plus contraceptive education (EC plus condoms and their access)	Students at intervention schools received a 30 h course (over 15 weeks) on HIV prevention and life skills, designed in accordance with guidelines of the joint United Nations programme on HIV/AIDS. Two extra hours of education on emergency contraception were given to students in the condom promotion with contraception arm		Teachers	School	Preconception
Ybarra et al. (2013)	Parallel group RCT	Online health sexuality programme	5 h with half group receiving booster at 4 months		RAs (research associates?)	School	Preconception
Baird et al. (2010)	Cluster RCT	Cash transfer (unconditional and conditional)	2 year monthly cash transfer		NGOs	School/community	Preconception
Gallegos et al. (2008) (table in Mexican)	RCT	Small group discussions, use of videos and interactive exercises (a) reduction of HIV/AIDS risk and (b) health promotion	6 h total (2 sessions one in a week)		Facilitators	Schools	Preconception
Ross et al. (2007)	Community randomised trial	Community activities; teacher‐led, peer‐assisted sexual health education in years 5–7 of primary school; training and supervision of health workers to provide “youth‐friendly” sexual health services; and peer condom social marketing	In school programme12 40 min session/year Health worker trained to 1 week Competition—twice yearly (total 3 years)		Teachers, health workers, peer‐elected youth for condom distribution, local youth groups	Community/school	Preconception
Daniel (2008)	Quasi‐ experimental programme study	Educational workshops and behaviour change communication. Elements included were social environment building, providing information on reproductive health and services, and improving access to reproductive health services	Implementation began in July 2002 in Nalanda, in October 2002 in Nawada and in April 2003 in Patna. The first phase of PRACHAR ended in 2005–2006. Phase II was being implemented when this paper was written and was supposed to continue through July 2009		Change agents and training officers	Rural communities	Preconceptional
Martiniuk et al. (2003)	Cluster RCT	Responsible sexuality education programme; which is a 3‐h scripted responsible sexuality education intervention which provides a framework for adolescents for decision making in relationships and provides unbiased information about sex and sexuality	3 h			High schools	
Shuey et al. (1999)	Quasi randomised study	School health education programme in primary schools. This programme aimed to improve access to information and other resources for healthy sexual behaviour decision making, improve adolescent to adolescent interaction regarding information and decision making (relating to AIDS, sexuality and health), and improving the quality of the existing district educational system in the implementation of the school health curriculum and in counselling/advice giving to students. This intervention consisted of nine activities involving the community, parents, local leaders, teachers, students and school health clubs			One full time health educator, staff already present on the district education and health teams, Senior men and women tutors are	Rural and town sites. School setting	
Kaljee et al. (2005)	RCT	The Vietnamese Focus on Kids programme includes eight sessions and two sessions for community project development and delivery	The programme took place once a week for 10 consecutive weeks. Each session was approximately 2 h long.		The facilitators included teachers, youth leaders, and commune centre health care providers	Local schools. Rural community	

##### Maternal—Use of birth control methods

**Figure 5:**
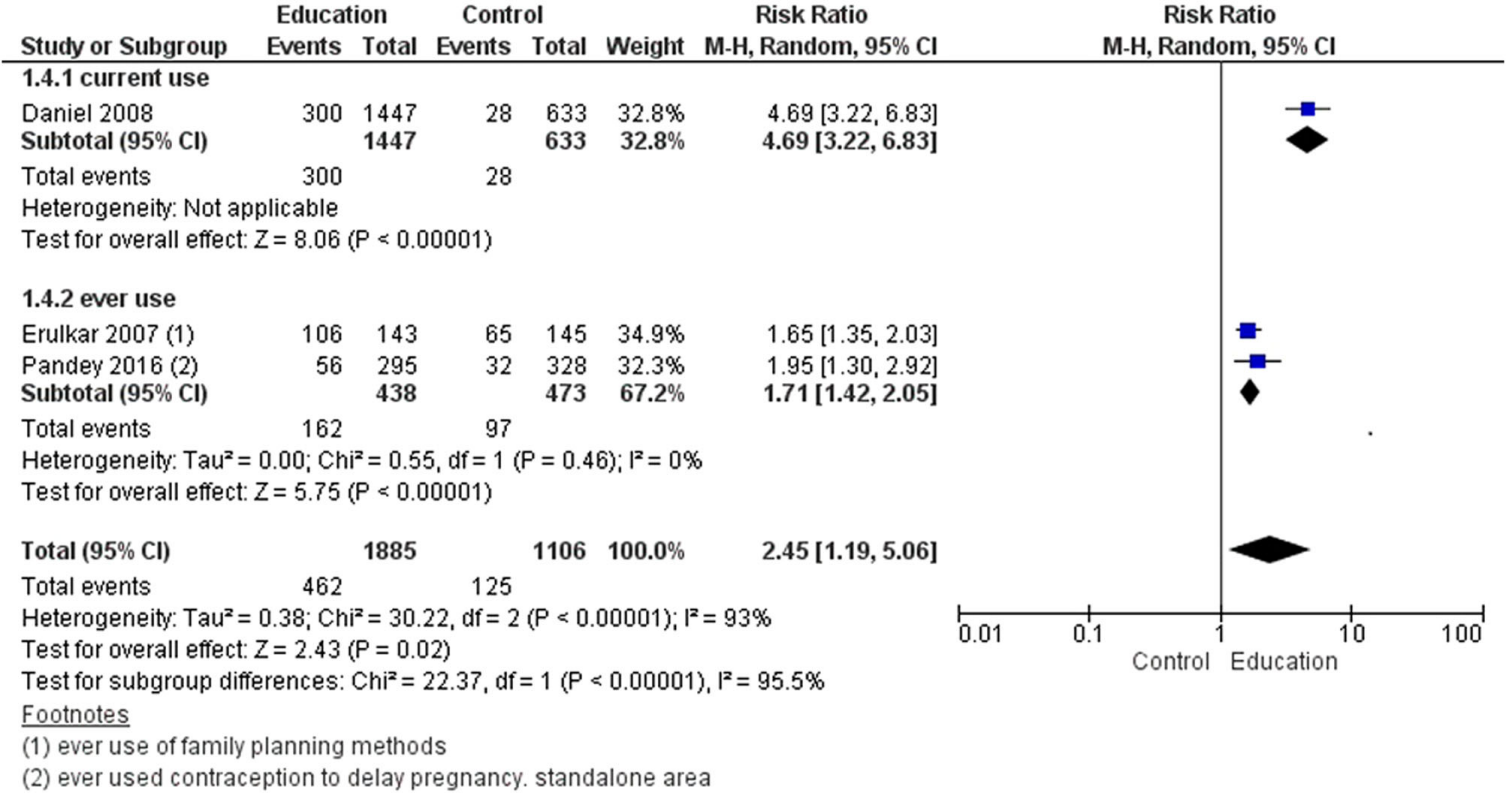
Forest plot of comparison: 1 delay in pregnancy—education versus no intervention, outcome: 1.4 use of any contraception

Use of contraception: The outcome was reported in three studies (Daniel et al., 2008; Erulkar & Muthengi, 2007; Pandey et al., 2016). One quasi experimental study in community settings (Daniel et al., 2008) described current contraception use and two studies (Erulkar & Muthengi, 2007; Pandey et al., 2016) described ever use of contraception. Education on contraception had a significant impact on the usage of contraception when compared with no education (RR, 2.45; 95% CI, 1.19–5.06; three studies, *n* = 2991; random‐effect; heterogeneity: *χ* ^2^ *p* < .00001; *I* ^2^ = 93%). Education on contraception had a significant impact on the current use of contraception when compared with no education (RR, 4.69; 95% CI, 3.22–6.83; one study, *n* = 2080) as well as on ever use of contraception (RR, 1.71; 95% CI, 1.42–2.05; two studies, *n *= 911; random‐effect, Heterogeneity: *χ* ^2^ *p* = .46; *I* ^2^ = 0%) (Analysis 1.4; Figure 5).

For ever contraception use, the subgroups according to setting were school (Erulkar & Muthengi, 2007) and community (Pandey et al., 2016). Education on contraception had a significant impact on ever contraception use when compared with no education for school (RR, 1.65; 95% CI, 1.35–2.03; one study, *n* = 288), but insignificant for community (RR, 1.95; 95% CI, 1.30–23.92; one study; *n* = 623) settings. However, the findings are coming from a single study.

**Figure 6:**
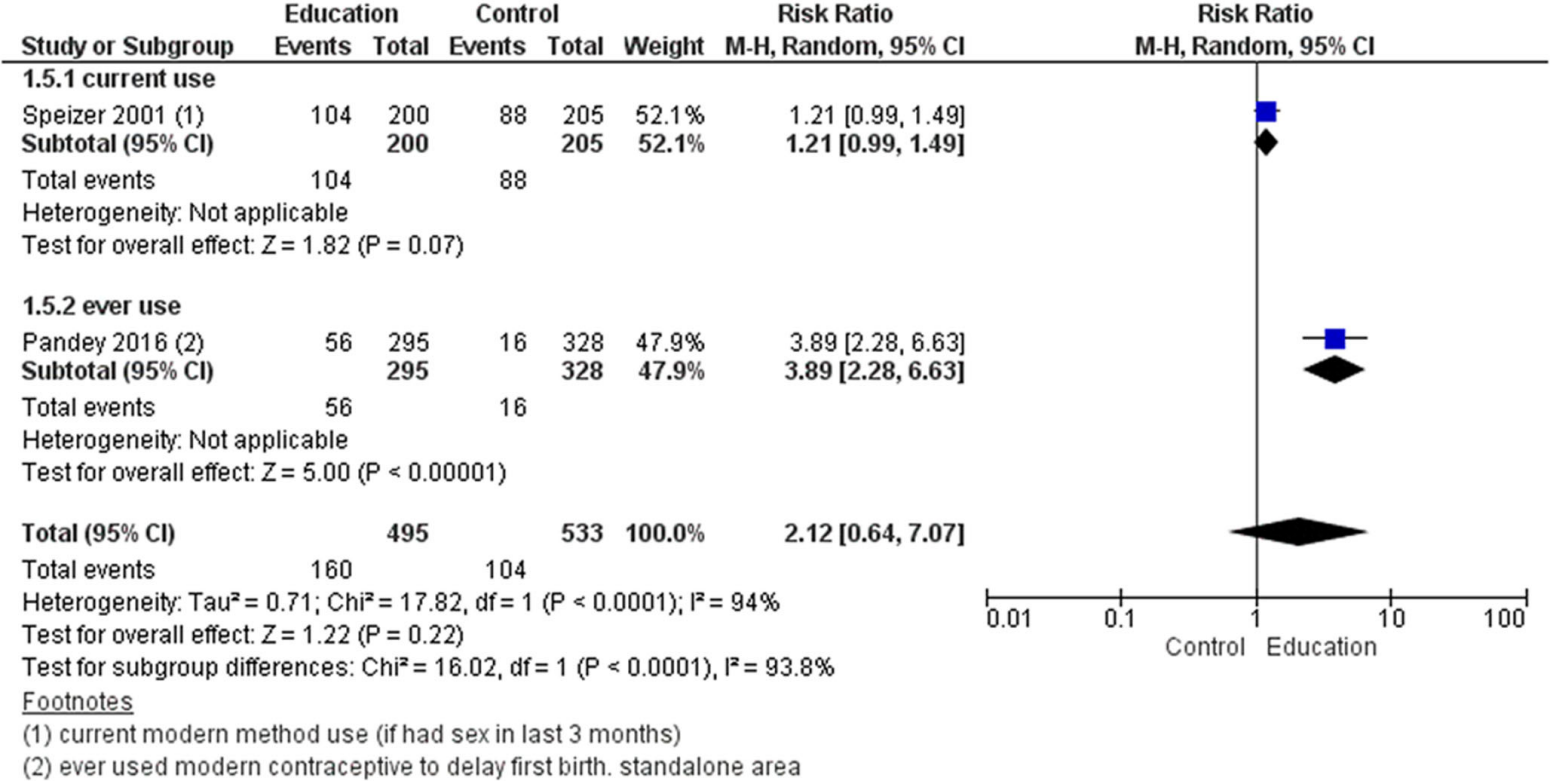
Forest plot of comparison: 1 delay in pregnancy—education versus no intervention, outcome: 1.5 use of a modern method

Use of a modern method: Two quasi studies from community settings reported this outcome with a total of 2466 women (Pandey et al., 2016; Speizer et al., 2001). Speizer et al. (2001) reported current use of modern birth control methods while Pandey et al. (2016) reported ever used. Education on contraception did not have a significant impact on the usage of modern methods of contraception when compared with no education (RR, 2.12; 95% CI, 0.64–7.07; two studies, *n* = 1028; random‐effect, heterogeneity: *χ* ^2^ *p* < .0001; *I* ^2^ = 94%) (Analysis 1.5; Figure 6).

Use of a traditional method: One quasi‐experimental study that took place in a community setting reported this outcome (Pandey et al., 2016), with a total of 2061 women. Education on contraception did not have a significant impact on the usage of traditional methods of contraception when compared with no education (RR, 1.70; 95% CI, 0.94–3.07, one study; *n* = 623) (Analysis 1.6).

**Figure 7:**
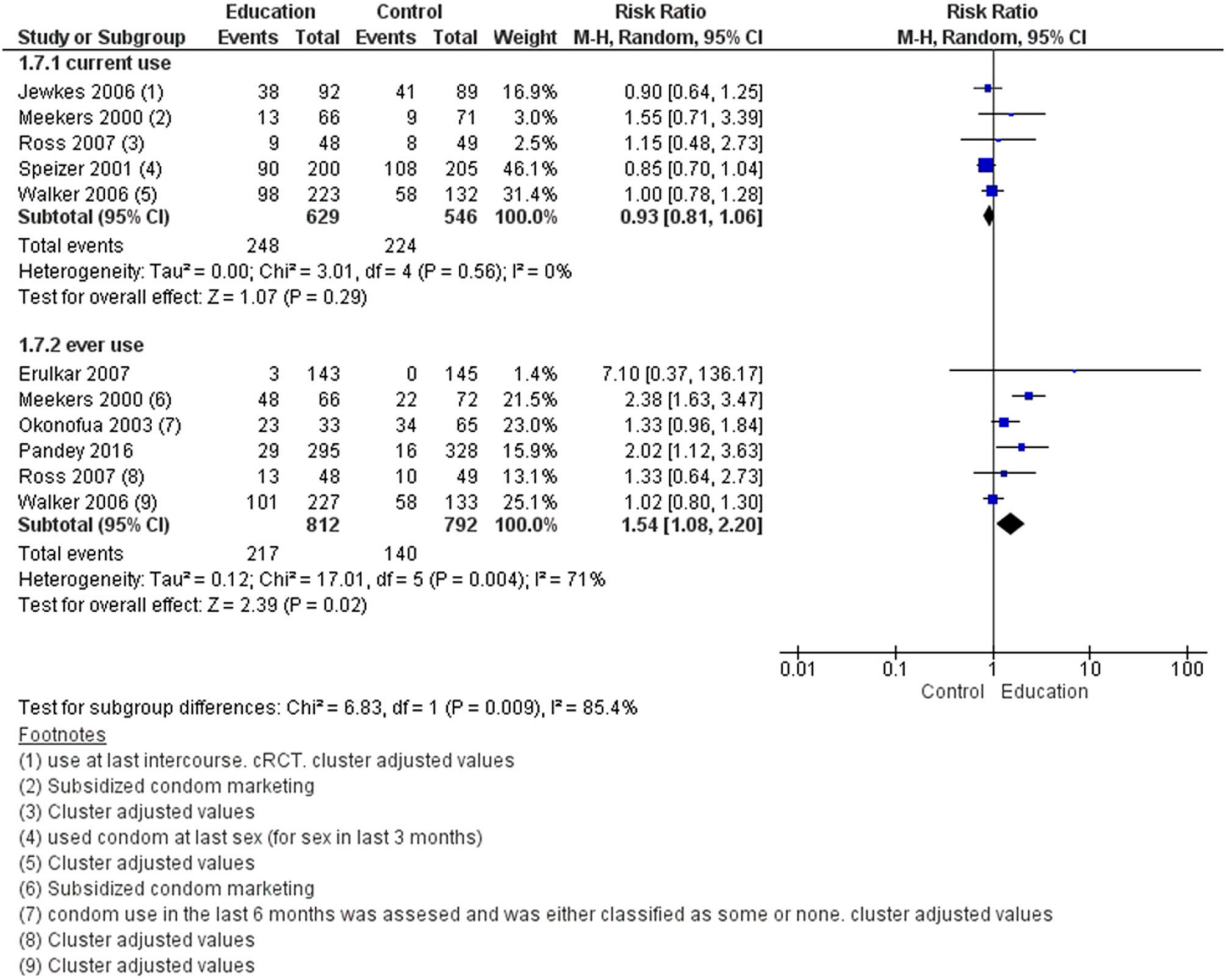
Forest plot of comparison: 1 delay in age of first pregnancy—education versus no intervention, outcome: 1.7 use of condoms

Use of condoms: Eight studies (Erulkar & Muthengi, 2007; Jewkes et al., 2006; Meekers, 2000; Okonofua et al., 2003; Pandey et al., 2016; Ross et al., 2007; Speizer et al., 2001; Walker et al., 2006) reported this outcome. Of these eight studies, five reported reported current use (Jewkes et al., 2006; Meekers, 2000; Ross et al., 2007; Speizer et al., 2001; Walker et al., 2006) and six reported ever use of condom (Erulkar & Muthengi, 2007; Meekers, 2000; Okonofua et al., 2003; Ross et al., 2007; Speizer et al., 2001; Walker et al., 2006). Education on contraception did not have a significant impact on current condom use when compared with no education (RR, 0.93; 95% CI, 0.81–1.06; five studies, *n* = 1175; random‐effect, heterogeneity: *χ* ^2^ *p* = .56; *I* ^2^ = 0%). However, education on contraception significantly improved ever use of condom (RR, 1.54; 95% CI, 1.08–2.20; six studies, *n* = 1604; random‐effect, heterogeneity: *χ* ^2^ *p* = .004; *I* ^2^ = 71%) (Analysis 1.7; Figure 7).

Use of pills: Use of contraceptive pills was reported in one study of this comparison (Erulkar & Muthengi, 2007), with a total of 926 females. Education on contraception had a significant impact on the use of pills when compared with no education (RR, 1.34; 95% CI, 0.89–2.01; one study, *n* = 288) (Analysis 1.8).

Use of depot/injectable methods: This outcome was reported in one study of this comparison (Erulkar & Muthengi, 2007), with a total of 926 females. Education on contraception had a significant impact on the use of depot/injectable methods when compared with no education (RR, 1.58; 95% CI, 1.26–1.98; one study, *n* = 288) (Analysis 1.9).

##### Maternal—Abortion

One study (Cowan et al., 2010) reported the risk of abortion. Education on reproductive health did not have a significant impact on the risk of abortion when compared with no education (RR, 1.11; 95% CI, −0.07–17.06, one study, *n* = 80). The study type was RCT and the setting was a combination of community, schools and clinics (Analysis 1.10).

2. **Education on sexual health + provision of contraceptives versus no intervention (one study)**

This comparison involved one study (Lou et al., 2004), with a total 470 women and primary outcomes were not reported.

##### Secondary outcomes

###### Maternal—Use of birth control methods

Use of any contraception: The outcome was reported as regular and as ever use. SRH education and provision of contraception had a significant impact on use of regular contraception (RR, 1.90; 95% CI, 1.71–2.10; one study, *n* = 954; random‐effect) and ever use of contraception (RR, 1.17; 95% CI, 1.12–1.22; one study, *n* = 954) when compared to no intervention (Analysis 3.1).

Condom use: Education and provision of contraception had a significant impact on condom use when compared to no intervention (RR, 1.14; 95% CI, 1.09–1.19; one study, *n* = 954; random‐effect) (Analysis 3.2).

3. Conditional cash transfer versus no intervention (two studies)

This involved two studies (Baird et al., 2010; Handa et al., 2015). It was not included in the meta‐analysis as the results are reported as regressions.

##### Primary outcomes

Unintended pregnancy: Handa et al. (2015) reported that cash transfers to vulnerable adolescent girls reduced the likelihood of pregnancy by 5%. However there was no significant impact on likelihood of early marriage.

##### Secondary outcomes

Maternal—Use of birth control methods: Average condom use for schools girls relative to the post treatment indicator was −0.136 (0.075) (Baird et al., 2010) (Table 2).

#### 5.2.7 Optimising interpregnancy intervals

1. Sexual and reproductive health education versus no intervention (two studies) (Table 4).

**Table 4:** Outcomes that could not be included in the meta‐analysis (optimising interpregnancy interval)

Name of study	Type of intervention	Outcome			
		Unintended pregnancy	Reported changes in knowledge and attitudes about the risk of unintended pregnancies	Initiation of sexual intercourse	Use of birth control methods
Daniel (2008)	Education		The reported change in the percentage of married women aged 15–24 that agreed that early childbearing is injurious to a mother's health increased from 17% to 74% in the intervention group and 12%–65% in the control group. The reported change in the percentage of married women aged 15–24 that agreed that contraceptive use is safe and necessary for delaying first birth increased from 38% to 80% in the intervention group and 36%–72% in the control group		In Pandey et al. (6), 29.7% of the young married women (who had at least had one birth) in standalone areas reported the use of any modern contraceptive method, compared to 18.9% in control areas. 19.8% of the young married women (who had at least had one birth) in standalone areas reported the use of any modern spacing method, compared to 9.2% in control areas
Pandey et al. (2016)	Education and counselling, referrals, family members' education and improvement of contraceptive services		One study (Pandey et al. 2016) was included. In Pandey et al. (2016), 34.5% of the young married women (who had at least had one birth) in comprehensive areas reported the use of any modern contraceptive method, compared to 18.9% in control areas. 20.5% of the young married women (who had at least had one birth) in comprehensive areas reported the use of any modern spacing method, compared to 9.2% in control areas		
Baqui (2018)	Family planning integrated to maternal newborn care package				Compared to the control arm, women in the intervention arm had a 19% lower risk of short birth interval (adjusted RR) = 0.81, 95% CI = 0.69–0.95) and 21% lower risk of preterm birth (adjusted RR = 0.79; 95% CI = 0.63–0.99)

Abbreviation: CI, confidence interval.

This involved two quasi RCTs (Daniel et al., 2008; Pandey et al., 2016) and no primary outcomes were reported.

##### Secondary outcomes

###### Maternal—Use of birth control methods

Two studies (Daniel et al., 2008; Pandey et al., 2016), with a total of 6136 women reported use of modern methods and any birth control method.

###### Maternal—Reported changes in knowledge and attitudes about the risk of unintended pregnancies

In Daniel et al. (2008), the reported change in the percentage of married women aged 15–24 that agreed that early childbearing is injurious to a mother's health increased from 17% to 74% in the intervention group (*N* = 1,447) and 12%–65% in the control group (*N* = 633). The reported change in the percentage of married women aged 15–24 that agreed that contraceptive use is safe and necessary for delaying first birth increased from 38 to 80% in the intervention group and 36%–72% in the control group (Table 4).

Use of any contraception: This involved two studies (Daniel et al., 2008; Pandey et al., 2016). Education on sexual health with provision of contraceptive and involvement of male partner had a significant impact on the use of contraception compared with no intervention (RR, 1.83; 95% CI, 1.26–2.66; one study, *n* = 338), however, education alone did not have a significant impact on improving the use of contraception when compared with no education (RR, 2.72; 95% CI, 0.88–8.40; two studies, *n* = 2385; random‐effect, heterogeneity: *χ* ^2^ *p* < .0001; *I* ^2^ = 94%) (Analysis 4.1). Both studies reported current use of contraception.

Use of a modern method: One study (Pandey et al., 2016), involving a total of 2061 women reported the use of modern methods of contraception. Education on sexual health with provision of contraceptive and involvement of male partner had a significant impact on the use of modern methods of contraception compared with no intervention (RR, 2.25, 95% CI, 1.29–3.93; one study, *n* = 338; random‐effect). However, education alone also showed a significant impact on use of modern methods of contraception when compared with no education (RR, 2.45; 95% CI, 1.42–4.24; one study, *n* = 338; random‐effect) (Analysis 4.2).

Sub groups according to study setting and type were not made for this as both of the studies (Daniel et al., 2008, Pandey et al., 2016) under this comparison were quasi experimental studies that took place in community settings. None of the included studies reported on unintended pregnancy, initiation of sexual intercourse, abortion and preterm birth (Table 5).

2. **Education on sexual health + provision of contraception + involvement of male partner versus education on sexual health and contraception only (one study).**

**Table 5:** Matrix—Optimising interpregnancy interval

Study	Study design	Intervention	Duration	Dosage	Moderators of delivery	Setting
Baqui (2018)	Cluster quasi study	integrated post‐partum family planning and maternal and newborn health (PPFP‐MNH) interventions	During the enrolment pregnancy (baseline), hereafter referred to as “index” pregnancy and during the postpartum period through 36 months postpartum		Trained community health workers	Community level unions (unions are the lowest administrative units in Bangladesh with an average population of about 25,000 and a health centre known as Health and Family Welfare Center—H&FWC) in two subdistricts in the Sylhet district
Daniel (2008)	Quasi‐ experimental programme study	Educational workshops and behaviour change communication. Elements included were social environment building, providing information on reproductive health and services, and improving access to reproductive health services	Implementation began in July 2002 in Nalanda, in October 2002 in Nawada and in April 2003 in Patna. The first phase of PRACHAR ended in 2005–2006. Phase II was being implemented when this paper was written and was supposed to continue through July 2009		Change agents and training officers	Rural communities
Rosenberg (2015a)	Retrospective cohort study	Child support grant	Variable. To be eligible in 1998, caregivers needed to care for a child under 7. The programme expanded in April 2003 to include children up to age nine; in April 2004 up to age 11; in April 2005 up to age 14; in April 2009 up to age 15; and in April 2010 up to age 18	100–330 Rand (US$8 to US$27) per month per child	Government of South Africa	Rural community
Zhu et al. (2009)	Cluster randomised trial	Post‐abortion family planning service package. Package A included provision of limited information and referral to existing services, and the other, more comprehensive, package B consisted–in addition to the above simple package–of individual counselling, free provision of contraceptive materials, and involvement of the male partner	May to November 2006		Researchers	Abortion clinics (department of gynaecology in hospitals)
Pandey et al. (2016)	Longitudinal study	PRACHAR's adolescent training programme, which imparted knowledge on a range of issues related to reproduction, family planning, and responsible decision‐making in the area of reproductive health	3 days (5 h a day)		Two pairs of female trainers for participants that were girls and 2 teams of 1 male and 1 female trainer for participants who were boys	Rural communities in selected villages of the Gaya district. There were 2 types of settings, standalone (where no other PRACHAR Phase III activities were conducted) and comprehensive (where other PRACHAR

One study (Zhu et al., 2009) with a total of 2336 women was included. This study compared education on sexual health and provision of contraceptive, along with involvement of male partner with education only.

##### Primary outcomes

###### Maternal—Unintended pregnancy

Education on sexual health and provision of contraceptive along with involvement of male partner probably makes little or no difference to the risk of unintended pregnancies when compared to education on sexual health only (RR, 0.32; 95% CI, 0.01–7.45; one study, *n* = 45; moderate certainty of evidence using GRADE assessments) (Analysis 5.1; Table 6).

**Table 6:** Optimising interpregnancy interval—Education on sexual health and contraception + provision of contraception + involvement of male partner versus education on sexual health and contraceptionalone

Education + referral services + training of service providers + counselling + provision of contraception + involvement of male partner compared to education + referral services in pregnancy						
Patient or population: pregnancy						
Setting: LMICs						
Intervention: education + referral services + training of service providers + counselling + provision of contraception + involvement of male partner						
Comparison: education + referral services						
Outcomes	Anticipated absolute effects^a^ (95% CI)		Relative effect (95% CI)	No. of participants (studies)	Certainty of the evidence (GRADE)	Comments
	Risk with education + referral services	Risk with education + referral services + training of service providers + counselling + provision of contraception + involvement of male partner				
unintended pregnancies	Study population		RR 0.32 (0.01 to 7.45)	45 (1 RCT)	⊕⊕⊕⊝ MODERATE^b^, ^c^	
	45 per 1000	15 per 1000 (0 to 339)			

*Note*: GRADE Working Group grades of evidence. High certainty: We are very confident that the true effect lies close to that of the estimate of the effect. Moderate certainty: We are moderately confident in the effect estimate: The true effect is likely to be close to the estimate of the effect, but there is a possibility that it is substantially different. Low certainty: Our confidence in the effect estimate is limited: The true effect may be substantially different from the estimate of the effect. Very low certainty: We have very little confidence in the effect estimate: The true effect is likely to be substantially different from the estimate of effect.

Abbreviations: CI, confidence interval; OR, odds ratio; RR, risk ratio.

^a^ The risk in the intervention group (and its 95% confidence interval) is based on the assumed risk in the comparison group and the relative effect of the intervention (and its 95% CI).

^b^ Heterogeneity not applicable as there is only one study under this comparison.

^c^ Total number of events is <300.

##### Secondary outcomes

###### Maternal—Use of birth control methods

The use of contraceptive methods was given in a following different ways:

Use of any contraceptive method: Education on and provision of contraceptive along with involvement of male partner had no significant impact on the use of contraception when compared to education alone (RR, 1.05; 95% CI, 0.91–1.21; one study, *n* = 39) (Analysis 5.2).

Use of condoms, oral contraceptives (OCs), intrauterine devices (IUDs) and implants: Education on and provision of contraceptive along with involvement of male partner had no significant impact on the use of condoms, OCs, IUDs and implants when compared to education alone (RR, 1.08; 95% CI, 0.88–1.26; one study, *n* = 39) (Analysis 5.3).

###### Maternal—Abortion

Education on sexual health and provision of contraceptive along with involvement of male partner had no significant impact on the repeat abortion rate when compared to education alone (RR, 0.32; 95% CI, 0.01–7.45; one study, *n* = 45) (Analysis 5.4).

No subgroups were possible as only single study was included in this comparison. This study (Zhu et al., 2009) did not report changes in knowledge and attitudes about the risk of unintended pregnancies, initiation of sexual intercourse and preterm birth.

3. Integration of family planning in maternal newborn care package

One quasi‐RCT (Baqui et al., 2018) provided family planning interventions with maternal and newborn care to increase birth spacing. The study did not report on un‐intended pregnancy, use of birth control method, but did report on preterm birth. The study reported lower risk of short birth interval (RR, 0.81; 95% CI, 0.69–0.95 and lower risk of preterm birth (RR, 0.79; 95% CI, 0.63–0.99) in the intervention arm compared to control areas.

##### Periconceptional folic acid

1. Supplementation with folic acid versus placebo

###### Primary outcomes

**Neonatal**

**Neural tube defects**

Of the five included studies, two assessed the impact of folic acid supplementation on NTDs when compared with placebo (Berry et al., 1999; Vergel et al., 1990). Of these studies, none were a RCT. Supplementation in these studies started in the preconception period and continued up till the first trimester of pregnancy. These studies had daily supplementation and the dosage of folic acid was 0.4 mg (Berry et al., 1999), and 5 mg (Vergel et al., 1990).

**Figure 8:**
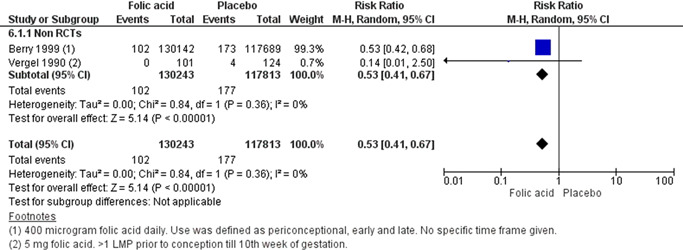
Forest plot of comparison: 20 folic acid versus placebo, outcome: 20.1 neural tube defects

**Figure 9:**
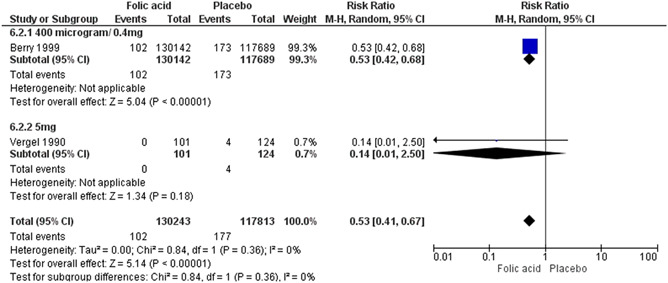
Forest plot of comparison: 20 folic acid versus placebo, outcome: 20.2 neural tube defects

We are uncertain whether periconceptional folic acid supplementation reduces the risk of NTD compared to placebo (RR, 0.53; 95% CI, 0.41–0.67; two studies; *n* = 248,056; random‐effect; heterogeneity: *χ* ^2^ *p *= .36; *I* ^2^ = 0%; very low certainty of evidence using GRADE assessment) (Analysis 6.1; Figure 8; Table 7). Impact of folic acid supplementation on NTDs was only significant for 0.4 mg of folic acid and nonsignificant for 5 mg of folic acid (Analysis 6.2; Figure 9).

**Table 7:** Periconceptional folic acid supplementation compared to placebo

Folic acid compared to placebo for periconceptional women						
Patient or population: Periconceptional women						
Setting: LMICs						
Intervention: Folic acid						
Comparison: Placebo						
Outcomes	Anticipated absolute effects^a^ (95% CI)		Relative effect (95% CI)	No. of participants (studies)	Certainty of the evidence (GRADE)	Comments
	Risk with placebo	Risk with folic acid				
Neural tube defects	Study population		RR 0.53 (0.41–0.67)	248,056 (2 RCTs)	⊕⊝⊝⊝ VERY LOW^b^, ^c^	
	2 per 1000	1 per 1000 (1–1)			

*Note*: GRADE Working Group grades of evidence: High certainty: We are very confident that the true effect lies close to that of the estimate of the effect. Moderate certainty: We are moderately confident in the effect estimate: The true effect is likely to be close to the estimate of the effect, but there is a possibility that it is substantially different. Low certainty: Our confidence in the effect estimate is limited: The true effect may be substantially different from the estimate of the effect. Very low certainty: We have very little confidence in the effect estimate: The true effect is likely to be substantially different from the estimate of effect.

Abbreviations: CI, confidence interval; OR, odds ratio; RR, risk ratio.

^a^ The risk in the intervention group (and its 95% confidence interval) is based on the assumed risk in the comparison group and the relative effect of the intervention (and its 95% CI).

^b^ Two studies (Berry, 1999) (Vergel et al., 1990) did not have random sequence generation and allocation concealment.

^c^ Number of events is <300.

###### Secondary outcomes

**Miscarriage**

Miscarriage was reported in one study Vergel et al., 1990. Vergel et al., 1990 reported miscarriages as examined versus not examined. In the not examined category, 6 of 124 of the unsupplemented participants and 1 of the 81 fully supplemented participants had a miscarriage. In the examined category, 1 of the 20 partially supplemented participants had a miscarriage (Table 8).

**Table 8:** Outcomes that could not be included in meta‐analysis (periconceptional folic acid supplementation)

Study ID	Type of study	Type of intervention	Outcomes
Berry (1999)	Cohort study	Folic acid supplementation	*Adherence* Defined as percen tage of folic acid pills taken as compared with the total number that could be taken. It was 78% in the North and 81% in the South. *Mean compliance (%)* Northern region Periconceptional use: 81 Late use: 75 Early discontinuation: 68 Total: 78 Southern region Periconceptional use: 87 Late use: 74 Early discontinuation: 78 Total: 81 *Stillbirth* 206/37,425 registered women in the North and 423/248,111 registered women in the South *Abortion* Induced (406/37,425 registered women in the North and 2704/248,111 registered women in the South Termination of pregnancy after prenatal diagnosis of birth defect (3/37,425 registered women in the North and 6/248,111 registered women in the South Miscarriage: Spontaneous (851/37,425 registered women in the North and 4317/248,111 registered women in the South
Li (2014)	Randomised controlled trial	Folic acid + milk OR milk alone OR folic acid alone	*Serum folate* Serum folic acid (nmol/l) in women (Han and Mongolian) who took 400 μg folic acid pre pregnancy versus control. Non pregnant group‐folic acid‐*Mongolian women* 17.8 ± 9.01 *Han women* 23.2 ± 7.77 No folic acid *Mongolian women* 14.2 ± 6.81 *Han women* 15.60 ± 6.19 5–7th week of gestation Folic acid‐*Mongolian women* 18.5 ± 5.29 *Han women* 24.6 ± 12.5 No folic acid *Mongolian women* 14.2 ± 6.81 *Han women* 15.5 ± 5.54 16th week of gestation Folic acid‐*Mongolian women* 17.8 ± 7.50 *Han women* 23.3 ± 13.1 No folic acid *Mongolian women* 15.8 ± 5.55 *Han women*16.3 ± 3.94 32 weeks of gestation Folic acid‐*Mongolian women* 14.9 ± 4.55 *Han women*16.9 ± 5.79 No folic acid *Mongolian women*15.4 ± 6.18 *Han women*14.7 ± 4.94
Rosenthal (2008)	Randomised controlled trial	Folic acid supplementation	*Adherence* Participants were divided into 58 compilers and 12 noncompilers.
Vergel et al. (1990)	Prospective cohort study	Folic acid supplementation	*RBC folate*: RBC folate levels (nmol/l) before and 2 weeks after stopping folic acid supplementation (mean ± *SD*) in fully supplemented participantsBefore: 808.5 ± 404.5After:14,190.3 ± 390.3.*Serum folate*: Serum folate levels (nmol/l) before and 2 weeks after stopping folic acid supplementation (mean ± *SD*) in fully supplemented participantsBefore: 23.9 ± 23.9After: 53.3 ± 20.4.*Adherence*: 81/101 women in the supplemented group completed the full regime of folic acid supplementation. *Miscarriage:* Miscarriage (not examined) Fully supplemented: 1 Partially supplemented: 0 Unsupplemented: 6 Miscarriages (Examined) Fully supplemented: 0 Partially supplemented: 1* Unsupplemented: 0
Wehby et al. (2012)	Randomised study	Folic acid supplementation	*Serum folate*: Mean post‐supplementation serum folate levels (ng/ml): 0.4 mg group—13.0 (increase of 1.7 ng/ml or 15% compared to baseline). 4 mg group—14.3 (increase of 3.1 ng/ml or 28% compared to baseline)(*p* < .0001). Mean post supplementation RBC folate (ng/ml): 0.4 mg group—716 (increase of 4 ng/ml compared to baseline). 4 mg group—793 (increase of 76 ng/ml compared to baseline). (*p* = .0021).

All the included studies in this comparison did not report on anaemia, iron deficiency anaemia, perinatal mortality, neonatal mortality, maternal serum folate, adverse effects, adherence to folic acid or iron folic acid supplementation, maternal mortality, preterm birth, small for gestational age, other congenital anomalies, or admission to special care for any cause.

Subgroup analysis for setting, timing of intervention and type of intervention could not be done. Setting was community for all the studies. All studies were conducted in the periconception period. The type of intervention was folic acid supplementation in all studies (Table 9).

**Table 9:** Matrix—Periconceptional folic acid supplementation

Study	Study design	Intervention	Duration	Dosage	Moderators of delivery	Setting	Timing of intervention
Berry (1999)	Cohort Study	Daily supplementation of folic acid supplementation	Premarital examination till end of first trimester of pregnancy. Women who registered with the pregnancy monitoring system from October 1993 to September 1995 and were pregnant any time from October 1, 1993, to December 31, 1996, were a part of this study	400 µg folic acid daily	Village health workers	Community. Hebei, Zheijang and Jiangsu provinces, China	Periconception use (before the LMP before conception to end of first trimester of pregnancy), late use (after the LMP before conception to end of first trimester of pregnancy) and early discontinuation (started and stopped before LMP before conception)
Li (2014)	Parallel group design (randomised controlled trial)	Supplementation of folic acid + milk; milk alone; folic acid alone	Folic acid supplementation was from the 3 months before to 3 months after conception. Milk was supplemented after pregnancy was confirmed till delivery	Tablets had 400 µg folic acid each, one was to be taken daily. Average level of folate in the milk was 3.79 µg/100 ml (ranging from 1.68–5.69 µg/100 ml). 243 ml of milk were provided daily.	Trial organisers	Rural and urban maternal and child health centres. Tongliao city, China	Periconceptional, Gestational
Rosenthal (2008)	Double‐blind randomised controlled trial	Daily and weekly supplementation of folic acid tablet	12 weeks	1 mg folic acid daily; 5 mg folic acid once weekly	Factory nurses	Factories. Choloma city, Honduras	Pregestational
Vergel et al. (1990)	Prospective cohort study	Daily supplementation of folic acid	>1 LMP prior to conception till 10th week of gestation	5 mg folic acid	Researchers	Provincial Genetics Department. Havana City, Cuba	Periconceptional
Wehby et al. (2012)	Randomised study	Daily supplementation of folic acid	Before pregnancy till the end of the first trimester	4 mg folic acid; 0.4 mg folic acid	Study staff	Craniofacial clinics. Brazil	Preconception, Periconceptional

#### 5.2.8 Periconceptional iron folic acid

1. Supplementation with iron‐folic acid versus placebo:

##### Primary outcomes

###### Maternal

**Anaemia**

**Figure 10:**
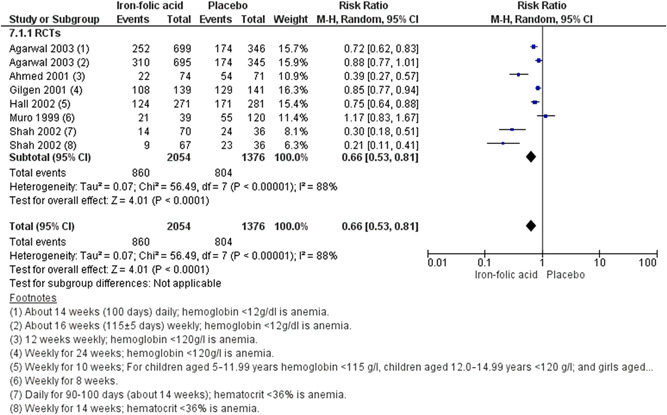
Forest plot of comparison: 22 iron folic acid versus placebo, outcome: 22.1 anaemia

Out of a total of 10 studies, six presented anaemia in iron‐folic acid group compared to placebo as an outcome (Agarwal et al., 2003; Ahmed et al., 2001; Gilgen, 2001; Hall et al., 2002; Muro et al., 1999; Shah and Gupta, 2002). All of these were RCTs. All of them had weekly supplementation and Agarwal et al. (2003) and Shah and Gupta, 2002 also had daily supplementation arms.The studies were grouped according to type, setting, weekly or daily supplementation, and duration of weekly and daily supplementation. We are uncertain whether iron‐folic acid supplementationreduces anaemia (RR, 0.66; 95% CI, 0.53–0.81; six studies; *n* = 3430, random‐effect; heterogeneity: *χ* ^2^ *p* < 0.001; *I* ^2^ = 88%; very low certainty of evidence using GRADE assessment) (Analysis 7.1; Figure 10; Table 10).

**Table 10:** Periconceptional iron folic acid supplementation compared to placebo

Iron folic acid compared to placebo for periconceptional women						
Patient or population: Periconceptional women						
Setting: LMICs						
Intervention: Iron folic acid						
Comparison: Placebo						
Outcomes	Anticipated absolute effects^a^ (95% CI)		Relative effect (95% CI)	No. of participants (studies)	Certainty of the evidence (GRADE)	Comments
	Risk with placebo	Risk with iron folic acid				
Anaemia—RCTs	Study population		RR 0.66 (0.53–0.81)	3430 (6 RCTs)	⊕⊝⊝⊝ VERY LOW^b^, ^c^, ^d^	
	565 per 1000	350 per 1000 (288–429)			
Anaemia—Weekly supplementation	Study population		RR 0.70 (0.55–0.88)	2661 (6 RCTs)	⊕⊝⊝⊝ VERY LOW^b^, ^c^, ^g^	
	488 per 1000	332 per 1000 (273–405)				
Anaemia—Daily supplementation	Study population		RR 0.49 (0.21–1.12)	1532 (2 RCTs)	⊕⊝⊝⊝ VERY LOW^b^, ^c^, ^h^	
	417 per 1000	213 per 1000 (133–338)			
Anaemia—8 weeks of weekly supplementation	Study population		RR 1.17 (0.55–1.67)	159 (1 RCTs)	⊕⊝⊝⊝ VERY LOW^e^, ^f^, ^i^	
	249 per 1000	237 per 1000(142–394)				
Anaemia—10 weeks of weekly supplementation	Study population		RR 0.75 (0.64–0.88)	552 (1 RCT)	⊕⊕⊝⊝ VERY LOW^e^, ^j^	
	609 per 1000	456 per 1000 (389–536)			
Anaemia—12 weeks of weekly supplementation	Study population		RR 0.39 (0.27–0.57)	145 (1 RCTs)	⊕⊝⊝⊝VERY LOW^b^, ^c^, ^e^, ^h^	
	398 per 1000	187 per 1000 (108–327)				
Anaemia—14 weeks of weekly supplementation	Study population		RR 0.21 (0.11–0.39)	139 (1 RCT)	⊕⊕⊝⊝ LOW^e^, ^k^	
	653 per 1000	137 per 1000 (72–255)			
Anaemia—16 weeks of weekly supplementation	Study population		RR 0.89 (0.79–0.99)	1386 (1 RCT)	⊕⊕⊕⊝ MODERATE^j^	
	504 per 1000	448 per 1000 (398–499)				
Anaemia—24 weeks of weekly supplementation	Study population		RR 0.85 (0.77–0.94)	280 (1 RCT)	⊕⊕⊝⊝ LOW^e^, ^l^	
	915 per 1000	778 per 1000 (704–860)			
Anaemia—School	Study population		RR 0.66 (0.51–0.86)	3005 (4 RCTs)	⊕⊝⊝⊝ VERY LOW^b^, ^c^, ^m^	
	459 per 1000	257 per 1000 (206–326)				
Anaemia—Work	Study population		RR 0.59 (0.24–1.43)	425 (2 RCTs)	⊕⊝⊝⊝VERY LOW^e^, ^n^, ^o^	
	863 per 1000	509 per 1000 (207–1000)			

*Note*: GRADE Working Group grades of evidence: High certainty: We are very confident that the true effect lies close to that of the estimate of the effect. Moderate certainty: We are moderately confident in the effect estimate: The true effect is likely to be close to the estimate of the effect, but there is a possibility that it is substantially different. Low certainty: Our confidence in the effect estimate is limited: The true effect may be substantially different from the estimate of the effect. Very low certainty: We have very little confidence in the effect estimate: The true effect is likely to be substantially different from the estimate of effect.

Abbreviations: CI, confidence interval; OR, odds ratio; RR, risk ratio.

^a^ The risk in the intervention group (and its 95% CI) is based on the assumed risk in the comparison group and the relative effect of the intervention (and its 95% CI).

^b^ Some studies use multiple micro nutrients in the intervention arm.

^c^ Multiple studies with large weight are at high risk for bias.

^d^ Heterogeneity is 84%.

^e^ Total number of events is <300.

^f^ One study uses vitamin C along with iron‐folic acid in the intervention arm.

^g^ Heterogeneity is 82%.

^h^ Heterogeneity is 76%.

^i^ One study is at high risk of bias.

^j^ Study is at risk of performance and reporting bias.

^k^ Study is at risk of other biases.

^l^ It is mostly unclear if study is at risk of bias.

^m^ Heterogeneity is 83%.

^n^ Heterogeneity is 95%.

^o^ One study is at risk of attrition bias.

**Figure 11:**
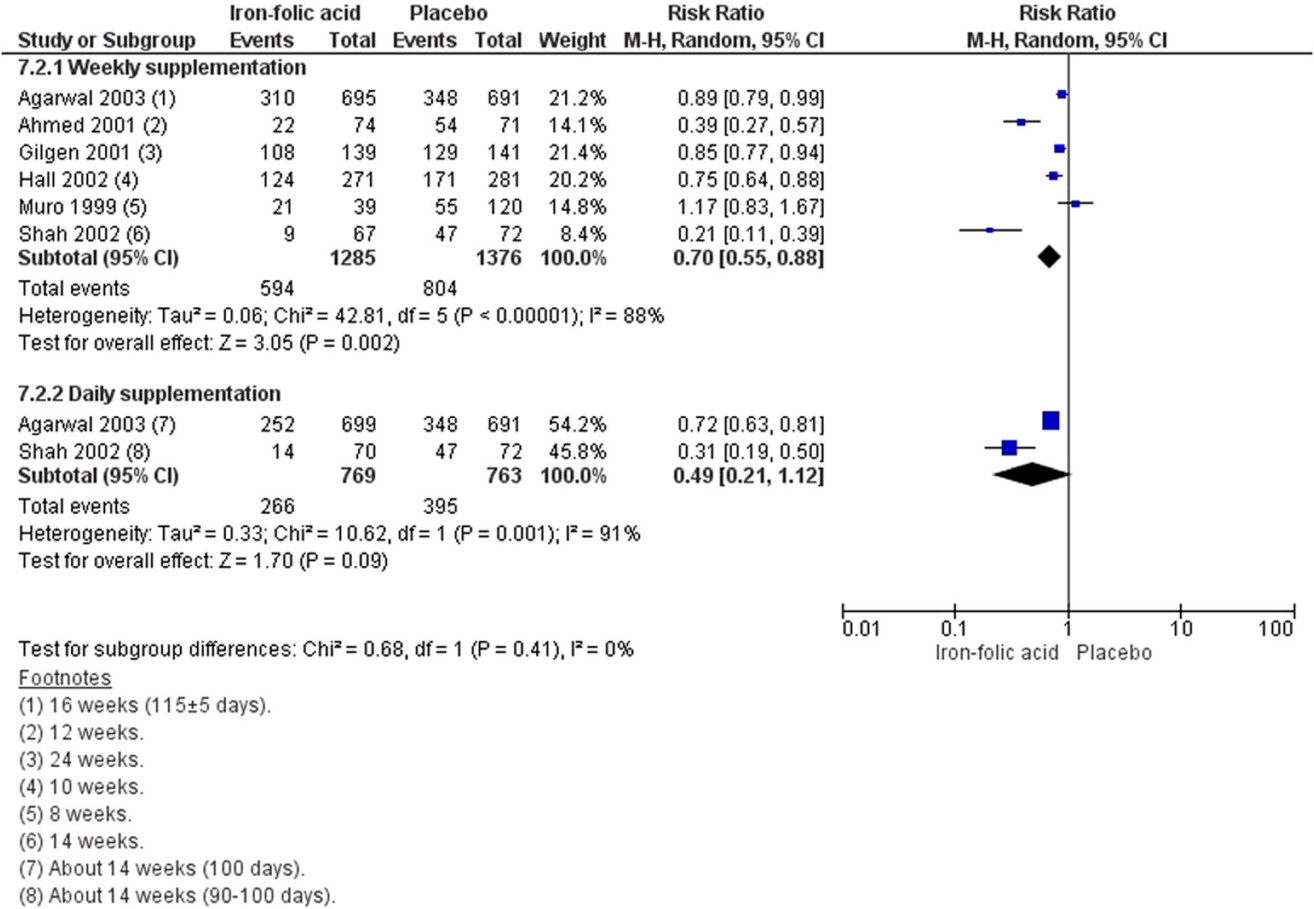
Forest plot of comparison: 22 iron folic acid versus placebo, outcome: 22.2 anaemia

We are uncertain whether weekly (RR, 0.70; 95% CI 0.55–0.88; six studies; *n* = 2661; random‐effect; heterogeneity: *χ* ^2^ *p* < .001; *I* ^2^ = 88%; very low certainty of evidence using GRADE assessments) or daily supplementation of iron‐folic acid (RR, 0.49; 95% CI, 0.21–1.12; two studies; *n* = 1532; random‐effect; heterogeneity: *χ* ^2^ *p* = .001; *I* ^2^ = 91%; very low certainty of evidence using GRADE assessments) reduce anaemia compared to a placebo (Analysis 7.2; Figure 11; Table 10).

**Figure 12:**
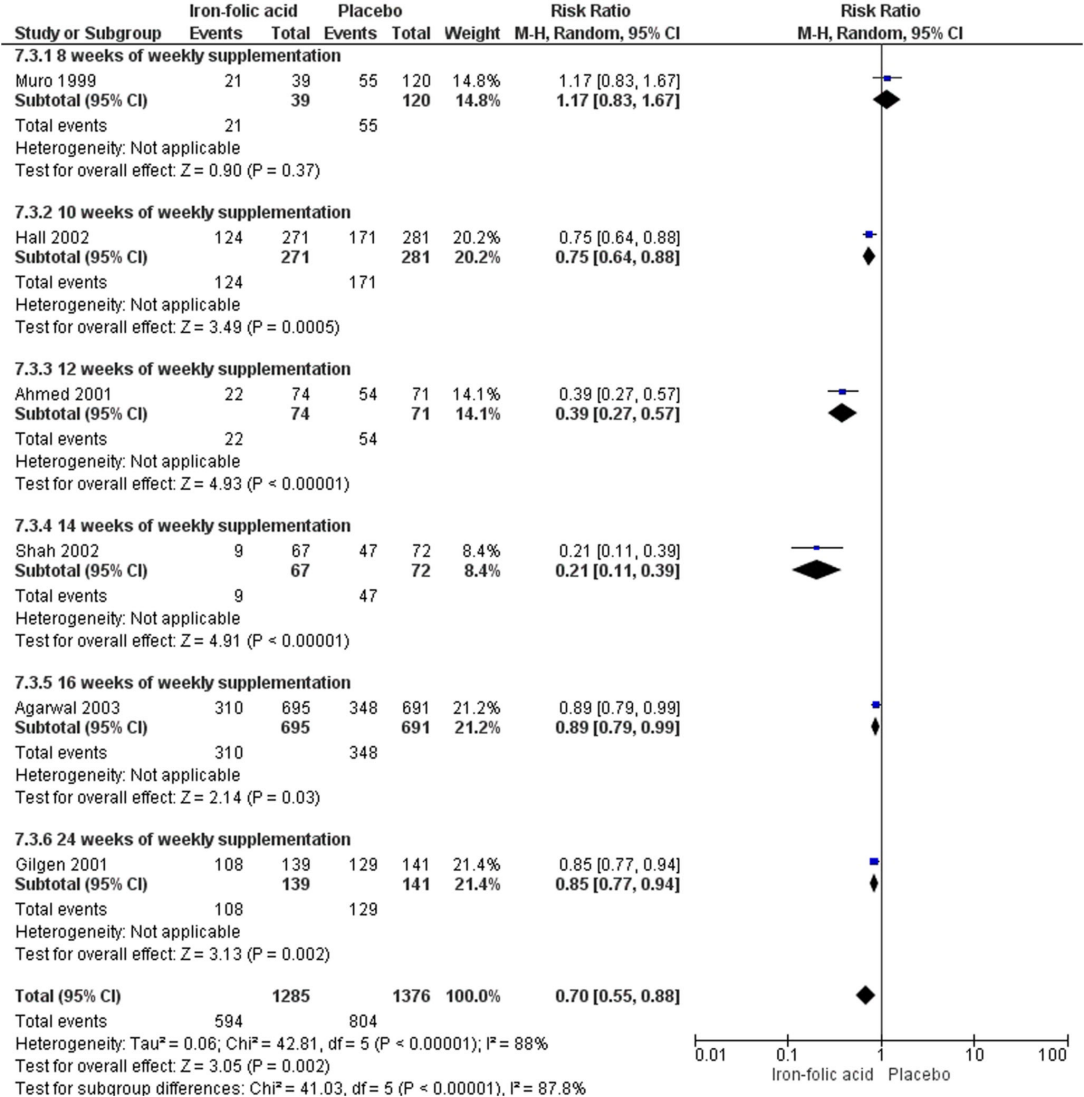
Forest plot of comparison: 22 iron folic acid versus placebo, outcome: 22.3 anaemia

Evidence from single studies on 8 weeks weekly supplementation of iron‐folic acid sis not show improvement in anaemia (RR, 1.17; 95% CI, 0.83–1.67), whereas 10, 12, 14, 16, and 24 weeks weekly supplementation showed significant improvement in anaemia rates by 25%, 41%, 39%, 11%, and 15% (Analysis 7.3; Figure 12; Table 10).

**Figure 13:**
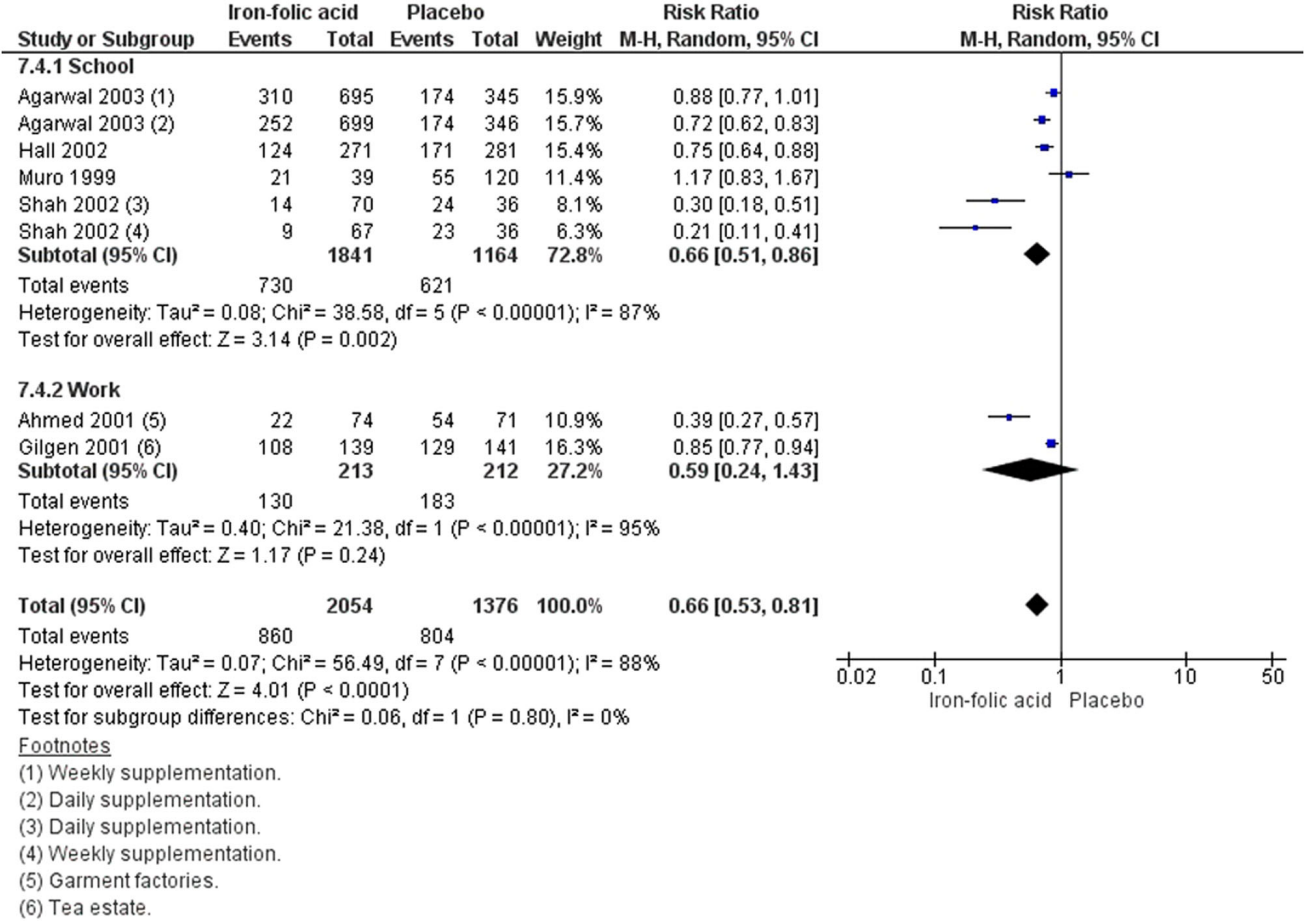
Forest plot of comparison: 22 iron folic acid versus placebo, outcome: 22.5 anaemia

We are uncertain whether iron‐folic acid supplementation in school setting (RR, 0.66; 95% CI, 0.51–0.86; four studies; *n* = 3005; random‐effect; heterogeneity: *χ* ^2^ *p* < .0001; *I* ^2^ = 87%; very low certainty of evidence using GRADE assessment) and at work setting (RR, 0.59; 95% CI, 0.24–1.43; two studies; *n* = 425; random‐effect; very low certainty of evidence using GRADE assessment) reduces anaemia (Analysis 7.4; Figure 13; Table 10).

##### Secondary outcomes

###### Adverse effect

Five studies reported adverse effects (Agarwal et al., 2003; Gilgen, 2001; Muro et al., 1999; Shah & Gupta, 2002; Soekarjo et al., 2004). In Agarwal et al. (2003), seven girls in the daily supplementation group had gastric side effects in the second week of the intervention and were excluded from the trial on request (Table 11). Four girls receiving iron‐folic acid supplementation reported stomach irritation in Muro et al. (1999) (Table 11). Two of the girls were only receiving supplementation while two were receiving communication sessions along with the supplementation. Shah and Gupta (2002) mentions that 2 out of 70 girls in the once daily supplementation group reported adverse effects (Table 11). In Soekarjo et al. (2004), 41.4% of the 134 girls interviewed about taking iron‐folic acid supplementation reported gastrointestinal side effects (Table 11).

**Table 11:** Outcomes that could not be included in meta‐analysis (Periconceptional iron folic acid supplementation)

Study ID	Type of study	Type of intervention	Outcome	
			Adverse effects	Adherence
Agarwal (2003)	Randomised controlled trial	Iron and folic acid supplementation	Seven girls in the daily supplemented group were excluded because they had gastric side effects and did not want to continue the study.	
Ahmed et al. (2001)	Randomised controlled trial	Supplements of placebo, vitamin A, iron + folic acid, or iron + folic acid + vitamin A weekly		Percentage of people that received certain number of dosages in the iron + folic acid group‐12 doses‐ 60% 11 doses—24% 10 doses—14% 9 doses—2%
Kanani (2000)	Quasi randomised study	Supplements Iron folic acid		90% of the girls consumed >85 of the 90 tablets
Muro et al. (1999)	Randomised controlled trial	(1) Iron‐folic acid only weekly. (2) Iron‐folic acid with weekly communication sessions (3) Control: no intervention	Stomach irritation School 1—1 girl School 2—1 girl School 3—2 girls Increased appetite School 1—5 girls School 2—3 girls	Reported Compliance percentage School 1 1st dose—87% were compliant 2nd dose—69% 3rd dose—90% 4th dose—100% 5th dose—100% 6th dose—95% 7th dose—90% 8th dose—85% School 2 1st dose—(not available) 2nd dose—(not available) 3rd dose—(not available) 4th dose—74% 5th dose—59% 6thdose—(not available) 7th dose—(not available) 8th dose—90% School 3 1st dose—56% 2nd dose—28% 3rd dose—67% 4th dose—90% 5th dose—51% 6thdose—51% 7th dose—28% 8th dose—18% Observed compliance (positive iron stool test) School 1 Third week—100% Fifth week—87% School 2 Third week‐ 93% Fifth week‐56% School 3 Third week‐ 60% Fifth week‐ 40%
Shah and Gupta (2002)	Randomised controlled trial	Weekly versus daily iron and folic acid supplementation	Adverse effects in 2 girls—both in once daily group Not mentioned what adverse effects	Noncompliant number (%) Group A 8 (11.4) Group B 4 (6.0) Group C 4 (5.6)
Soekarjo et al. (2004)	Randomised controlled trial	Weekly iron‐folic acid supplementation versus control	41.4% of 134 girls interviewed from the iron‐folic acid supplementation group reported GI side effects	

Gilgen 2001 found no difference in adverse effects in iron‐folic acid supplementation group compared to placebo group (RR, 0.63; 95% CI, 0.38–1.05; one study; *n* = 280) (Analysis 7.5)

###### Adherence to folic acid or iron‐folic acid supplementation

Four studies examined adherence to iron‐folic acid supplementation (Ahmed et al., 2001; Kanani, 2000; Muro et al., 1999; Shah & Gupta, 2002). Ahmed et al. (2001) reported that, in the iron‐folic acid group, 60% received 12 doses, 24% received 11 doses, 14% received 10 doses and 2% received 9 doses. In the placebo group, 68% received 12 doses, 24% received 11 doses, 6% received 10 doses and 2% received nine doses (Table 11). According to Kanani (2000), 90% of the girls consumed >85 of the 90 tablets provided (Table 11). Muro et al. (1999) provided reported as well as observed compliance to the iron‐folic acid supplementation. Reported compliance was 90% in school 1, 89% in school 2 and 48% in school 3. Observed compliance was 94% in school 1, 75% in school 2 and 50% in school 3. Reported compliance was also given week wise in all the schools (Table 11). In Shah and Gupta (2002), eight participants (11.4%) in the daily supplemented group, four (6.0%) in the weekly and four (5.6%) in the control group were non complaint (Table 11).

In this comparison, we could not assess the following primary and secondary outcomes: iron deficiency anaemia, NTDs, stillbirth, perinatal mortality, neonatal mortality, low birth weight, maternal red blood cell folate, maternal serum folate, abortion, miscarriage, maternal mortality, preterm birth, small for gestational age, other congenital anomalies, and admission to special care for any cause.

Subgroup analysis for timing of intervention and type of intervention could not be done. Timing of intervention was preconception for all studies. The type of intervention was iron‐folic acid supplementation for all studies (Table 12).

**Table 12:** Matrix—Periconceptional iron folic acid supplementation

Study	Study design	Intervention	Duration	Dosage	Moderators of delivery	Setting	Timing of intervention
Agarwal 2003	RCT	Daily and weekly supplements of iron‐folic acid	115 days and at 230 ± 5 days	100 mg iron and 500 µg folate daily and weekly		Government schools. North‐East Delhi, India	Preconception
Ahmed et al. (2001)	RCT	Supplements of placebo; vitamin A; iron‐folic acid; or iron + folic acid + vitamin A weekly	12 weeks	2.42 mg vitamin A (retinol) as retinyl palmitate, 120 mg elemental ferrous sulphate, and 3.5 mg folic acid	Field staff	Garment factories. Dhaka City, Bangladesh	Preconception
Februhartanty 2002	RCT (with a nonrandomised third arm)	Weekly supplements of iron‐folic acid; iron‐folic acid supplements for four days during menstruation	16 weeks	60 mg elemental iron and 0.25 mg folic acid in the form of 200 mg ferrous sulphate	Teachers	Schools. Kupang, East Nusa Tenggara, Indonesia	Preconception
Hall et al. (2002)	RCT	Weekly supplements of iron‐folic acid	10 weeks	65 mg of iron and 0.25 mg of folic acid	Teachers	Schools. Kolondieba district, Mali	Preconception
Kanani 2000	Quasi randomised study	Daily supplements iron‐folic acid	3 months	60 mg elemental iron and 0.5 mg folic acid per day		Community. Vadodora, India	Preconception
Gilgen (2001)	RCT	Weekly supplements of iron‐folic acid	24 weeks	200 mg ferrous fumarate and 200 mg folic acid		Community (tea estate). Bangladesh	Preconception
Muro et al. (1999)	RCT	Weekly iron‐folic acid supplementation; weekly iron folic acid supplementation along with communication session.	8 weeks	65 mg iron sulphate and 0.25 mg folic acid	Teachers	Schools. Dar‐es‐Salaam, Tanzania	Preconception
Shah and Gupta (2002)	RCT	Weekly versus daily iron‐folic acid supplementation.	90–100 days for once daily; 14 weeks for weekly	350 mg ferrous sulphate and 1.5 mg folic acid combination	Group A‐ Parents. Group B‐ Investigator.	School. Dharan, Nepal	Preconception
Shobha and Sharada (2003)	RCT	Daily or twice weekly iron + folic acid supplementation regimen	84 days (i.e., 12 weeks).	60 mg iron and 0.5 mg folic acid	Investigator	School. Ranga Reddy district, India	Preconception
Soekarjo et al. (2004)	RCT	Weekly supplements of vitamin A; iron and folate; vitamin A, iron and folate	14 weeks	10 000 IU vitamin A and 60 mg elemental iron plus 250 mg folate	Field workers	Schools and homes. Bangkalan and Sampang district, Indonesia	Preconception

## 6 DISCUSSION

### 6.1 Summary of main results

For the interventions to delay the age of first pregnancy, our review found three comparisons comprising of different interventions related to delaying age at first pregnancy. Education on sexual health and contraception was the most common intervention and use of birth control methods was the most reported outcome. Education on sexual health and contraception intervention alone showed an insignificant impact on the risk of unintended pregnancies. However, it showed a showed significant increase in use of condom (ever) by 71%.The evidence on interventions on education and provision of contraception came from single study showed a significant impact on improving the the use of any contraception by 49% and use of condoms by 14%.

Interventions on optimising interpregnancy intervals did not show a significant impact of education on sexual health and provision of contraceptive along with involvement of male partner on the risk of unintended pregnancies when compared to education on sexual health and contraception only. However, educational intervention alone or with provision of contraceptive showed a significant improvement in the uptake of contraceptive method.

Overall, use of folic acid reduces the incidence of NTDs by 47% but the evidence came from non RCTs and on GADE the certainty of this evidence is very low. The impact of folic acid supplementation on NTDs differs by folic acid dosage and the evidence from single study with the larger sample size showed significant impact for 0.4 mg (reduction in incidence of NTDs by 47%) and a nonsignificant impact at 4 and 5 mg. Included studies did not report on anaemia, iron deficiency anaemia, perinatal mortality, neonatal mortality, adverse effects, maternal mortality, preterm birth, small for gestational age, and admission to special care for any cause.

Use of iron‐folic acid reduces the prevalence of anaemia compared to a placebo by 34%. However, it is important to note that the evidence came from RCTs and supplemented adolescent and young girls during preconception period only and the certainty of the evidence was very low on GRADE. Weekly supplementation showed significant improvement in reducing anaemia by 30% but the certainty of the evidence was very low on GRADE. Supplementation at school showed significant improvement in reducing anaemia rates by 34% but again the certainty of the evidence was very low. The evidence does not support any significant difference between use of iron‐folic acid and placebo to reduce adverse effects. Included studies did not report on iron deficiency anaemia, NTDs, stillbirth, perinatal mortality, neonatal mortality, low birth weight, abortion, miscarriage, maternal mortality, preterm birth, small for gestational age, other congenital anomalies and admission to special care for any cause.

Across all the included intervention objectives of this review, there was a large amount of heterogeneity across the studies which impacts the applicability and reliabiltiy of the results of this review. Readers should consider the heterogenous nature of the studies included in the analyses and the implications this has on the quality of evidence and therefore the results should be interpreted with cautions.

### 6.2 Overall completeness and applicability of evidence

Although an extensive search was undertaken to locate relevant papers there are still some knowledge gaps that remain. The greatest amount of studies was available for interventions to delay the age at first pregnancy and while these studies provided us with insight into which intervention settings were most effective, the majority of interventions were education on sexual health focused and therefore do not sufficiently capture other intervention strategies. This was also the case for studies related to optimising interpregnancy intervals with health education being the dominating intervention strategy. A commonality across all the interventions is that not all of the outcomes noted in the methods were reported on in the included studies. Another part of this review's objective was to assess the effectiveness of these interventions in LMICs, however the literature included in this review is dominated by African and Asian countries. Therefore, while this review provides an appropriate overview of the current evidence it is important to acknowledge that the findings may not be valid to all settings and populations. Due to limited number of studies in each comparison, we could not conduct all the planned subgroup analysis and sensitivity analysis.

### 6.3 Quality of the evidence

Forty‐three studies were included in this review, with majority contributing to the evidence for delaying the age of pregnancy (*N* = 26), with much fewer identified for optimising interpregnancy birth intervals (*N* = 4), folic acid supplementation (*N* = 5) and iron‐folic acid supplementation (*N* = 10). Across all of the studies there were concerns relating to different types of bias and there was a large amount of heterogeneity which made some planned analyses impossible to execute.

For studies specific to delaying the age at first pregnancy, the blinding of participants was often not possible due to the nature of the intervention and most studies did not mention whether or not the assessors were blinded. Baseline outcomes were usually similar within studies, while characteristics were either similar or unclear about it. The majority of studies in this section did not mention whether knowledge of allocated interventions was adequately prevented and did not address the issue of incomplete outcome data. Overall, contamination was adequately prevented, selective reporting was avoided and other sources of bias were minimal. The overall quality of events primary outcomes was either low or very low. Specific to unintended pregnancy, the evidence was evaluated using GRADE and it was determined that the evidence for this outcome was low due to issues of attrition and selection bias.

In prolonging inter‐pregnancy interval, participants could not be blinded to the intervention given its nature. Randomisation was not employed throughout given the study designs and its details were often not mentioned which impacts quality assessment. In one instance baseline outcome measurements were different in intervention and control groups. The overall quality of events primary outcomes was either low or very low. For the primary outcome of unintended pregnancy the evidence was deemed to be moderate, however this rating was based off of one study with a small sample size and therefore should be interpretated with caution.

In periconceptional folic acid supplementation, most of the studies were either low or unclear risk of bias considering allocation concealment, blinding, incomplete outcome data, selective reporting and other biases. In most studies, either random sequence generation was not adequate or the method of random sequence generation was not described. The overall quality of events primary outcomes was either low or very low. Concerning NTDs, the quality of evidence was considered to be very low due to the lack of random sequence generation and allocation concealment in the two contributing studies.

In iron‐folic acid supplementation, most of the studies were either low or unclear risk of bias considering allocation concealment, incomplete outcome data, selective reporting and other biases. In most studies, either random sequence generation was not adequate or the method of random sequence generation was not described. Blinding of participants and personnel was mostly at high risk of bias while blinding of outcome assessors was either low risk or unclear risk of bias. Publication bias was considered unlikely in all the studies. The overall quality of primary outcomes was either low or very low. The GRADE assessment resulted in a range of evidence ratings from very low to moderate for the outcome anaemia, which was dependent on the difference strategies of supplementation. The issues with the quality of evidence is largely due to heterogeniety, which is a common factor across all of the interventions investigated in this review. The review could not undertake sensitivity analysis as there were very few studies under each outcome.

None of the pooled outcomes has 10 ore more studies, therefore publication bias was not assessed.

### 6.4 Potential biases in the review process

Efforts were made to reduce biases in the review process. Two reviewers independently checked for eligibility for inclusion, performed data extractions, assessed for risk of bias and entered the data. However, different types of study, for example, quasi RCTs are a potential source of bias in the review. Quasi studies were included in this review along with RCTs which are prone to error given their respective designs. Cluster randomised trials were a part of this study and their results had to be adjusted accordingly to compare them with the other studies. Where possible intra‐cluster coefficients (ICCs) from the study itself were used that was given by its own authors but this was not always mentioned and in those cases an estimate of the ICCs was taken from a study with a similar setting. This was done to the best of the author's abilities but the accuracy can be questionable. Due to the variety of interventions and the way study outcomes were measured, a large number of studies could not be pooled together often in this review which is a limitation.

However, carrying out reviews is not an exact science and may require a number of subjective judgements; it is possible that a different review team may have reached different decisions regarding assessments of eligibility and risk of bias. We would encourage readers to examine the Characteristics of included studies tables to assist in the interpretation of results.

### 6.5 Agreements and disagreements with other studies or reviews

The results that interventions such as provision of contraceptives and education to delay the age at first pregnancy are consistent to earlier reviews (Hindin 2016; Oringanje et al. 2016; Salam et al., 2016; Whitaker et al., 2016 and Norton et al., 2017). Hindin (2016) included 21 studies from LMICs found increased contraceptive use and delay in the age of sexual debut after interventions to either prevent unintended pregnancies or repeat pregnancies. Similr to our review, Oringanje et al. (2016) that included 53 studies from LMICs and HICs also did not find evidence on reducing the risk of unintended pregnancies. Similar to our review Salam et al. (2016) and Norton et al. (2017) also showed that education interventions were effective in bringing about significant improvement in sexual knowledge, contraceptive use, as well as in decreasing adolescent pregnancy and improving birth intervals.

The result that periconceptional folic acid supplementation reduces the incidence of NTDs is consistent with other earlier reviews (Dean et al., 2014b; De‐Regil et al., 2015; Ramakrishnan 2012), but our review includes the addition of quasi‐experimental studies to the evidence from LMICs only. The results of our review on periconceptional iron‐folic acid supplementation are in agreement with Fernández‐Gaxiola and De‐Regil (2011) although that review only assessed use of iron among menstruating women (alone or with other micronutrients) in reducing the prevalence of anaemia regardless of dose used. However, unlike Fernández‐Gaxiola and De‐Regil (2011), our review shows that reduction in anaemia prevalence differs with the duration of iron folic acid supplementation (no significant impact <8 weeks).

Overall, the findings from our review are similar to earlier reviews and our findings can be extrapolated to LMICs in general. However, we have to be cautious while drawing conclusions since studies had multiple interventions for interventions alongside education (i.e., training of health workers, provision of referrals, social marketing of contraception and involvement of other family members) to delay the age of first pregnancy and prolonging birth interval. Although a large number of studies were included, a large number of studies could not be pooled together since the respective interventions were quite heterogeneous in nature. This calls for further research on these interventions in LMICs. This allows a more robust analysis to take place in the future that may help elucidate which interventions are most effective. Education regarding sexual health is clearly a powerful tool that has been seen to be significantly effective in improving maternal outcomes but more information on mode of delivery, setting and nature of para‐interventions needs to be present to make recommendations.

## 7 AUTHORS' CONCLUSIONS

### 7.1 Implications for practice

Our review has found that SRH educational interventions that aim to delay pregnancy or optimise interpregnancy intervals can benefit in improving the contraceptive use and knowledge but there is insufficient evidence for the impact on unplanned pregnancy which may be due to the lack of included studies reporting on this outcome. Educational interventions aiming to optimise interpregnancy intervals were more effective than provision of contraceptives, however there were limited studies included in these analyses so intervention strategies need to be investigated further to determine the most effective approach.

Regarding folic acid supplementation our review further supports the use of folic acid to reduce NTDs, however GRADE assessment determined that there is very low certainty of evidence. Our review also supports the use of iron‐folic acid during the preconception period to reduce anaemia where weekly supplementation regimes were more effective however on GRADE the certainty of the evidence was very low There is evidence that iron‐folic acid supplementation is most effective when supplementation takes place in school settings compared to work settings, perhaps because students are more easily supervised and therefore adherence is more consistent. It is important to note that certainty of the evidence is very low on GRADE

The review, however, has identified a large amount of heterogeneity across the studies which impacts the applicability and reliabiltiy of the results and therefore the results should be interpreted with cautions.

### 7.2 Implications for research

Further evidence is required for each of the intervention targets in this review. Firstly, education on sexual health and contraception interventions that aim to delay pregnancy or improve interpregnancy intervals need to more consistently report on unplanned pregnancy in adolescent population particularly in LMICs. While there is known challenges to reporting on unplanned pregnancies, especially when self‐reported. Further research is required to determine whether school, community, clinical or a combination of settings is optimal for these interventions, though there is evidence from our review that studies in community settings were more effective. With reproductive and sexual health education often emphasising the importance of avoiding unplanned pregnancies, studies need to ensure that these education on sexual health and contraception interventions are translating into behaviour beyond contraceptive use to include the prevalence of unplanned pregnancies. Majority of the interventions had a education on sexual health and contraception component, while there is some adequate evidence from our meta‐analyses of the use of health education strategies, there were fewer studies with multidimensional components (such as health education and provision of contraceptives) and therefore these strategies need to be further investigated to determine whether they are just as effective or better than solely education.

Our review was consistent with other reviews that folic acid and iron‐folic acid supplementation reduces the incidence of NTDs and anaemia respectively and therefore supplementation should be implemented before conception but certainty of the evidence was low. More robust research is needed in LMICs to determine the best strategies to ensure consistent supplementation use of folic acid and iron‐folic acid in order to determine which dosages and durations are most beneficial for maternal and neonatal outcomes. Regardless of study design, anthropometric measures should be collected when possible to determine the effect of micronutrient supplementation on related outcomes as this review could not set out to report on neonatal outcomes such as birth weight, and SGA. We included an array of study designs, therefore it is unsurprising that the GRADE assessment varied from medium to very low quality.

## CONTRIBUTIONS OF AUTHORS

Zohra S Lassi and Sophie G. E. Kedzior ran the searches and performed the initial searching. Wajeeha Tariq, Yamna Jadoon, and Sophie G. E. Kedzior have done with extraction, quality assessment of all included studies. Wajeeha Tariq, Yamna Jadoon, has done the analysis in supervision of Zohra S Lassi. Wajeeha Tariq, Yamna Jadoon, Sophie G. E. Kedzior, and Zohra S. Lassi have written the review. Jai K. Das and Zulfiqar A. Bhutta have critically read the manuscript and Zohra S. Lassi finalised the review.

## DECLARATIONS OF INTEREST

The authors are not aware of any conflicts of interest arising from financial or researcher interests.

## DIFFERENCES BETWEEN PROTOCOL AND REVIEW

We did not state a priori which measurement time points would be used for synthesising the outcomes, therefore used the time points which were most commonly reported and was meaningful for the intervention(s) under study.

## PUBLISHED NOTES

Not all meta‐analyses figures are displayed due to space limitations.

## CHARACTERISTICS OF STUDIES

Characteristics of included studies

Agarwal (2003)

**Table:** 

**Methods**	RCT in northeast Delhi. Study was conducted during August 1996 to February 1999
**Participants**	Adolescent school girls (*n* = 2088) 10–17 years, 702 on daily and 695 on weekly iron‐folate administration
**Interventions**	Intervention: weekly or daily iron‐folate supplementation, 100 mg elemental iron and 500 µg of folic Control: 691 girls served as controls; no supplementation for first 100 days, then same as daily group Setting: school‐level Timing of intervention: preconception Moderators delivering: researchers (?)
**Outcomes**	Primary: Anaemia, haemoglobin concentration; plasma ferritin Secondary: Sexual maturity rating was done using Tanner's criteria, time of menarche and regularity of menstrual periods was noted
**Notes**	Funding: UNICEF, New Delhi. Declaration of interest: None stated.

Risk of bias table

**Table:** 

Bias	Authors' judgement	Support for judgement
Random sequence generation (selection bias)	Unclear risk	Quote: “The randomisation was done at the class section level for 60 class sections.” Comment: method of sequence generation was not described
Allocation concealment (selection bias)	Low risk	Comment: since randomisation was performed at class section level it is unlikely a selection bias is present at individual level
Similar baseline characteristics	Unclear risk	
Similar baseline outcome measurement	Unclear risk	
Blinding of participants and personnel (performance bias)	High risk	Comment: all groups received the supplement and no placebo was used
Blinding of outcome assessment (detection bias)	Low risk	Comment: outcomes were objective therefore the blinding of outcome assessors would not impact the results
Incomplete outcome data (attrition bias)	Low risk	Quote: “Seven girls in the daily administered group during the second week of intervention complained of gastric side effects and requested not to continue in the study and were excluded” Comment: incomplete outcome data were matched across groups.
Prevention of knowledge of allocated intervention	Unclear risk	
Protection against contamination	Unclear risk	
Selective reporting (reporting bias)	Unclear risk	Quote: “Girls with haemoglobin <70 g/L (0.3%)were eliminated from the analysis.” “Plasma ferritin and C‐reactive proteins (CRP) were estimated in every tenth girl of the study groups” Comment: it is unclear how each girl was selected for these analyses. Data not available for the second measurement. However no protocol could be found and therefore there is insufficient evidence to make a judgement.
Other bias	Low risk	Comment: the study appears to be free of other bias

Ahmed et al. (2001)

**Table:** 

**Methods**	Randomised, double‐blind, placebo‐controlled trial in urban Bangladesh. Study was from March to September 1998
**Participants**	Postmenarcheal, nonpregnant teenagers aged 14–19 years. 120 subjects were recruited to each of the 4 groups to give a total study population of 480 subjects
**Interventions**	Intervention: The supplements contained 2.42 mg vitamin A (retinol) as retinyl palmitate, 120 mg elemental Fe as ferrous sulfate, and 3.5 mg folic acid. There were 3 study groups: (1) vitamin A only (2.42 mg retinol as retinyl palmitate and a placebo for iron and folic acid), (2) iron + folic acid (120 mg elemental Fe as ferrous sulfate, 3.5 mg folic acid, and a placebo for vitamin A), and (3) iron + folic acid + vitamin A. Control: placebo (a placebo for vitamin A and for iron and folic acid) Setting: community‐level (garment factories in Dhaka City) Timing of intervention: preconception Moderators delivering: field staff, trained nurses
**Outcomes**	Primary: anaemia, iron deficiency, vitamin A deficiencySecondary: compliance
**Notes**	enrolled subjects were followed at weekly intervals for 12 weeks. Funding: Supported by a grant from the Department for International Development, United Kingdom. Beximco Pharmaceutical Co (Dhaka, Bangladesh) provided the iron and folate preparation and the Opsonin Pharmaceutical Co (Barisal, Bangladesh) provided the vitamin A preparation. Declaration of interest: NA

Risk of bias table

**Table:** 

Bias	Authors' judgement	Support for judgement
Random sequence generation (selection bias)	Unclear risk	Quote: “Subjects were randomly allocated to one of the study groups.” Comment: method of sequence generation was not described
Allocation concealment (selection bias)	Low risk	Quote: “An independent person coded the preparations and the code was not broken until all data had been entered into the computer” Comment: adequately done
Similar baseline characteristics	Unclear risk	
Similar baseline outcome measurement	Unclear risk	
Blinding of participants and personnel (performance bias)	Low risk	Comment: Participants: were not aware of the treatment; Personnel: were not aware of the treatment;
Blinding of outcome assessment (detection bias)	Low risk	Quote: “An independent person coded the preparations and the code was not broken until all the data had been entered into the computer.” Comment: outcome assessors were aware of treatment but the outcomes are objective
Incomplete outcome data (attrition bias)	High risk	Quote: “Of the 480 women recruited to the study, 289 subjects completed the 12‐week study protocol: 59% of the placebo group, 62% of the iron + folic acid group, 56% of the vitamin Agroup, and 65% of the iron + folic acid + vitamin A group. One hundred ninety‐one subjects were lost to follow‐up for the following reasons: 75% left their job or moved to another factory, 3% became pregnant, 5% refused to give a second blood sample, 12% were absent on the day of blood collection, and 5% did not take the supplements for the full 12‐week supplementation period.” Comment: Incomplete outcome data were not matched across groups.
Prevention of knowledge of allocated intervention	Unclear risk	
Protection against contamination	Unclear risk	
Selective reporting (reporting bias)	Unclear risk	Comment: Outcomes mentioned in the methods were reported on in the results but no protocol was found.
Other bias	Unclear risk	Quote: “There was some variability in the administration of the supplements, depending on the factory management. In some factories, supplements were given before lunch and in some they were given after lunch. Many subjects came to work after having eaten little or no breakfast and ate only a small lunch” Comment: Other biases might be present

Baird et al. (2010)

**Table:** 

**Methods**	Cluster‐randomised controlled trial—Malawi (cRCT) Randomisation was done using Enumeration Areas (EAs) Study conducted between October 2007 to February 2009.
**Participants**	Young women aged 13 years to 22 years who were never married. Baseline surveys were conducted with 3805 girls, 1225 girls in 88 randomly selected EAs were sampled to be part of the CCT program
**Interventions**	Intervention: conditional cash transfer (CCT) as an incentive for school girls and young women to stay or return to school Control: no CCT provided Setting: Enumeration Areas, community/school Timing of intervention: Moderators delivering:
**Outcomes**	Primary: Pregnancy, sexual behaviour (onset of sexual activity, number of sexual partners in the past 12 months, condom use, frequency of sexual activity) Secondary
**Notes**	Funding: Global Development Network, the Bill and Melinda Gates Foundation, 3ie Open Window (Round 2), NBER Africa Project, and the World Bank. Declaration of interest: No conflicts of interests disclosed

Risk of bias table

**Table:** 

Bias	Authors' judgement	Support for judgement
Random sequence generation (selection bias)	Unclear risk	Quote: “Random sample of 179 Enumeration Areas”—Comment: randomisation method not mentioned
Allocation concealment (selection bias)	Unclear risk	Comment: insufficient information to permit judgement
Similar baseline characteristics	Unclear risk	
Similar baseline outcome measurement	Unclear risk	
Blinding of participants and personnel (performance bias)	Low risk	Comment: unable and unnecessary to blind participants due to type of intervention (cash transfer program)
Blinding of outcome assessment (detection bias)	Unclear risk	Comment: insufficient information to make any judgement
Incomplete outcome data (attrition bias)	Low risk	Quote: “the success rate in tracking our respondents in the study sample was more than 93% in the one‐year follow up.” Comment: attrition rate was low and balanced across treatment and control groups
Prevention of knowledge of allocated intervention	Unclear risk	
Protection against contamination	Unclear risk	
Selective reporting (reporting bias)	Unclear risk	Comment: listed outcomes were reported on but there was no protocol available.
Other bias	Unclear risk	Comment: there were baseline differences between dropouts and school girls (dropouts were older, less literate and more likely to have started childbearing)

Baqui (2018)

**Table:** 

**Methods**	Cluster Controlled Before and After Study Study conducted between 2007 and 2013.
**Participants**	*N* = 4504 pregnant women aged 15–35 + (2247 in the intervention arm and 2257 in the control arm)
**Interventions**	Intervention: integrated post‐partum family planning and maternal and newborn health (PPFP‐MNH) interventions. This included 2‐monthly home visits to identify pregnancies; two antenatal home visits and three postnatal home visits on first, third, and seventh days of childbirth; and identification and referral of sick neonates. In the intervention area, PPFP activities sought to build upon existing home visits to include (a) the integration of behavior change communication (BCC) messages on FP into planned antenatal and postpartum home visits (at 30‐32 weeks of pregnancy; 6 days postpartum and 29‐ 35 days postpartum); (b) on‐going distribution of short term contraceptive methods, including pills and condoms and referrals for clinical methods such as IUDs; and (c) additional home visits at 2 or 3 and 4 or 5 months postpartum for a total of 5 postpartum visits. The visits at 4–5 months postpartum were intended to ensure that women were satisfied with their contraceptive method and to assist Lactational Amenorrhea Method (LAM) users to transition from LAM to another modern family planning method, since LAM is no longer effective after 6 months. PPFP activities also included training and establishment of Community Mobilizers (CMs) and voluntary “LAM Ambassadors” who collaboratively conducted group meetings with women of reproductive age, husbands, mothers and mothers‐in‐laws, and key community stakeholders including religious leaders. Control: MNH interventions only. This included 2‐monthly home visits to identify pregnancies; two antenatal home visits and three postnatal home visits on first, third, and seventh days of childbirth; and identification and referral of sick neonates. Setting: community level unions (unions are the lowest administrative units in Bangladesh with an average population of about 25 000 and a health centre known as Health and Family Welfare Center—H&FWC) in two subdistricts in the Sylhet district Timing of intervention: postconceptional to postpartum period Moderators delivering: Trained community health workers, voluntary “LAM Ambassadors
**Outcomes**	Primary: birth spacing, preterm births Secondary: newborn care practices and survival status of the index child; program exposure (visits by CHWs and CMs), attendance at community meetings, contraceptive use history, subsequent pregnancy Incidences, resumption of menstrual period, sexual activity resumption, and breastfeeding.
**Notes**	“In the case of stillbirths or neonatal deaths, women were interviewed with a shorter form without any reference to postpartum contraceptive use; these women were excluded from the analysis.” Funding: US Agency for International Development Declaration of interest: declared no conflict of interest.

Risk of bias table

**Table:** 

**Bias**	**Authors' judgement**	**Support for judgement**
Random sequence generation (selection bias)	Unclear risk	
Allocation concealment (selection bias)	Unclear risk	Comment: insufficient information to permit judgement.
Similar baseline characteristics	High risk	Quote: “The baseline sample characteristics were similar in terms of women's age, husbands' education, parity and religion (Table 1). However, women in the intervention arm had higher mean years of education (4.5 vs. 4.1 years) and better household economic status compared to women in the control arm.” Comment: Baseline characteristics were different.
Similar baseline outcome measurement	Low risk	Quote: “At baseline, short birth intervals of <24 months and preterm birth rates were similar among women in the intervention and control arms.” Comment: Baseline outcomes were similar
Blinding of participants and personnel (performance bias)	Unclear risk	
Blinding of outcome assessment (detection bias)	Unclear risk	
Incomplete outcome data (attrition bias)	Low risk	Comment: Attrition in the intervention group was 5.8% and 6.0% in the control group.
Prevention of knowledge of allocated intervention	Unclear risk	Comment: insufficient information to permit judgement.
Protection against contamination	Low risk	Comment: Allocation was by unions.
Selective reporting (reporting bias)	Unclear risk	Comment: the outcome measures were reported on but no protocol was found
Other bias	Unclear risk	Quote: “The preterm status was determined based on mothers' reported last menstrual period (LMP), which may be subject to recall error leading to misclassifications. However, we conducted 2‐montly home visits and prospectively collected LMP data from all women. Therefore, the recall period was short and any potential misclassification of term and preterm status should be minimal.” Comment: Possible recall error

Berry (1999)

**Table:** 

**Methods**	Community‐based intervention, China Study cohort were from women identified in the pregnancy monitoring system (October 1993 ‐ September 1995) and who were pregnant at some point between October 1993 ‐ December 31 1996.
**Participants**	247 831, Women preparing for marriage who underwent a physical examination and were later registered with a monitoring system that records prenatal care and delivery
**Interventions**	Intervention: Daily supplement containing 400 mg folic acid. Divided the women who took folic acid pills according to the pattern of use on the basis of the dates they started and stopped taking folic acid. Control: no control, just comparison group Setting: Community level, public health campaign Timing of intervention: periconceptional Moderators delivering:Village health workers recorded the dates that the women started and stopped taking folic acid.
**Outcomes**	Primary: NTD, pregnancy outcome, pattern of use of folic acid pills
**Notes**	Funding: NADeclaration of interest: NA

Risk of bias table

**Table:** 

Bias	Authors' judgement	Support for judgement
Random sequence generation (selection bias)	Unclear risk	
Allocation concealment (selection bias)	Low risk	Quote: “As part of a public health campaign conducted from 1993 to 1995 in an area of China with high rates of neural‐tube defects (the northern region)and one with low rates (the southern region)” Comment: unit of allocation was by province
Similar baseline characteristics	High risk	Comment: no baseline outcome measurements were collected
Similar baseline outcome measurement	High risk	Quote: “In both regions, the women who took folic acid pills were approximately two years younger than those who did not, were more likely to have registered before their last menstrual period, and were more likely to be pregnant for the first time” Comment: baseline characteristics were not similar
Blinding of participants and personnel (performance bias)	Unclear risk	
Blinding of outcome assessment (detection bias)	Unclear risk	
Incomplete outcome data (attrition bias)	Low risk	Quote: “After the exclusion of pregnant women who were lost to follow‐up or those for whom the status of the fetus or infant with respect to a neural‐tube defect was unknown, there were 247,831 pregnant women, 31,960 in the northern region and 215,871 in the southern region (88 percent and 90 percent of all pregnant women, respectively) for whom the neural‐tube–defect status of their fetus or infant was known.” Comment: lost to follow‐up was balanced across groups and an explanation was provided
Prevention of knowledge of allocated intervention	Low risk	Quote: “Village health workers recorded the dates that the women started and stopped taking folic acid.''Three paediatricians, who were unaware of the women's pill‐taking status, independently reviewed the reports and photographs and assigned codes, and a clinical geneticist validated the diagnoses.” Comment: the data collectors were different to the assessors
Protection against contamination	Low risk	Comment: unit of allocation was by province
Selective reporting (reporting bias)	Unclear risk	Comment: there is no evidence that outcomes were selectively reported and protocol was not found.
Other bias	Low risk	Comment: study appears free of other bias

Cabezón (2005)

**Table:** 

**Methods**	Cluster‐randomised controlled trial in Chile. Classrooms were randomised by blindly, taking letters of the class from a bag (simple balloting) 1997 and 1998 cohorts, study dates not reported.
**Participants**	*N* = 1259, 9th grade female students in San Bernardo, Chile, aged 15 years to 16 years, White Hispanic, who had initiated high school in 1997 and 1998
**Interventions**	Intervention: one 45‐ minute class per week for a year on health education, contraceptive education, skills‐ building and abstinence Control: no intervention Setting: Santiago high school Timing of intervention: Moderators delivering: Teachers assigned as monitors were regular teachers of any area, related or not to sex education or biology course.
**Outcomes**	Primary: Unintended pregnancy (ended in term or preterm deliveries or in spontaneous abortion) Secondary
**Notes**	Chile was an upper middle income country at the time this study took place Funding: NA Declaration of interest: NA

Risk of bias table

**Table:** 

Bias	Authors' judgement	Support for judgement
Random sequence generation (selection bias)	Low risk	Quote: “school administration distributes them among 10 classes of 30 to 35 girls each, without previously knowing them. This distribution is random. Among these 10 classes, five of them were alternately selected, as intervention and control groups.” “These eight classes were chosen blindly, taking the letter of the class from a bag” Comment: simple balloting (blindly taking letters from a bag) was used as a randomisation method
Allocation concealment (selection bias)	Unclear risk	Comment: insufficient information to permit judgement
Similar baseline characteristics	Unclear risk	
Similar baseline outcome measurement	Unclear risk	
Blinding of participants and personnel (performance bias)	Low risk	Comment: not possible to blind participants (students and teachers/monitors) due to intervention being sexuality education and the intervention and control groups came from the same school.
Blinding of outcome assessment (detection bias)	Unclear risk	Comment: insufficient information to inform a judgement
Incomplete outcome data (attrition bias)	Low risk	Quote: “Dropout rates were also similar when comparing the intervention with the control groups for the 1997 cohort (22.9% vs. 21.1%) and the 1998 cohort (16.5% vs. 14.5%). Finally it must be stressed that causes of dropout were also comparable, being related mainly to change of residence and financial problems.” Comment: per‐ protocol analysis was carried out but missing outcome data balanced in numbers across intervention groups with similar reasons for missing data across groups (change of residence and financial problems)
Prevention of knowledge of allocated intervention	Unclear risk	
Protection against contamination	Unclear risk	
Selective reporting (reporting bias)	Unclear risk	Comment: the stated outcomes were measured but there was no protocol available.
Other bias	Low risk	Comment: the study appears to be free from other bias

Cowan (2010)

**Table:** 

**Methods**	A cluster randomised controlled trial Study conducted between 2003 and 2007
**Participants**	30 communities, with 15 in the intervention arm and 15 in the comparison arm, in seven districts of Zimbabwe were randomized. A community had three components, the area and population it contained, clinics and secondary schools. The participants consisted of Form two students. Their median age was 15 years at baseline and ranged from 18 to 22 years at follow up time after four years.
**Interventions**	Intervention: The Regai Dzive Shiri intervention consisted of an in‐school teaching program, training of nurses and raising awareness and improving communication in the community with regards to HIV prevention. Control: delayed implementation Setting: Community, schools and clinics Timing of intervention: Preconception Moderators delivering: Research staff, nurses, school leavers
**Outcomes**	Primary: HIV, herpes simplex virus 2 (HSV), pregnancy, knowledge, attitudes Secondary
**Notes**	Funding: The trial was funded by the National Institute of Mental Health (RO1 MH‐66570 PI Cowan Frances Mary). Additional funding was received from DfID Zimbabwe. Declaration of interest: NA

Risk of bias table

**Table:** 

**Bias**	**Authors' judgement**	**Support for judgement**
Random sequence generation (selection bias)	Unclear risk	Quote: “The process of randomisation was as follows. The communities were placed into three strata based on the distance of the clinic from a tarred road. There were 16 299 360 ways that the 30 communities could be allocated to the two arms ensuring balance across these strata. Randomisation was then restricted to ensure an equal number of schools in each arm; balance across districts; and an average sample size per community between 255 and 261 in each arm. The 8575 allocations satisfying these conditions were listed, and one was selected randomly at the randomisation meeting.”—Cogan 2008 Comment: insufficient information to permit judgement
Allocation concealment (selection bias)	Low risk	Comment: intervention and comparison sites were different
Similar baseline characteristics	Unclear risk	
Similar baseline outcome measurement	Unclear risk	
Blinding of participants and personnel (performance bias)	Low risk	Comment: participants could not be blinded due to the nature of the intervention
Blinding of outcome assessment (detection bias)	Unclear risk	Comment: insufficient information to permit judgement
Incomplete outcome data (attrition bias)	High risk	Comment: high amount of loss to follow up from both intervention and control groups
Prevention of knowledge of allocated intervention	Unclear risk	
Protection against contamination	Unclear risk	
Selective reporting (reporting bias)	Unclear risk	Comment: pre‐specified outcomes in the 2008 paper by the same author, were reported; but no protocol was found.
Other bias	Low risk	Comment: the study appears to be free from other bias

Daniel (2008)

**Table:** 

**Methods**	Quasi‐randomized and natural experiment in India Study conducted between 2002 and 2004
**Participants**	Random samples of married women younger than 25 with no more than one child were surveyed in 2002–2003, before PRACHAR was implemented (*N* = 1995), and in 2004, 21–27 months after implementation (*N* = 2080).
**Interventions**	Intervention: The PRACHAR Project seeks to increase contraceptive use for delaying and spacing births through communication intervention. During home visits female change agents, newly married women were counselled on delaying first births and on the correct and consistent use of the pill and condoms. Women who were pregnant for the first time were counselled to space their next birth by using contraceptives. They received information on where to obtain health services for contraception, antenatal care, safe delivery, postpartum care and immunization. Control: comparison areas were chosen because their socioeconomic conditions and accessibility were similar to those of the intervention communities. Setting: community level Timing of intervention: periconceptional Moderators delivering:female change agents, male change agents, training officers, government health workers, rural medical practitioners
**Outcomes**	Primary: Contraceptive demand and use, and related attitudes and knowledge (early childbearing) Secondary
**Notes**	“Women in intervention areas had elevated odds of knowing that fertility varies during the menstrual cycle, and of agreeing that early childbirth can be harmful and that contraceptive use is necessary and safe for delaying first births” Funding: NA Declaration of interest: NA

Risk of bias table

**Table:** 

**Bias**	**Authors' judgement**	**Support for judgement**
Random sequence generation (selection bias)	Unclear risk	
Allocation concealment (selection bias)	Unclear risk	Comment: insufficient information to permit judgement
Similar baseline characteristics	Low risk	Quote: “Respondents were generally similar across survey areas in age, number of children, education and caste.” Comment: similar characteristics
Similar baseline outcome measurement	High risk	Quote: “the proportion of respondents who agreed that early childbearing is injurious to a mother's health increased from 12%to 65%in the comparison areas and from 17%to 74% in the intervention areas.” Comment: one baseline outcome was statistically significantly different
Blinding of participants and personnel (performance bias)	Unclear risk	
Blinding of outcome assessment (detection bias)	Unclear risk	
Incomplete outcome data (attrition bias)	Unclear risk	Quote: “The samples were drawn independently, and the sampling techniques were identical, in the baseline and follow‐up surveys.” Comment: baseline and follow‐up participants were different
Prevention of knowledge of allocated intervention	Unclear risk	Comment: insufficient information to permit judgement
Protection against contamination	Low risk	Comment: allocation was by community area
Selective reporting (reporting bias)	Unclear risk	Comment: the outcome measures were reported on but no protocol is available.
Other bias	Low risk	Comment: study appears free from other bias

Diop (2004)

**Table:** 

**Methods**	Quasi‐experimental design based in three urban communities in northern Senegal. Pre‐ and post‐test control group design. Study conducted between 2000 and 2002
**Participants**	Test interventions to improve the reproductive health of youth aged 10‐19. A total of 2,893 adolescents and 1,683 parents were interviewed at baseline, and 2,738 adolescents and 1,409 parents at end line.
**Interventions**	Intervention: communities of Louga and Saint‐Louis served as intervention sites. sensitization on adolescent reproductive health for community and religious leaders, reaching parents through women's groups, and education sessions led by peer educators using a life skills curriculum. Providers and peer educators were trained to offer youth‐friendly services. Trained teachers and peer educators to provide reproductive health information through a reproductive health curriculum tailored to in‐school youth Control: Diourbel served as a control site, and did not receive any of the intervention components. Setting: community‐based intervention, school‐based and clinic based Timing of intervention: Moderators delivering: Providers, peer educators, teachers
**Outcomes**	Primary: Knowledge and attitudes towards reproductive health (puberty, risks related to adolescent sex, contraception & condoms, use of contraception, HIV/AIDS), sexual activity (age at first intercourse) Secondary
**Notes**	Funding: U.S. Agency for International DevelopmenT (USAID) Declaration of interest: NA

Risk of bias table

**Table:** 

Bias	Authors' judgement	Support for judgement
Random sequence generation (selection bias)	Unclear risk	
Allocation concealment (selection bias)	Unclear risk	Comment: insufficient information to permit judgement
Similar baseline characteristics	Low risk	Quote: “From this analysis, the samples from the two surveys were comparable on most socio‐demographic characteristics.” Comment: socio‐demographic characteristics were similar across the three districts (Table 3)
Similar baseline outcome measurement	High risk	Comment: they were not similar across groups
Blinding of participants and personnel (performance bias)	Unclear risk	
Blinding of outcome assessment (detection bias)	Unclear risk	
Incomplete outcome data (attrition bias)	Unclear risk	Comment: it was a two stage cluster design and the sample was different at baseline and endline
Prevention of knowledge of allocated intervention	Unclear risk	Comment: insufficient information to permit judgement
Protection against contamination	High risk	Quote: “The distance between them is great enough to avoid contamination. Louga was site A, Saint‐Louis was site B, and the town of Diourbel, also distant from Saint‐Lois and Louga, was the control site C.” “Louga was not supposed to implement a school‐based intervention; however, given the difficulty in this city of working within the community, peer educators frequently contacted groups of students and conducted group classes outside schools.” Comment: this was not adequately done
Selective reporting (reporting bias)	Unclear risk	Comment: no protocol was available.
Other bias	High risk	Quote: “The end line survey took place during the international World Cup soccer tournament, reducing the chance of finding boys at home, as not all households have a TV. Boys were often visiting friends or relatives in homes that had TV to watch the matches” Comment: risk of measurement bias is present due to time of data collection

Duflo (2015)

**Table:** 

**Methods**	Seven‐year randomised evaluation of two programs in Kenya (cRCT) Study conducted between 2003 and 2010
**Participants**	328 schools in Kenya's Western Province, sample consists of 19,289 students (9,487 girls and 9,802 boys) enrolled in grade 6
**Interventions**	Intervention: education subsidies and HIV prevention education focused on abstinence until marriage. (2) Stand‐Alone Education Subsidy program (83 schools); (3) Stand‐Alone HIV Education program (83 schools); (4) Joint Program (80 schools). Control: Control (82 schools) Setting: school level Timing of intervention: Moderators delivering: second program trained teachers on how to deliver the national HIV/AIDS prevention curriculum to upper primary school students.
**Outcomes**	Primary: STI, teenage pregnancy rate
**Notes**	Funding: the Hewlett Foundation, the MacArthur Foundation, NIH grant R01HD039922, the Nike Foundation, the Partnership for Child Development, and the World Bank. Declaration of interest: no relevant or material financial interests that relate to the research described in this paper

Risk of bias table

**Table:** 

Bias	Authors' judgement	Support for judgement
Random sequence generation (selection bias)	Low risk	Quote: “Schools were stratified and assigned to one of four arms using a random number generator.” Comment: Adequately done
Allocation concealment (selection bias)	Low risk	Comment: unit of allocation was by institution
Similar baseline characteristics	Unclear risk	
Similar baseline outcome measurement	Unclear risk	
Blinding of participants and personnel (performance bias)	Low risk	Comment: not possible to due to type of intervention
Blinding of outcome assessment (detection bias)	Unclear risk	Comment: insufficient information to permit judgement
Incomplete outcome data (attrition bias)	Low risk	Quote: “There is no evidence of differential attrition for any outcome, except for dropout information after five years.”—refer to B. Attrition page 1 Comment: adequately done
Prevention of knowledge of allocated intervention	Unclear risk	
Protection against contamination	Unclear risk	
Selective reporting (reporting bias)	Unclear risk	Comment: No protocol was available.
Other bias	Low risk	Comment: study appears free from other bias

Dupas (2011)

**Table:** 

**Methods**	Quasi experimental study. Randomised experiment in two rural districts of Western Kenya Study conducted between 2003 and 2005
**Participants**	Students (males and females) from 328 primary schools
**Interventions**	Intervention: four groups of schools: (1) teacher training program and relative risk information, (2) only teacher training, (3) only relative risk information, and (4) neither programs Teacher training: 163 schools were randomly selected received teacher training on the national HIV/AIDS curriculum, which focuses on average risk and encourages abstinence until marriage, but does not discuss risk reduction strategies (such as condom use or selection of safer partners). Relative Risk Information Campaign: schools receiving the teacher training also received an information campaign, which provided teenagers (only students in grade 8) with information on the prevalence of HIV disaggregated by age and gender group Control: control schools received neither program Setting: school‐level Timing of intervention: Periconception Moderators delivering: teachers
**Outcomes**	Primary: incidence of unprotected sex between teenage girls and male partners five or more years older, the incidence of unprotected sex between teenage girls and teenage boys (measured by incidence of childbearing)
**Notes**	Funding: World Bank. Declaration of interest: NA

Risk of bias table

**Table:** 

**Bias**	**Authors' judgement**	**Support for judgement**
Random sequence generation (selection bias)	Unclear risk	
Allocation concealment (selection bias)	Low risk	Comment: allocation was done on the school level
Similar baseline characteristics	Low risk	Comment: school characteristics were balanced across groups (Table 3)
Similar baseline outcome measurement	Low risk	Quote: “No comprehensive baseline survey was administered to the study cohort. However, as described in Section IB, students in RR schools were asked to fill a short, anonymous “priors” survey just before the RR information was provided to them.” Comment: the 'priors' survey demonstrated similar measurements across study groups
Blinding of participants and personnel (performance bias)	Unclear risk	
Blinding of outcome assessment (detection bias)	Unclear risk	
Incomplete outcome data (attrition bias)	Unclear risk	Comment: insufficient information to permit judgement
Prevention of knowledge of allocated intervention	Unclear risk	Comment: insufficient information to permit judgement
Protection against contamination	Low risk	Comment: unit of allocation was by school
Selective reporting (reporting bias)	Unclear risk	Comment: no protocol was available.
Other bias	Low risk	Quote: “While overall the self‐reported data in this paper seems consistent with the biological (childbearing) data, it is important to keep in mind that the estimates obtained with that data could suffer from reporting biases.” Comment: bias is unlikely

Erulkar (2007)

**Table:** 

**Methods**	Quasi experimental study. Evaluation of a program to promote education and delay marriage in Rural Ethiopia. Study conducted between 2004 and 2006
**Participants**	The Berhane Hewan program targets married and unmarried girls aged 10 to 19. Endline sample included 464 girls in the control area and 462 in the experimental area.
**Interventions**	Intervention: three components: (1) social mobilization and group formation by adult female mentors, (2) participation in nonformal education and livelihoods training for out of school girls, or support to remain in school, and (3) “community conversations” which involved meetings of all community members and discussions were on early marriage, other harmful traditional practices, and matters affecting young women and girls. Baseline and end line surveys were implemented for both intervention and control areas.Control: Enamirt KA in Mecha District/Woreda served as the control, selected because of its similar socioeconomic profile.Setting: community‐levelTiming of intervention:Moderators delivering: adult female mentors, community conversations facilitators
**Outcomes**	Primary: increased friendship networks, increased access to safe spaces, increased participation, increased school attendance, increased school attainment, increased levels of literacy, decreased proportion of married adolescents, increased knowledge of reproductive health topics, increased discussion of reproductive health topics, increased use of family planning methods (e.g., Condoms)
**Notes**	Funding: UNFPA, the Nike Foundation and United Nations Foundation.Declaration of interest: NA

Risk of bias table

**Table:** 

Bias	Authors' judgement	Support for judgement
Random sequence generation (selection bias)	Unclear risk	
Allocation concealment (selection bias)	Low risk	Comment: kebele administrations were used as the unit of treatment group
Similar baseline characteristics	High risk	Quote: “There were no significant differences between groups at baseline and end line in terms of age, parental co‐residence and school status. At baseline, girls in the experimental sites significantly differed from the controls in terms of marital status, parenthood status and socioeconomic status.” Comment: baseline characteristics were not similar
Similar baseline outcome measurement	High risk	Quote: “At baseline, Mosebo girls were significantly less likely to talk to their closest friends about family planning, condoms, and violence in their communities” Comment: baseline outcomes were not similar
Blinding of participants and personnel (performance bias)	Unclear risk	
Blinding of outcome assessment (detection bias)	Unclear risk	
Incomplete outcome data (attrition bias)	Unclear risk	Comment: there was a greater sample size at end line compared to baseline—therefore incomplete outcome data cannot be determined
Prevention of knowledge of allocated intervention	Unclear risk	Comment: insufficient information to permit judgement
Protection against contamination	Low risk	Quote: “The results indicate that there was no contamination in the control group” Comment: this was adequately done
Selective reporting (reporting bias)	Unclear risk	Comment: no protocol was available.
Other bias	Low risk	Comment: study appears free from other bias

Februhartanty (2002)

**Table:** 

**Methods**	Single blind community experimental study in Indonesia RCT (with a nonrandomised third arm) Study conducted between August to December 1998
**Participants**	*N* = 137 postmenarchal female adolescent students
**Interventions**	Intervention: (1) 48 students got an iron tablet weekly; (2) 41 students took an iron tablet for four consecutive days during their menstruation cycle. Iron tablet included 60 mg elemental iron and 0.25 mg folic acid in the form of 200 mg ferrous sulphate Control: Forty eight students received a placebo tablet weekly Setting: school‐level, community based Timing of intervention: preconception Moderators delivering: teachers
**Outcomes**	Primary: Haemoglobin, serum ferritin, height, weight, mid‐upper arm circumference and dietary intake. Prevalence of anaemia
**Notes**	Funding: SEAMEO‐TROPMED Regional Center for Community Nutrition in Jakarta Declaration of interest: NA

Risk of bias table

**Table:** 

Bias	Authors' judgement	Support for judgement
Random sequence generation (selection bias)	Unclear risk	Quote: “One hundred of them were recruited from one school and allocated randomly to placebo or weekly groups.The other 50 students were recruited at random from a different junior high school and allocated to the menstruation group. This allocation method was chosen for practical reasons to avoid confusion in the field.” Comment: method of sequence generation was not described
Allocation concealment (selection bias)	Unclear risk	Comment: insufficient information to prevent judgement
Similar baseline characteristics	Unclear risk	
Similar baseline outcome measurement	Unclear risk	
Blinding of participants and personnel (performance bias)	Low risk	Quote: “Weekly supplementation was implemented every Wednesday for both placebo and iron groups.” Comment: participants: were not aware of the treatments (use of a placebo) Personnel: not described
Blinding of outcome assessment (detection bias)	Unclear risk	Comment: not described
Incomplete outcome data (attrition bias)	Low risk	Quote: “The final data set consisted of 48 subjects in each of the study groups (4% drop out per group). Common reasons for dropping out were refusal to undergo blood collection,absent from class on the day of blood collection, and transfer to another school” Comment: missing outcome data were matched across groups
Prevention of knowledge of allocated intervention	Unclear risk	
Protection against contamination	Unclear risk	
Selective reporting (reporting bias)	Unclear risk	Quote: “After excluding extreme values (serum ferritin changes of more than 50 μg/L), a complete data set of serum ferritin levels covered 34 subjects in the placebo group and 31 subjects in the weekly group” Comment: complete information not available nor was a protocol available
Other bias	High risk	Quote: “More anaemic subjects were found in the weekly group,with the prevalence of anaemia being 72.9% in this group.” Comment: higher prevalence of anaemia in the weekly group

Gallegos et al. (2008)

**Table:** 

**Methods**	Randomized controlled trial with four follow ups in MexicoStudy conducted from 2002 to 2005
**Participants**	*N* = 832 adolescents recruited from high schools, age 14‐17, (459 girls, 370 boys) were randomly assigned to the experimental or control group.
**Interventions**	Intervention: Two types of intervention: (1) reduction of HIV/AIDs risk, (2) health promotion. Six hour intervention used active learning strategies, and was delivered in two sessions on two consecutive Saturdays. Groups consisted of 6‐8 members. Control: control group was present but limited details are provided Setting: school‐level, community‐level Timing of intervention: Moderators delivering: facilitators (*n* = 31; 6 men, 25 women)
**Outcomes**	Primary: intentions of having sex, intentions to use condoms, intention to use other contraceptives, behavioural beliefs around contraceptives (e.g., use of condoms) Secondary
**Notes**	Funding: the National Institute of Nursing Research (NINR) of the United States Declaration of interest: NA

Risk of bias table

**Table:** 

Bias	Authors' judgement	Support for judgement
Random sequence generation (selection bias)	Unclear risk	Quote: “Participants were assigned a code per family when registering, which was the basis for the random assignment to the experimental or control condition…the adolescents were stratified by gender and age, assigning them randomly, now to groups of 6‐8 members.” Comment: method of randomization not explained
Allocation concealment (selection bias)	Unclear risk	Comment: insufficient information to permit judgement
Similar baseline characteristics	Unclear risk	
Similar baseline outcome measurement	Unclear risk	
Blinding of participants and personnel (performance bias)	Low risk	Comment: blinding of participants not possible due to nature of intervention
Blinding of outcome assessment (detection bias)	Unclear risk	Comment: insufficient information to permit judgement
Incomplete outcome data (attrition bias)	Low risk	Quote: “About 94.7% of the sample attended the three‐month follow‐up, 94.6% attended the six‐month follow‐up, and 90.6% attended the 12‐month follow‐up. According to the logistic regression analysis (GEE), there was no significant difference in the percentages of attendance at the follow‐up sessions for data collection, between the experimental and control groups. Additional analysis indicated that the variables gender, age and sexual experience were not related to the probability of dropping out of the study.” Comment: outcome data balanced across groups
Prevention of knowledge of allocated intervention	Unclear risk	
Protection against contamination	Unclear risk	
Selective reporting (reporting bias)	Unclear risk	Comment: insufficient information to permit judgement and no protocol was found
Other bias	Low risk	Comment: the study appears free from other bias

Gilgen (2001)

**Table:** 

**Methods**	A randomised double‐blind intervention trial in a tea estate in Bangladesh Study (field work) took place between November 1995 and March 1997
**Participants**	*N* = 280 adult, female tea pluckers, who were not pregnant and not breast feeding
**Interventions**	Intervention: received weekly iron supplementation (200 mg ferrous fumarate and 200 mg folic acid) for 24 weeks (*n* = 139) Control: placebo (*n* = 141), manufactured by the same company. Setting: community level Timing of intervention: preconception Moderators delivering: NA
**Outcomes**	Primary: haemoglobin, hematocrit and ferritin concentration. Mean corpuscular haemoglobin concentration helminth infections (prevalence and intensity); anaemia; improvement in well being (stronger, more energetic, relief from stomach pains, anorexia and diarrhoea); adverse effects
**Notes**	Funding: Unicef and The Nestle Foundation. Declaration of interest: NA

Risk of bias table

**Table:** 

Bias	Authors' judgement	Support for judgement
Random sequence generation (selection bias)	Low risk	Quote: “The random number generator in SPSS (Version 7.5) was used to create four groups of equal size and the process was repeated until there was no statistically significant difference between the randomised groups in mean age,years of plucking experience, productivity of the previous plucking season, haemoglobin and ferritin values and prevalence and egg counts of *Ascaris, Trichuris* and hookworms.” Comment: adequately done
Allocation concealment (selection bias)	Unclear risk	Comment: insufficient information to permit judgement
Similar baseline characteristics	Unclear risk	
Similar baseline outcome measurement	Unclear risk	
Blinding of participants and personnel (performance bias)	Low risk	Comment: participants: were not aware of the treatments; personnel: were not aware of the treatments
Blinding of outcome assessment (detection bias)	Unclear risk	Outcome assessors: not described
Incomplete outcome data (attrition bias)	Unclear risk	Comment: insufficient information to permit judgement
Prevention of knowledge of allocated intervention	Unclear risk	
Protection against contamination	Unclear risk	
Selective reporting (reporting bias)	Unclear risk	Comment: outcomes mentioned in the introduction were reported on in the results but no protocol was available
Other bias	Low risk	Comment: there is no evidence of other bias

Hall et al. (2002)

**Table:** 

**Methods**	A randomised controlled trial in Mali Study conducted from January 2000 to 14‐16 weeks later
**Participants**	552 schoolgirls
**Interventions**	Intervention:IF weekly (single tablet 300 mg ferrous sulphate weekly for 10 weeks): 30 schools (271 girls). For 10 weeks a tablet providing 65 mg of iron and 0.25 mg of folic acid Control: 30 schools no iron tablets were given (*n* = 281) Setting: school‐level (rural community schools) Timing of intervention: preconception Moderators delivering: teachers
**Outcomes**	Primary: haemoglobin concentration, prevalence of anaemia, adherence Secondary
**Notes**	Funding: Save the Children, USA and Helen Keller International, Mali. Declaration of interest: NA

Risk of bias table

**Table:** 

Bias	Authors' judgement	Support for judgement
Random sequence generation (selection bias)	Low risk	Quote: “60 schools were then randomly assigned to either a treatment or a comparison group.” Comment: a computer‐generated random number list (information communicated by the author)
Allocation concealment (selection bias)	Low risk	Comment: not reported. Since the intervention was allocated at school level, it is unlikely there was a selection bias at the individual level
Similar baseline characteristics	Unclear risk	
Similar baseline outcome measurement	Unclear risk	
Blinding of participants and personnel (performance bias)	Unclear risk	Comment: insufficient information to permit judgement
Blinding of outcome assessment (detection bias)	Unclear risk	Comment: insufficient information to permit judgement.
Incomplete outcome data (attrition bias)	Low risk	Quote: “1201 children at the baseline survey and 1113 children 14–16 weeks later. The haemoglobin concentration of the 88 children who dropped out of the study or did not provide a second blood sample was not significantly different at baseline from those children who remained in the study.” Comment: missing outcome data were matched across groups
Prevention of knowledge of allocated intervention	Unclear risk	
Protection against contamination	Unclear risk	
Selective reporting (reporting bias)	Unclear risk	Comment: outcomes described in the methods were reported in the results section but there was no protocol available
Other bias	Low risk	Comment: the study appears to be free of other bias

Handa et al. (2015)

**Table:** 

**Methods**	Clustered randomised controlled trial (longitudinal design) to evaluate the impact of the Kenya Cash Transfer Study conducted from March 2007 to 2011
**Participants**	females aged 12 to 24 (n = 1549), randomised by households
**Interventions**	Intervention: provides a monthly cash sum to eligible households, beneficiaries were told that they were expected to use the money for the care and development of the OVC resident in the household. Control: existence of a control group, which was delayed entry to the program due to budget constraints. Setting: community‐level, households Timing of intervention: Moderators delivering: Location OVC (orphans and vulnerable children)Committees (LOCs)
**Outcomes**	Primary: ever been pregnant, ever married or co‐habiting, likelihood of pregnancy, likelihood of early marriage, initiation of sexual intercourse (delay in sexual debut) Secondary: schooling (enrolment), in‐migration between households
**Notes**	In the 2011 third‐wave of the survey, a fertility module was introduced, which asked all female residents age 12‐49 a series of questions regarding pregnancy, health behaviour around birth, and other fertility history. Funding: U.S. National Institute of Mental Health through Grant Number 1R01MH093241 and by Eunice Kennedy Shriver National Institute of Child Health and Development R24 HD050924 to the Carolina Population Center. Declaration of interest: NA

Risk of bias table

**Table:** 

Bias	Authors' judgement	Support for judgement
Random sequence generation (selection bias)	Low risk	Quote: “two Locations in each district were randomly chosen by lottery to serve as delayed entry controls and the program was implemented in the other two” “It contained 1542 and 755 treatment and control households respectively (a ratio of 2:1), which were randomly selected via computer generated ranking in each Location” Comment: adequately done
Allocation concealment (selection bias)	Low risk	Quote: “survey teams and households did not know their treatment status to avoid biases in responses and survey enumeration” Comment: adequately done
Similar baseline characteristics	Unclear risk	
Similar baseline outcome measurement	Unclear risk	
Blinding of participants and personnel (performance bias)	Low risk	Quote: “survey teams and households did not know their treatment status to avoid biases” Comment: Adequately done
Blinding of outcome assessment (detection bias)	Unclear risk	Comment: insufficient information to permit judgement
Incomplete outcome data (attrition bias)	Low risk	Quote: “Although attrition levels at the household level were high between the baseline and first follow‐up in 2009 (18 percent), corresponding with the political turmoil and election violence, we examine differential attrition at the household and individual level and find little evidence of differential attrition” Comment: effect of attrition is accounted for
Prevention of knowledge of allocated intervention	Unclear risk	
Protection against contamination	Unclear risk	
Selective reporting (reporting bias)	Unclear risk	Quote: “analyses impacts on early pregnancy and marriage among females aged 12 to 24 at the four‐year follow‐up in 2011.” Comment: the outcomes stated in the aim were reported on but no protocol was available
Other bias	Low risk	Comment: study appears free from other bias

James et al. (2005)

**Table:** 

**Methods**	Quasi experimental study. A pre‐post test follow‐up design in South Africa Study dates not reported
**Participants**	*N* = 1168 secondary school learners. Total of 19 schools in the final sample, equally distributed in rural and urban areas. Age: 15 to >22 years
**Interventions**	Intervention: did read Laduma (*n* = 9), provides accurate factual information to increase students' knowledge and reduce misperceptions about STIs, exposes them to real life situations, possible risks and solutions. Also it aims to engender a positive attitude towards safe sexual practices and to enhance self‐efficacy and adoption of skills, such as talking about sexually transmitted infections and prevention with partners, negotiating safer sexual practices and decision‐making about seeking help. Control: randomly allocated to control (did not read Laduma) (*n* = 10) Setting: school‐level Timing of intervention: Moderators delivering: The questionnaire was individually filled in by learners and was supervised by a fieldworker and researcher during a normal class lesson.
**Outcomes**	Primary: knowledge on STIs, attitude to condom use, towards people with STIs and/or HIV/AIDS, intention to practice safe sex, intention to practice safe sex, communication about STIs
**Notes**	Funding: NACOSA Declaration of interest: NA

Risk of bias table

**Table:** 

Bias	Authors' judgement	Support for judgement
Random sequence generation (selection bias)	Unclear risk	
Allocation concealment (selection bias)	Low risk	Quote: “The schools were randomly allocated to control (did not read Laduma) and intervention (did read Laduma) groups. There were 10 control and nine intervention schools” Comment: unit of allocation was done by school, however the method of allocation was not discussed
Similar baseline characteristics	High risk	Quote: “Tests of independence revealed that both the distribution of boys and girls and the distribution of first language (English versus Zulu) differed for the intervention and control group.” Comment: baseline characters were not similar
Similar baseline outcome measurement	Low risk	Quote: “…knowledge about spread of sexually transmitted infections revealed that the knowledge levels of the intervention group and the control group did not differ at baseline.' '… we found no difference between the intervention and control group at baseline.” Comment: no important differences between baseline outcomes were observed
Blinding of participants and personnel (performance bias)	Unclear risk	
Blinding of outcome assessment (detection bias)	Unclear risk	
Incomplete outcome data (attrition bias)	Low risk	Quote: “No difference in drop out between intervention group and the control group was found.” Comment: this was adequately addressed
Prevention of knowledge of allocated intervention	Unclear risk	Comment: insufficient information to permit judgement
Protection against contamination	Low risk	Comment: allocation was done via institution
Selective reporting (reporting bias)	Unclear risk	Comment: insufficient information to permit judgement and no protocol was found
Other bias	Low risk	Comment: study appears free from other bias

Jewkes et al. (2006)

**Table:** 

**Methods**	Cluster randomised‐controlled trial in rural Eastern Cape, South Africa Study dates not reported.
**Participants**	A total of 2776 participants (1409 intervention and 1367 control) were enrolled at baseline and had an interview. The intervention was implemented in 35 communities in two workshops of 20 men and 20 women in each community. Aged 16–23 years,
**Interventions**	Intervention: Stepping Stones is an approach to HIV prevention that aims to improve sexual health through building stronger, more gender‐equitable relationships with better communication between partners. The issues covered in the 13 sessions include reflecting on love, sexual health joys and problems, body mapping, menstruation, contraception and conception (including infertility), sexual problems, unwanted pregnancy, HIV, STDs, safer sex, gender‐based violence, motivations for sexual behaviour, and dealing with grief and loss. A total of 17 sessions (50 h) over a period of 3–12 weeks (including core sessions, peer groups, and a final community meeting) Control: control arm communities attended a single session of about 3 h on HIV and safer sex. Setting: community level Timing of intervention: Moderators delivering: trained, gender matched facilitator (young men and women who were slightly older than the study participants.)
**Outcomes**	Primary: HIV incidence Secondary: changes in knowledge, attitude and sexual behaviours
**Notes**	Knowledge and attitudes about STDs, HIV, condom use Funding: National Institute of Mental Health grant number MH 64882‐01 and the South African Medical Research Council. Declaration of interest: NA

Risk of bias table

**Table:** 

Bias	Authors' judgement	Support for judgement
Random sequence generation (selection bias)	Low risk	Quote: “The study statistician (JL) based in Pretoria, who had no knowledge of the study area, randomly generated the allocation sequence for each stratum. Then enrolled participants.” Comment: random sequence generation was adequately done
Allocation concealment (selection bias)	Low risk	Quote: “The study statistician (JL) based in Pretoria, who had no knowledge of the study area, randomly generated the allocation sequence” Comment: allocation was adequately concealed
Similar baseline characteristics	Unclear risk	
Similar baseline outcome measurement	Unclear risk	
Blinding of participants and personnel (performance bias)	Low risk	Comment: personnel (field workers and interviewers) could not be blinded and were used for quality control of the measures. There was active recruiting the villages for participants in the study, again given the nature of the intervention blinding was not possible.
Blinding of outcome assessment (detection bias)	Low risk	Quote: “All blood tests were conducted blind to the treatment arm” Comment: outcome assessment was blind
Incomplete outcome data (attrition bias)	Low risk	Quote: “No clusters were lost to follow‐up.” “Loss to follow‐up was mainly because participants had moved and could not be located.” Comment: missing outcome data is balanced across groups
Prevention of knowledge of allocated intervention	Unclear risk	
Protection against contamination	Unclear risk	
Selective reporting (reporting bias)	Unclear risk	Comment: insufficient information to permit judgement and no protocol was found.
Other bias	Low risk	Comment: no evidence to suggest this

Kaljee et al. (2005)

**Table:** 

**Methods**	Randomized–controlled effectiveness trial in Vietnam (cRCT) Study conducted from September 2001 to July 2003
**Participants**	*N* = 480 adolescents (15‐20 years) were randomised into control and intervention groups. Convenience sample of 60 youth was selected from each of the eight study communes, for a total of 480 participants.
**Interventions**	Intervention: The Vietnamese Focus on Kids program includes eight sessions and two sessions for community project development and delivery. Designed to teach youth new skills for decision making and communication, as well as factual information about HIV/AIDS and other STIs, birth control, and condom use. The program was delivered once a week for 10 consecutive weeks, each session was approximately 2 h, each commune there were six groups of 10 same–gender youth, with one facilitator per group. Control: control youth received the intervention after collection of the 18–month follow–up data (at conclusion of study) Setting: school‐level (program delivery sites were the local schools) Timing of intervention: Moderators delivering: The facilitators included teachers, youth leaders, and commune centre health care providers.
**Outcomes**	Primary: self‐efficacy (condom use), response efficacy (belief that condoms will reduce risk to STIs/AIDs and unplanned pregnancy) Secondary
**Notes**	Beliefs = attitudes Eg. Sex difference reported in text: “A total of 15/480 youth (3.1%) report engaging in “other” sex, with 11/15 of those respondents female” “intention to engage in sexual intercourse, there was no significant difference between control and intervention groups at either post‐intervention”—not separated by gender Funding: Fogarty International AIDSFIRCA Declaration of interest: NA

Risk of bias table

**Table:** 

Bias	Authors' judgement	Support for judgement
Random sequence generation (selection bias)	Unclear risk	Quote: “Randomization was at the commune level” Comment: randomisation method was not mentioned in the text
Allocation concealment (selection bias)	Unclear risk	Comment: insufficient information to permit judgement
Similar baseline characteristics	Unclear risk	
Similar baseline outcome measurement	Unclear risk	
Blinding of participants and personnel (performance bias)	Low risk	Comment: this is not possible with this type of intervention
Blinding of outcome assessment (detection bias)	Unclear risk	Comment: insufficient information to permit judgement
Incomplete outcome data (attrition bias)	Low risk	Quote: “Participant retention rates remained high throughout the study with 466/480 (97.1%) of youth completing the postintervention and 454/480 (94.6%) completing the 6–month follow–up evaluation.” Comment: this issue was adequately addressed
Prevention of knowledge of allocated intervention	Unclear risk	
Protection against contamination	Unclear risk	
Selective reporting (reporting bias)	Unclear risk	Comment: there was no accessible protocol
Other bias	Low risk	Comment: there is no evidence to suggest any kind of bias

Kanani (2000)

**Table:** 

**Methods**	An experimental placebo control study in Vadodora, IndiaQuasi‐randomised trial Study date not reported
**Participants**	*N* = 210 unmarried adolescent girls 10–18 y of age.
**Interventions**	Intervention: Girls in the experimental group received iron folic acid tablets for 3 months (60 mg of elemental iron + 0.5 mg folic acid per day) (*n* = 101) Control: the control group received a similar‐looking placebo tablet accordingly (*n* = 102)Setting: community‐level Timing of intervention: Moderators delivering: NA
**Outcomes**	Primary: haemoglobin, hunger score, weight, BMI Secondary: compliance
**Notes**	Funding: NADeclaration of interest: NA

Risk of bias table

**Table:** 

Bias	Authors' judgement	Support for judgement
Random sequence generation (selection bias)	Unclear risk	
Allocation concealment (selection bias)	Unclear risk	Comment: allocation was by community, however allocation was selected by the authors
Similar baseline characteristics	Low risk	Quote: “Similarly, baseline Hb and BMI did not differ.” Comment: baseline outcomes were similar
Similar baseline outcome measurement	Low risk	Quote: “Baseline socioeconomic status, housing conditions, water supply and sanitation, and health facilities were similar in both communities.” Comment: baseline characteristics were similar
Blinding of participants and personnel (performance bias)	Unclear risk	
Blinding of outcome assessment (detection bias)	Unclear risk	
Incomplete outcome data (attrition bias)	Unclear risk	Comment: insufficient information to permit judgement
Prevention of knowledge of allocated intervention	Low risk	Comment: most of the primary outcomes were objective except hunger which was self‐reported and therefore would not be influenced by assessor bias
Protection against contamination	Low risk	Quote: “the larger community became the experimental group and the two smaller ones became the control group.” Comment: allocation was by community
Selective reporting (reporting bias)	Unclear risk	Comment: insufficient information to permit judgement; and no protocol was found
Other bias	Low risk	Comment: study appears free from other bias

Kanesathasan et al. (2008)

**Table:** 

**Methods**	Quasi‐experimental study design to evaluate the impact of a program (DISHA) in India Study conducted from September 2004 to October 2007.
**Participants**	Conducted baseline, cross‐sectional, household surveys among married and unmarried, male and female youth, ages 14‐24 years (*n* = 4645), and among adults aged 30 years and older (n = 1601) living in all six of the NGO catchment areas.
**Interventions**	Intervention: (1) mass or generalized: limited engagement with youth (2) targeted or individualized: youth groups (provided young people with health information on a range of topics, including adolescence, gender and sexuality, fertility awareness, contraception, HIV and AIDS, safe motherhood, and reproductive health services), peer education (volunteer peer educators ‐ married and unmarried males and females ‐ to provide information, counselling, support and referrals to their peers through youth groups and individual sessions.), livelihoods (income generating opportunities and skills), 36 focus group discussions, conducted after the end line surveys, were used to contextualise quantitative findings regarding changes in reproductive health knowledge, attitudes and behaviours; provide insights and feedback on DISHA program interventions Control: mentions use of control groups (control sites) Setting: community, clinics Timing of intervention: Moderators delivering: peer educators, youth depot holders and health service providers
**Outcomes**	Primary: youth level outcomes: knowledge and attitudes (marriage, of all six modern contraceptive methods—including condoms, use of contraceptives and reproductive health services), youth empowerment (self‐efficacy, communication with elders, spousal communication and mobility), livelihoods engagement, youth behaviours (age at marriage, contraceptive prevalence and use of sexual and reproductive health services among youth) Secondary: community level outcomes
**Notes**	Other aspects of program: community support for youth sexual and reproductive health needs (community entry & mobilisation, adult groups and youth‐adult partnership groups), youth‐friendly reproductive health services (Youth‐friendly health services, Youth contraceptive depot holders), and building NGO Capacity for Sustainability One of DISHA objectives: delay marriage and childbearing among youth and strengthen their ability to make informed decisions about reproductive health matters, especially among females Funding: David and Lucile Packard Foundation Declaration of interest: NA

Risk of bias table

**Table:** 

Bias	Authors' judgement	Support for judgement
Random sequence generation (selection bias)	Unclear risk	
Allocation concealment (selection bias)	Unclear risk	Quote: “…designed, implemented and evaluated DISHA in two Indian states: Bihar and Jharkhand.” Comment: the program evaluation mentions the use of a control group but whether these groups were villages in these states or outside is unclear
Similar baseline characteristics	Unclear risk	Comment: insufficient information to permit judgement
Similar baseline outcome measurement	Unclear risk	Comment: there were differences between participant outcomes between the two states at baseline—however both these outcomes were reported for the intervention, with minimal information on the control cohort's baseline outcomes.
Blinding of participants and personnel (performance bias)	Unclear risk	
Blinding of outcome assessment (detection bias)	Unclear risk	
Incomplete outcome data (attrition bias)	Unclear risk	Comment: greater sample size following the intervention (at end line)
Prevention of knowledge of allocated intervention	Unclear risk	Comment: insufficient information to permit judgement
Protection against contamination	Unclear risk	Comment: it is unclear whether the control sites were within the same states/villages as the intervention sites, which may have led to contamination
Selective reporting (reporting bias)	Unclear risk	Comment: insufficient information to permit judgement; and no protocol was found
Other bias	Low risk	Comment: study seems free from other bias

Klepp et al. (1997)

**Table:** 

**Methods**	Quasi randomised study (A randomised‐controlled community trial in Tanzania) Study conducted between 1992 and 1993
**Participants**	*N* = 1063 sixth graders participated in baseline and 814 in follow‐up Public primary schools randomly assigned to intervention (*n* = 6) or control (*n* = 12)
**Interventions**	Intervention: Ngao is a local HIV/AIDS education program. Activities included: (1) Teachers provided factual information about HIV transmission and AIDS.(2) Students created their own posters depicting their perceptions of HIV risk factors.(3) Students wrote and performed songs and poetry about the danger of AIDS and how children their own age can protect themselves. (4) Students working in small groups discussed how people are exposed to HIV risk and what they themselves could do to reduce such risk. (5) Students wrote and performed role‐plays in which they argued publicly, trying to convince each other about aspects of HIV risk behaviours or practicing refusal skills relating to sexual involvement. (6) Students created and performed elaborate plays in which they wore their traditional clothes instead of school uniforms. These plays portrayed how AIDS was perceived and could be dealt with in the community. (7) Students performed the plays, role‐plays, poetry, and songs outdoors in front of younger schoolmates. In this way, the program also had the potential to educate younger children. Other activities were designed to increase communication with parents and other community members on AIDS issues. Control: delayed‐intervention comparison group. Setting: School based Timing of intervention: Moderators delivering: Trained Tanzanian project staff, teachers, local health workers
**Outcomes**	Primary: Knowledge, sexual initiation Behavioural intention to engage in sexual intercourse over the next 3 months was measured by one item. Ever had sex
**Notes**	Funding: the Norwegian Agency for Development Cooperation and by the Tanzanian Ministry of Health.Declaration of interest: NA

Risk of bias table

**Table:** 

Bias	Authors' judgement	Support for judgement
Random sequence generation (selection bias)	Unclear risk	
Allocation concealment (selection bias)	Unclear risk	Comment: Insufficient information to permit a judgement
Similar baseline characteristics	Low risk	Quote: “As can be seen in Table 1, there were no significant differences between students from intervention schools and comparison schools on any of the socio‐demographic characteristics.” Comment: baseline characteristics were similar
Similar baseline outcome measurement	Low risk	Quote: “There were no statistically significant differences between students from intervention schools and comparison schools on any of the HIV/AIDS‐related outcome measures at baseline.” Comment: baseline outcomes were similar
Blinding of participants and personnel (performance bias)	Unclear risk	
Blinding of outcome assessment (detection bias)	Unclear risk	
Incomplete outcome data (attrition bias)	Low risk	Quote: “attrition rate was higher in the comparison schools than in the intervention schools (25.1% vs. 19.6%)' 'Furthermore, students who dropped out of the study had been found at baseline to have less exposure to AIDS information (8.4 vs. 9.2; P = 0.02) and to hold subjective norms more favourable toward becoming sexually active' we do not believe that this attrition was due to students not wanting to participate in the study; rather, we believe that this attrition reflects the high proportion of school dropouts in the Tanzanian public school system.” Comment: this was adequately done
Prevention of knowledge of allocated intervention	Unclear risk	Comment: insufficient information to permit judgement
Protection against contamination	Low risk	Comment: contamination unlikely as the intervention did not take place in the same schools as the comparison
Selective reporting (reporting bias)	Unclear risk	Comment: insufficient information to permit judgement; and no protocol was found
Other bias	Low risk	Comment: no evidence to suggest this

*Li* 2014

**Table:** 

**Methods**	Parallel group design study, China Study date not reported
**Participants**	women of childbearing age (19‐43 years old) inTongliao city in the NeiMonggol Autonomous Region ofNorth China.4052 women in China undergoing prenatal consultations were divided into 2 ethnic groups, Han or Mongolian, and were further assigned into 1 of 4 groups
**Interventions**	Intervention: group 1 received folic acid tablets before pregnancy and drank liquid milk (containing folate which ranged in concentration from 1.68 to 5.69 μg/100 ml); group 2 did not take folic acid tablets before pregnancy but drank milk daily once pregnancy was confirmed; group 3 received folic acid but did not drink milk throughout the trial. Women who received folic acid tablets (groups 1 and 3) and/or milk (groups 1 and 2) continued their interventions throughout pregnancy. Control: did not take folic acid tablets and did not drink milk throughout the trial. Setting: facility‐based. rural and the urban maternal and child health centre for prenatal consultations Timing of intervention: periconceptional Moderators delivering: NA
**Outcomes**	Primary: birth body length and weight and Apgar score, serum folate concentrations
**Notes**	Funding: National Basic Research Program (973, 2010CB530403) from Ministry of Science and Technology of people's Republic of China. Conflict of interest: no conflict interest on the part of any of the authors

Risk of bias table

**Table:** 

Bias	Authors' judgement	Support for judgement
Random sequence generation (selection bias)	Unclear risk	Quote: “Three thousand five hundred and twenty‐six women had confirmed pregnancies and were randomized to receive liquid milk or not until delivery.” Comment: method of sequence generation was not described
Allocation concealment (selection bias)	Unclear risk	Quote: “further divided into four groups according to their assigned supplement regimen.” Comment: insufficient information to permit judgement
Similar baseline characteristics	Unclear risk	
Similar baseline outcome measurement	Unclear risk	
Blinding of participants and personnel (performance bias)	High risk	Quote: “Participants were instructed to drink a container of the liquid milk (243 ml) in the morning. As recommended by, participants were instructed to take a 400 μg FA tablet once a day.” “The organizers periodically recorded about drinking milk including stop or miss drinking or allergies.” Comment: organizers knew which patient was assigned to which intervention
Blinding of outcome assessment (detection bias)	Unclear risk	Comment: insufficient information to permit judgement
Incomplete outcome data (attrition bias)	Low risk	Quote: “3526 women had confirmed pregnancies and were randomized to receive liquid milk or not until delivery. Women gave up halfway for the following reasons; anaemia (*N* = 1), gastrointestinal disturbances (*N* = 5), dislike of the milk (*N* = 9), other reasons (*N* = 12).” Comment: reasons were given but the spread of drop out across groups were not given
Prevention of knowledge of allocated intervention	Unclear risk	
Protection against contamination	Unclear risk	
Selective reporting (reporting bias)	Unclear risk	Comment: insufficient information to permit judgement; and no protocol was found.
Other bias	Low risk	Comment: study seems to be free from other biases

Lou et al. (2004)

**Table:** 

**Methods**	Quasi‐experimental study determining the effects of a youth‐friendly intervention in Shanghai, China Study conducted between May 2000 to December 2001
**Participants**	*N* = 1220 unmarried young people from the intervention site and 1007 from the control site, including 1304 out‐of‐school youths and 923 high school students, were recruited, 15‐24 years of age. Non random sampling method was used to select the intervention and control sites
**Interventions**	Intervention: The intervention intended to build awareness and offer counselling and services related to sexuality and reproduction among unmarried youths, in addition to the routine program activities. Included three main areas of activities: (1) building awareness ‐ disseminating educational materials, playing instructional videos, giving lectures, and conducting small group activities. (2) provision of counselling ‐ youth health counselling centre was set up in the intervention town. (3) Enhancing access to services, specifically contraceptive services. Control: routine program activities, standard program and services Setting: Community based Timing of intervention: Moderators delivering: group activities were facilitated by research staff, full‐time young female counsellor was trained and made responsible for routine and telephone counselling, Trained interviewers assisted with surveys
**Outcomes**	Primary: Contraceptive use, condom use (ever contraceptive use, current regular contraceptive use, ever condom use, and contraceptive use at onset of sexual intercourse—if it occurred during the course of the intervention)
**Notes**	Funding: Department of Reproductive Health and Research, Special Program of Research, Development and Research Training in Human Reproduction, World Health Organization, Geneva, SwitzerlandDeclaration of interest: NA

Risk of bias table

**Table:** 

**Bias**	**Authors' judgement**	**Support for judgement**
Random sequence generation (selection bias)	Unclear risk	
Allocation concealment (selection bias)	Low risk	Quote: “To ensure comparability, the two towns were selected from the same district and similar in the level of social economics, geographic features, and social culture customs.” Comment: unit of allocation was by township
Similar baseline characteristics	High risk	Quote: “Differences were observed in terms of age distribution and education and occupation profiles. Gender disparities were also evident in both groups” Comment: baseline characteristics were not similar
Similar baseline outcome measurement	Low risk	Comment: baseline outcome measurements were similar
Blinding of participants and personnel (performance bias)	Unclear risk	
Blinding of outcome assessment (detection bias)	Unclear risk	
Incomplete outcome data (attrition bias)	Unclear risk	Quote: “A total of 91.7% were successfully followed up 20 months after the initial intervention, including 94.1% from the intervention site, and 88.8% from the control site.” Comment: not mentioned if missing outcome data were matched
Prevention of knowledge of allocated intervention	Unclear risk	Comment: insufficient information to permit judgement
Protection against contamination	Low risk	Comment: allocation was by town
Selective reporting (reporting bias)	Unclear risk	Comment: insufficient information to permit judgement; and no protocol was found.
Other bias	Low risk	Comment: study appears free from other bias

Martiniuk et al. (2003)

**Table:** 

**Methods**	A cluster randomised trial in Belize, Central America (cRCT) Study date not reported
**Participants**	Seven schools in Belize City were selected; 8 classrooms were randomised to the intervention arm and 11 classrooms to the control arm (*N* = 399). 13‐19 years old.
**Interventions**	Intervention: responsible sexuality education programme; which is a 3‐h scripted responsible sexuality education intervention which provides a framework for adolescents for decision making in relationships and provides unbiased information about sex and sexuality. Control: Allocation into intervention and control arms was achieved by flipping a coin. Setting: school level Timing of intervention: Moderators delivering:
**Outcomes**	Primary: knowledge associated with sex and sexuality Secondary: changes in attitudes and behavioural intent concerning sex and sexuality.
**Notes**	Association between gender and previous sexual experience at baseline. Other tables are not divided by gender Funding: NA Declaration of interest: NA

Risk of bias table

**Table:** 

**Bias**	**Authors' judgement**	**Support for judgement**
Random sequence generation (selection bias)	Low risk	Quote: “Allocation into intervention and control arms was achieved by flipping a coin” Comment: randomization method was adequate
Allocation concealment (selection bias)	Unclear risk	Comment: insufficient information to permit judgement
Similar baseline characteristics	Unclear risk	
Similar baseline outcome measurement	Unclear risk	
Blinding of participants and personnel (performance bias)	Low risk	Comment: blinding is not possible with the nature of intervention
Blinding of outcome assessment (detection bias)	Unclear risk	Comment: insufficient information to permit judgement
Incomplete outcome data (attrition bias)	Low risk	Quote: “Loss to follow‐up was similar in the intervention and the control groups (15% and 14% respectively).” Comment: this was adequately addressed
Prevention of knowledge of allocated intervention	Unclear risk	
Protection against contamination	Unclear risk	
Selective reporting (reporting bias)	Unclear risk	Comment: insufficient information to permit judgement; and no protocol was found.
Other bias	Unclear risk	Quote: “However, the two groups are not similar with respect to gender and previous sexual experience; this is likely to be due to the clustered nature of the data. Therefore, gender and previous sexual experience could act as confounders and were controlled for in the analysis.” Comment: possible confounders

Meekers (2000)

**Table:** 

**Methods**	Quasi‐experimental control group design in Soweto, South Africa
**Participants**	Young females aged 16‐20, analysis to females aged 17‐20. Remaining subsample includes *n* = 430 female adolescents.
**Interventions**	Intervention: Targeted social marketing program on reproductive health beliefs and behavior. This program promotes safer sex and distributes subsidized *Lovers Plus* condoms to traditional outlets, such as pharmacies and clinics, as well as nontraditional outlets, such as supermarkets, kiosks, and street vendors. The specific brand promotion and behavioral change activities include, a radio and television campaign promoting safer sex and condom use, billboard messages, press advertisements, point of sale materials (stickers and posters), and peer education among specific target groups. Control: not randomly assigned to control group Setting: Community based Timing of intervention: Moderators delivering:70 adolescents were trained in participatory media development, peer education and condom distribution.
**Outcomes**	Primary: Knowledge and awareness (contraceptives—including condoms, STI, HIV/AIDS)
**Notes**	pre‐ and post‐intervention surveys Funding: U.S Agency for International Development. AIDSCAPI Family Health International (FCO #52002, PSI Task Order No. 39), by the Office of Health and Nutrition, Global Bureau, and the Africa Bureau, USAID, under the terms of cooperative agreement No. HRN‐A‐00‐00021‐00. Additional support was provided by Population Services International, which has core funding from the British Department for International Development (DFID).Declaration of interest: NA

Risk of bias table

**Table:** 

**Bias**	**Authors' judgement**	**Support for judgement**
Random sequence generation (selection bias)	Unclear risk	
Allocation concealment (selection bias)	Low risk	Quote: “Soweto (intervention) and Umlazi (control)” Comment: unit of allocation was done by township
Similar baseline characteristics	High risk	Quote: “The two Soweto samples tend to be somewhat older than the Umlazi samples.” Comment: baseline characteristics were not similar
Similar baseline outcome measurement	High risk	Quote: “the most obvious difference between the two sites is the HIV prevalence rate.” Comment: baseline outcomes were not similar
Blinding of participants and personnel (performance bias)	Unclear risk	
Blinding of outcome assessment (detection bias)	Unclear risk	
Incomplete outcome data (attrition bias)	Unclear risk	Quote: “It is noted that this is not a panel design, i.e., the pre‐ and post‐intervention surveys are based on independent samples.” Comment: insufficient information to permit judgement
Prevention of knowledge of allocated intervention	Unclear risk	Comment: insufficient information to permit judgement
Protection against contamination	Low risk	Quote: “the two locations are over 500 kilometres apart minimizes the risk of contamination” Comment: protection was adequate
Selective reporting (reporting bias)	Unclear risk	Quote: “While data were collected on both males and females, data quality checks revealed that some of the interviewers used for the male follow‐up sample were not reliable. Consequently, the analyses in this paper are restricted to females” Comment: insufficient information to permit judgement and no protocol was found.
Other bias	Low risk	Comment: study was free from other bias

Mmbaga et al. (2017)

**Table:** 

**Methods**	Cluster‐randomised controlled trial in Dares Salaam, Tanzania Study conducted from 2011 to 2014
**Participants**	38 public primary schools were randomly selected, half to intervention and half to control. Total of 5091 male and female students (adolescents aged 12‐14) were recruited at baseline, and interviewed again at 6 (*n* = 4783) and 12 months (*n* = 4370).
**Interventions**	Intervention: PREPARE was designed to promote delayed sexual initiation and safer sexual behaviours among adolescents. It consists of 3 components implemented by teachers (6 lessons over 11 h), peer educators (3 lessons over 8 h), and health care providers (linking adolescents to information and services that may foster healthy sexuality). Control: half of the primary schools were assigned to the control group based on their size and geographic location.Setting: school‐level Timing of intervention: Moderators delivering: teachers, peer educators, health care providers
**Outcomes**	Primary: self‐reported sex initiation and condom use during the past 6 months.
**Notes**	Funding: EC Health research programme Declaration of interest: The authors declare that they have no competing interests.

Risk of bias table

**Table:** 

**Bias**	**Authors' judgement**	**Support for judgement**
Random sequence generation (selection bias)	Low risk	Quote: “For each of the 19 pairs of schools, one school was randomly allocated to the intervention group, and the second, the control group using computer generated randomisation.” Comment: random sequence generation was adequate
Allocation concealment (selection bias)	Low risk	Quote: “To ensure allocation sequence concealment, a statistician at the Medical Research Council, who did not have any knowledge of the schools, allocated the schools within each stratum to intervention and control arms of the study.” Comment: information taken from protocol. This was adequately done
Similar baseline characteristics	Unclear risk	
Similar baseline outcome measurement	Unclear risk	
Blinding of participants and personnel (performance bias)	Low risk	Comment: blinding was not possible due to the type of intervention and involvement of teachers, peer educators and health care providers delivering the intervention.
Blinding of outcome assessment (detection bias)	Unclear risk	Quote: “Members of the study team observed and rated approximately 25% of the teachers' intervention sessions” “All participants were given unique identification numbers at baseline and this number was written in the baseline questionnaire. The participant was given an envelope with his/her unique identification number which was produced during follow up before completing the 6 and 12 months follow up questionnaires.” Comment: it is unclear whether those that collected the data and checked the identification numbers were the same researchers that performed the analysis.
Incomplete outcome data (attrition bias)	Low risk	Quote: “This loss to follow up at 12 months did not differ significantly by intervention status. The main reasons for the loss to follow up were students' transfer to schools outside the study area and absenteeism” Comment: missing outcome date is balanced across groups
Prevention of knowledge of allocated intervention	Unclear risk	
Protection against contamination	Unclear risk	
Selective reporting (reporting bias)	Low risk	Comment: outcome variables were reported in the protocol
Other bias	Unclear risk	Quote: “all behavioural estimates were based on participant's self‐reports, which could be liable to desirability bias” Comment: possible bias

Muro et al. (1999)

**Table:** 

**Methods**	Randomised controlled trial in Tanzania Study conducted from August to November 1996
**Participants**	*N* = 237 adolescent girls aged between 14 to 17 years, from five schools. Both anaemic and nonanaemic girls were included.
**Interventions**	Intervention: two groups; (1) Iron‐folic acid only (iron sulphate 65 mg and folic acid 0.25 mg): *n* = 39 (school 3). (2) Iron‐folic acid (same supplement) and participated in weekly communication sessions.: *n* = 78 (school 1 & 2) Control: no intervention: *n* = 120 (school 5) Setting: community Timing of intervention: preconception Moderators delivering:trained class teachers
**Outcomes**	Primary: haemoglobin, anaemia, adherence, adverse effects, knowledge, low birthweight
**Notes**	Funding: NA Declaration of interest: NA

Risk of bias table

**Table:** 

**Bias**	**Authors' judgement**	**Support for judgement**
Random sequence generation (selection bias)	High risk	Quote: “Each group should have contained a similar number of girls. However, this goal was not reached, because after the schools had been randomly assigned to the three groups, the parents of the girls in school 4, who were assigned to receive iron supplementation without communication,did not approve of their daughters receiving iron tablets. The girls from this school were added to the nonintervention group” Comment: not adequately done
Allocation concealment (selection bias)	High risk	See above
Similar baseline characteristics	Unclear risk	
Similar baseline outcome measurement	Unclear risk	
Blinding of participants and personnel (performance bias)	High risk	Comment: blinding was not performed
Blinding of outcome assessment (detection bias)	Unclear risk	Comment: there is insufficient information to permit a judgement
Incomplete outcome data (attrition bias)	Low risk	Comment: 100% of the participants finished the treatment.
Prevention of knowledge of allocated intervention	Unclear risk	
Protection against contamination	Unclear risk	
Selective reporting (reporting bias)	Unclear risk	Comment: insufficient information to permit judgement, and no protocol was found.
Other bias	Low risk	Comment: the study appears to be free of other bias.

Okonofua et al. (2003)

**Table:** 

**Methods**	A randomized controlled trial in Nigeria (cluster RCT)Study conducted from September 1997 to July 1998
**Participants**	12 schools, 1896 and 1858 Nigerian youths 14‐20 years of age were enrolled in the pre‐ and post‐intervention surveys. Sampled single‐sex schools and co‐educational schools separately.
**Interventions**	Intervention: implemented in 4 schools, consisted of community participation, peer education, public lectures, health clubs in the schools, and training of STD treatment providers, including those with no formal training. A reproductive health club was established in each school, some of the members of the club were trained as peer educators and a final component of the intervention involved training of health providers.Control: eight randomly selected control schools and received no intervention. Two control groups: 4 from same region as intervention (Benin City), and 4 from a different region to minimize contamination (Ekpoma).Setting: school‐levelTiming of intervention:Moderators delivering: peer educators, health providers (medical practitioners, patent medicine dealers and pharmacists)
**Outcomes**	Primary: knowledge of STD symptoms, condom use, treatment seeking behavior among youths experiencing STD symptoms, the proportion of youths who had experienced symptoms of an STD in the 6 months prior to interview, notification of partner(s) by adolescents who hadSTDs.
**Notes**	Only some results are segregated by genderICC value of 0.06 used from Mathews et al., 2016 as they have the same study setting (schools).Funding: the John D. and Catherine T. MacArthur Foundation and the Rockefeller FoundationDeclaration of interest: NA

Risk of bias table

**Table:** 

**Bias**	**Authors' judgement**	**Support for judgement**
Random sequence generation (selection bias)	Unclear risk	Quote: “randomisation was applied at the school level” Comment: the method of randomisation of the schools was not discussed.
Allocation concealment (selection bias)	Unclear risk	Comment: insufficient information to permit judgement
Similar baseline characteristics	Unclear risk	
Similar baseline outcome measurement	Unclear risk	
Blinding of participants and personnel (performance bias)	Low risk	Comment: not possible to blind participants
Blinding of outcome assessment (detection bias)	Unclear risk	Comment: insufficient information to permit judgement
Incomplete outcome data (attrition bias)	Low risk	Quote: “A further source of potential bias was that resulting from possible loss to follow‐up…The study was designed to minimize loss to follow‐up by conducting the pre‐ and post‐intervention surveys among adolescents in senior classes 4 and 5, to ensure their availability 12 months after baseline.” Comment: this was adequately addressed
Prevention of knowledge of allocated intervention	Unclear risk	
Protection against contamination	Unclear risk	
Selective reporting (reporting bias)	Unclear risk	Comment: insufficient information to permit judgement; and no protocol was found.
Other bias	Low risk	Comment: the study appears to be free from other sources of bias

Pandey et al. (2016)

**Table:** 

**Methods**	Quasi‐randomized and natural experiment in India Study conducted during 2014
**Participants**	Adolescents in rural areas of selected districts of Bihar. A survey was conducted of 371 and 679 young men and women from control areas, and 789 and 1382, respectively, from intervention areas. 40 selected intervention villages and 20 selected control villages. Participants were aged 13–21 at time of training.
**Interventions**	Intervention: Prachar Project's reproductive health training programme for adolescents, which consists of three day training and focuses on addressing adolescents' need for information, contraceptive supplies, parental and community support, and a youth‐friendly health system. This aimed specifically at raising awareness and understanding of sexual and reproductive importance of delayed childbearing and spacing of pregnancies, and sources of services among unmarried adolescents. Two types of project settings: one in which no other PRACHAR Phase III activities were conducted (standalone settings) and a second (comprehensive settings) in which the adolescent training programme was conducted along with other activities of PRACHAR Phase III among married women and men more generally. Control: a cohort of similar young people not exposed to the programme. Setting: community‐level Timing of intervention: Moderators delivering: Two female trainers implemented the programme for girls, and one male and one female did so for boys.
**Outcomes**	Primary: young people's awareness of sexual and reproductive health matters, their gender role attitudes and such behaviours as delayed marriage and postponement of the first birth; the extent of safe and wanted pre‐marital sexual experiences; agency (particularly among young women), notably with regard to participation in marriage related decision‐making and other life choices (education, work, control over resources); and timely access to sexual and reproductive health services Namely knowledge and behaviour Secondary
**Notes**	Main outcomes interested in: pre‐marital sexual experiences (if any, including age at sexual initiation, partners, and contraception and condom use), and awareness about sexual and reproductive health matters. Funding: David and Lucile Packard Foundation Declaration of interest: NA

Risk of bias table

**Table:** 

**Bias**	**Authors' judgement**	**Support for judgement**
Random sequence generation (selection bias)	Unclear risk	
Allocation concealment (selection bias)	Low risk	Comment: unit of allocation as by villages
Similar baseline characteristics	High risk	Quote: “Findings indicate that while largely similar, despite village‐ as well as sample‐level matching on educational attainment, young people in intervention areas were marginally better off in terms of household economic status, educational attainments, and economic activity than those in control areas.” Comment: baseline characteristics were not adequately similar
Similar baseline outcome measurement	Unclear risk	Quote: “The training programme was not evaluated and hence no baseline data were available for assessing longer‐term changes among trainees” Comment: insufficient information to permit judgement
Blinding of participants and personnel (performance bias)	Unclear risk	
Blinding of outcome assessment (detection bias)	Unclear risk	
Incomplete outcome data (attrition bias)	Unclear risk	Comment: insufficient information to permit judgement
Prevention of knowledge of allocated intervention	Unclear risk	Comment: insufficient information to permit judgement
Protection against contamination	Low risk	Comment: allocation was by village
Selective reporting (reporting bias)	Unclear risk	Comment: insufficient information to permit judgement, and no protocol was found.
Other bias	Low risk	Comment: study appears free of other bias

Rosenthal (2008)

**Table:** 

**Methods**	Randomised, double‐blind control supplementation trial conducted in Choloma, Honduras. Study conducted between April and June 2005
**Participants**	140 female factory workers aged 18 to 49 years in Choloma, Honduras were randomly assigned to 1 of 2 groups
**Interventions**	Intervention: group 1 received a daily dosage of 1000 μg (1 mg) folic acid; group 2 received a once weekly dosage of 5000 μg (5 mg). Serum folate and red blood cell folate levels were determined by radioimmunoassay at baseline, 6 weeks and 12 weeks. Both groups received folic acid at different doses and regimens. Control: No control Setting: Community? information fair for all female workers, at work pills were administered Timing of intervention: Moderators delivering:folic acid pills were administered to the study subjects by two factory nurses.
**Outcomes**	Primary: serum and red blood cell folate
**Notes**	Funding: Project Healthy Children, Weston, Massachusetts. NOW Foods donated the folic acid tablets for the studyDeclaration of interest: acknowledged

Risk of bias table

**Table:** 

**Bias**	**Authors' judgement**	**Support for judgement**
Random sequence generation (selection bias)	Low risk	Quote: “A randomisation program was used to allocate volunteers into the two groups” Comment: adequately done
Allocation concealment (selection bias)	Low risk	Quote: “The 1 mg, 5 mg and placebo pills were of the same colour and size” Comment: adequately done
Similar baseline characteristics	Unclear risk	
Similar baseline outcome measurement	Unclear risk	
Blinding of participants and personnel (performance bias)	Low risk	Quote:“neither the authors nor the health staff or volunteers were aware of the subject's dose category until the study was completed. ”Comment: adequately done
Blinding of outcome assessment (detection bias)	Low risk	Quote: “neither the authors nor the health staff or volunteers were aware of the subject's dose category until the study was completed.” Comment: adequately done
Incomplete outcome data (attrition bias)	Low risk	Quote: Follow‐up rates at the end of the study were fifty‐eight out of seventy (82.9%) for the 5 mg once weekly dosage group and fifty out of seventy(71.4%) for the 1 mg daily dosage group. Comment: difference is <30% Quote: “Eleven volunteers in the 5 mg/week group dropped out from the study because of the following reasons: four resigned from work, three became pregnant, one had low B12 and three did not want to continue in the trial. Among the women in the 1 mg/d group, eight dropped out because they resigned their positions, two were fired, five became pregnant, one had low B12, two had burning and discomfort, and two did not give any specific reason.” “Furthermore, in the majority of cases the cause for dropping out was not linked to the trial per se.” Comment: reason for not continuing were provided
Prevention of knowledge of allocated intervention	Unclear risk	
Protection against contamination	Unclear risk	
Selective reporting (reporting bias)	Unclear risk	Comment: insufficient information to permit judgement, and no protocol was found.
Other bias	High risk	Quote: “(i) the two groups had different time lags between last pill taken and blood draw, which could have biased the group receiving the daily dosage to observed higher levels in serum and red blood cell folate; (ii) we were not able to observe the long‐term effect of the two dosage regimens as the intervention and measurement of folic acid levels spanned only 12 weeks; and (iii) we did not assess the un metabolized folic acid in serum nor the dietary intake of the participants. Recent research has demonstrated that, with higher dosages of folic acid, one can expect amounts of un metabolized folic acid to be found in blood. Our study was not designed to evaluate un metabolized folic acid.” Comment: other biases were present

Ross et al. (2007)

**Table:** 

**Methods**	Quasi experimental study. A community‐randomised trial in Tanzania (cluster RCT) Study conducted between late 1998 and April 2002
**Participants**	Twenty communities were randomly allocated, 9645 adolescents recruited in late 1998 before entering years 5, 6 or 7 of primary school. Minimum age was 14 and mean age was 15.7 years.
**Interventions**	Intervention: This intervention aimed to provide young people with the knowledge and skills to enable them to delay sexual debut, reduce sexual risk‐taking by sexually active youth and increase their appropriate use of sexual health services. Community activities; teacher‐led, peer‐assisted sexual health education in Years 5‐7 of primary school; training and supervision of health workers to provide “youth‐friendly” sexual health services; and peer condom social marketing. Control: standard activities Setting: Community based Timing of intervention: Moderators delivering: teachers, health workers, peer‐elected youth for condom distribution, local youth groups
**Outcomes**	Primary: Knowledge, attitude, HIV, herpes simplex virus 2 (HSV)
**Notes**	Funding: supported by grants from the European Commission, Development Cooperation Ireland (now Irish Aid), UK Medical Research Council, UNAIDS, and UK Department for International Development.Declaration of interest: NA

Risk of bias table

**Table:** 

**Bias**	**Authors' judgement**	**Support for judgement**
Random sequence generation (selection bias)	Low risk	Quote: “The 20 study communities were grouped into three strata, according to expected risk of HIV infection.” (Hayes et al. 2005) Comment: this was adequately done
Allocation concealment (selection bias)	Unclear risk	Comment: insufficient information to permit judgement
Similar baseline characteristics	Unclear risk	
Similar baseline outcome measurement	Unclear risk	
Blinding of participants and personnel (performance bias)	Low risk	Comment: participants could not be blinded due to the nature of the intervention
Blinding of outcome assessment (detection bias)	Low risk	Quote: “Sera were tested for HIV‐1 and HIV‐2 using the Murex HIV Ag/Ab Combination ELISA (Murex Biotech).” ”Sera were tested for antibodies to HSV2 using a monoclonal enzyme immunoassay” Comment: Outcome measurement not affected by lack of blinding
Incomplete outcome data (attrition bias)	Low risk	Quote: “Follow‐up rates were similar in intervention (72%) and comparison (74%) communities” Comment: high amount of loss to follow up—however the reasons were stated
Prevention of knowledge of allocated intervention	Unclear risk	
Protection against contamination	Unclear risk	
Selective reporting (reporting bias)	Low risk	Quote: “The predefined primary trial outcomes were HIV seroincidence during follow‐up and HSV2 seroprevalence at final survey. Secondary outcomes were six further biological, five behavioural, one attitudinal and three knowledge outcomes” Comment: pre specified outcomes were reported
Other bias	Low risk	Comment: no evidence to suggest this

Shah and Gupta (2002)

**Table:** 

**Methods**	Randomised‐controlled trial based in Nepal Study conducted from March 1998 to March 1999
**Participants**	Health adolescent girls aged 11‐18 year old (*n* = 209) randomised to 3 groups. *N* = 181 completed the trial.
**Interventions**	Intervention: weekly versus daily iron and folic acid supplementation. (1) IFA (350‐mg ferrous sulfate and 1.5‐mg folic acid combination) once daily for 90 to 100 days. (*n* = 70). (2) IFA (350‐mg ferrous sulfate and 1.5‐mg folic acid combination) once weekly for 14 weeks. (*n* = 72) Control: *n* = 72 Setting: School‐level Timing of intervention: preconception Moderators delivering: parents
**Outcomes**	Primary: pre‐ and post‐ prevalence of anaemia and change in hematocrit
**Notes**	Funding: Research Committee of B.P. Koirala Institute of Health Sciences, DharanDeclaration of interest: NA

Risk of bias table

**Table:** 

**Bias**	**Authors' judgement**	**Support for judgement**
Random sequence generation (selection bias)	Unclear risk	Quote: “Subject were randomly assigned to 1 of 3 groups” Comment: method of sequence generation was not described
Allocation concealment (selection bias)	Unclear risk	Comment: there is insufficient information to permit judgement
Similar baseline characteristics	Unclear risk	
Similar baseline outcome measurement	Unclear risk	
Blinding of participants and personnel (performance bias)	High risk	Comment: consumption of the supplements was performed under supervision. While the participants were not blinded it is unlikely this had an effect on the results
Blinding of outcome assessment (detection bias)	Unclear risk	Comment: insufficient information to permit judgement
Incomplete outcome data (attrition bias)	Low risk	Comment: of the 209 girls who met the inclusion criteria, 181 completed the study (87%); losses were balanced among groups. Severe adverse effects, noncompliance to treatment, and non availability for final hematocrit measurement were listed as reasons for losses. Numbers of subjects who left for each reason was not documented
Prevention of knowledge of allocated intervention	Unclear risk	
Protection against contamination	Unclear risk	
Selective reporting (reporting bias)	Unclear risk	Comment: insufficient information to permit judgement and no protocol was found
Other bias	High risk	Comment: subjects in the daily regimen group were not explicitly supervised while those in the weekly group were supervised

Shobha and Sharada (2003)

**Table:** 

**Methods**	Randomised‐controlled trial in India Study date not reported
**Participants**	*N* = 244 girls belonging to the 13‐15 years age group, from a low socioeconomic background.Subjects belonging to each grade of anaemia were further randomly divided into two subgroups (*n* = 203 were anaemic)
**Interventions**	Intervention: daily versus twice weekly iron for a duration of 12 weeks. (1) daily (60 mg iron, 0.5 mg folic acid) (*n* = 102) (2) Wed & Sat (60 mg iron, 0.5 mg folic acid) (*n* = 101) All girls received anthelmintic treatment one week before supplementation Control: NA Setting: school‐level Timing of intervention: preconception Moderators delivering: investigator
**Outcomes**	Primary: haemoglobin, adverse events
**Notes**	Funding: None Declaration of interest: none stated

Risk of bias table

**Table:** 

Bias	Authors' judgement	Support for judgement
Random sequence generation (selection bias)	Unclear risk	Quote: “Subjects belonging to each grade of anemia were further randomly divided into two subgroups” Comment: method of sequence generation was not described
Allocation concealment (selection bias)	Unclear risk	Comment: insufficient information to permit judgement
Similar baseline characteristics	Unclear risk	
Similar baseline outcome measurement	Unclear risk	
Blinding of participants and personnel (performance bias)	High risk	Quote: “Supplementation was carried out under the strict supervision of the investigator” Comment: personal were not blinded, there was no mention of blinding the participants
Blinding of outcome assessment (detection bias)	Unclear risk	Comment: insufficient information to permit judgement
Incomplete outcome data (attrition bias)	Unclear risk	Comment: insufficient information to permit judgement
Prevention of knowledge of allocated intervention	Unclear risk	
Protection against contamination	Unclear risk	
Selective reporting (reporting bias)	Unclear risk	Comment: insufficient information to permit judgement and no protocol was found.
Other bias	Low risk	Comment: the study appears to be free of other bias

Shuey et al. (1999)

**Table:** 

**Methods**	Quasi‐randomised study based in Uganda Study conducted from February 1994 to November 1996
**Participants**	A cross‐sectional sample of students 13–14 years, in their final year of primary school, was surveyed before and after 2 years of interventions. *N* = 400 at follow up time (280 in intervention group and 120 in control group).
**Interventions**	Intervention: School health education programme in primary schools. This programme aimed to improve access to information and other resources for healthy sexual behavior decision making, improve adolescent to adolescent interaction regarding information and decision making (relating to AIDS, sexuality and health), and improving the quality of the existing district educational system in the implementation of the school health curriculum and in counselling/advice giving to students. This intervention consisted of 9 activities involving the community, parents, local leaders, teachers, students and school health clubs. Control: A county adjacent to one of the implementation counties was surveyed as part of the baseline survey and this county served as a control. Students in the control area were exposed to the standard school health and AIDS education programme of Uganda Setting: school‐level Timing of intervention: Moderators delivering: one full time health educator, staff already present on the district education and health teams, Senior men and women tutors are teachers
**Outcomes**	Primary: been sexually active (abstaining) Secondary
**Notes**	Only data segregated by gender: Tables IV. *Proportions of P7 students who reported they had “played sex” or participated in sexual intercourse separated by gender* *Funding:* the ODA/UK, the Mercury Phoenix Trust, the Presiding Bishop's Fund of New York, AMREF Italy and AGIP. Declaration of interest: NA

Risk of bias table

**Table:** 

**Bias**	**Authors' judgement**	**Support for judgement**
Random sequence generation (selection bias)	Unclear risk	
Allocation concealment (selection bias)	Unclear risk	Comment: insufficient information to permit judgement
Similar baseline characteristics	Unclear risk	Comment: insufficient information to permit judgement
Similar baseline outcome measurement	High risk	Quote: “In 1994, 42.9% (123 of 287) of students in the intervention group described themselves as sexually active, while 25.7% (29 of 113) in the control group did so.” Comment: baseline characteristics were not adequately similar
Blinding of participants and personnel (performance bias)	Unclear risk	
Blinding of outcome assessment (detection bias)	Unclear risk	
Incomplete outcome data (attrition bias)	Unclear risk	Quote: “The same procedure was repeated in the November 1996 survey, using the P7 class that was present 2 years later.” Comment: participants in control group were 113 and 120 at baseline and follow up respectively. Participants in intervention group were 287 and 280 at baseline and follow up respectively. Although the number of respondents is similar, the participants themselves could be different.
Prevention of knowledge of allocated intervention	Unclear risk	Comment: insufficient information to permit judgement
Protection against contamination	Low risk	Comment: adjacent county was used as control site
Selective reporting (reporting bias)	Unclear risk	Comment: insufficient information to permit judgement and no protocol was found
Other bias	Unclear risk	Quote: “Students were asked to state their age at the time of first intercourse. In 1994 the question must have been misunderstood, as 17 of 120 (14.1%) in the intervention group and five of 27 (18.5%) in the control group replied 5 years of age or less including seven who answered 0 years.In 1996, only one of 31 in the intervention group and none of 32 in the control group gave such an answer. Mean age of first intercourse was estimated after those answers under the age of 6 years were excluded.” Comment: possible measurement bias

Soekarjo et al. (2004)

**Table:** 

**Methods**	Randomised‐controlled trial in Indonesia Study conducted from November 1996 to May 1997
**Participants**	*N* = 1757 girls and 1859 boys, aged 12–15 years, in 24 Junior High Schools.
**Interventions**	Intervention: 3 groups: (1)weekly 10 000 IU vitamin A (*n* = 970), (2) weekly 60 mg elemental iron (as ferrous sulphate) plus 250 mg folate (*n* = 978) or (3) weekly 10 000 IU vitamin A and 60 mg elemental iron plus 250 mg folate (*n* = 1042) For the iron‐folic acid group there is complete data for 488 girls. Duration: 14 weeks Control: no supplementation (*n* = 309) Setting: school‐level Timing of intervention: preconception Moderators delivering: field workers
**Outcomes**	Primary: Haemoglobin, Anaemia, low serum retinol
**Notes**	Funding: USAID Declaration of interest: NA

Risk of bias table

**Table:** 

**Bias**	**Authors' judgement**	**Support for judgement**
Random sequence generation (selection bias)	Unclear risk	Quote: “Adolescents from 15 schools (four U‐MTs, seven U‐SMP and four R‐SMP) (n¼2990) were randomly selected to receive weekly supplements, while adolescents in the other nine schools (three U‐MTs, two U‐SMP and four R‐SMP) served as controls (n¼1750).”Comment: method of sequence generation was notmentioned
Allocation concealment (selection bias)	Low risk	Comment: since this is a cluster trial, it is unlikely a selection bias at individual level
Similar baseline characteristics	Unclear risk	
Similar baseline outcome measurement	Unclear risk	
Blinding of participants and personnel (performance bias)	High risk	Quote: “All pupils were aware of which supplement(s) they were taking”Comment: Participants: were aware of the treatment;Personnel: were aware of the treatment;
Blinding of outcome assessment (detection bias)	Low risk	Comment: while outcome assessors were aware of the treatment allocation the majority of outcomes were subjective
Incomplete outcome data (attrition bias)	Low risk	Quote: “4810 out of 5116 participants study (96%). Subjects who dropped out were slightly older than those who remained in the study, and more subjects in the urban religious schools dropped out compared to the other school types. Otherwise, there were no differences between those who finished the study and those who did not.” “Dropout during the study (5.1%) was mainly caused by absenteeism on several consecutive days during the end‐line data collection. There was no difference in dropout rate between the four groups.” Comment: Missing outcome data were matched across groups
Prevention of knowledge of allocated intervention	Unclear risk	
Protection against contamination	Unclear risk	
Selective reporting (reporting bias)	Unclear risk	Comment: outcomes mentioned in the methods were explored in the results section but no protocol was found
Other bias	Low risk	Comment: the study appears to be free of other bias.

Speizer et al. (2001)

**Table:** 

**Methods**	Quasi‐experimental design in Cameroon (Entre Nous Jeunes) Study conducted from November 1997 to April 1999
**Participants**	A random household sample of adolescents aged 10‐25. At baseline, 402 from intervention and 400 in comparison and at follow‐up there were 405 in intervention and 413 in comparison.
**Interventions**	Intervention: peer education programme that educated peer educators in information techniques for group discussions and on reproductive health related topics. The peer educators worked within their communities through discussion groups, one‐on‐one meetings, and health and sport association gatherings to inform and refer their peers to health/social centres for services. Control: comparison community, Mbalmayo Setting: community‐level Timing of intervention: Moderators delivering: peer‐educator
**Outcomes**	Primary: “spontaneous” knowledge (modern contraceptives, symptoms of STIs), protective behaviours (condom use, modern contraceptives)
**Notes**	Funding: Family Health and AIDS in West and Central Africa Project to Tulane University from USAID Declaration of interest: NA

Risk of bias table

**Table:** 

**Bias**	**Authors' judgement**	**Support for judgement**
Random sequence generation (selection bias)	Unclear risk	
Allocation concealment (selection bias)	Low risk	Quote: “…conducted in the intervention community, Nkongsamba, and in the comparison community, Mbalmayo” Comment: unit of allocation was by community/city
Similar baseline characteristics	High risk	Quote: “Girls in the sample from Mbalmayo are more likely to be able to read and more likely never to have been married than are the girls in the sample from Nkongsamba.” Comment: baseline characteristics were not similar
Similar baseline outcome measurement	High risk	Comment: baseline outcome measurements were not similar
Blinding of participants and personnel (performance bias)	Unclear risk	
Blinding of outcome assessment (detection bias)	Unclear risk	
Incomplete outcome data (attrition bias)	Unclear risk	Comment: insufficient information to permit judgement
Prevention of knowledge of allocated intervention	Unclear risk	Comment: insufficient information to permit judgement
Protection against contamination	Low risk	Comment: allocation was done by community
Selective reporting (reporting bias)	Unclear risk	Comment: insufficient information to permit judgement and no protocol was found
Other bias	Low risk	Comment: study appears free from other bias

Vergel et al. (1990)

**Table:** 

**Methods**	Community‐based intervention, Cuba Study date not reported
**Participants**	*N* = 81, Nonpregnant women with a history of a previous NTD birth
**Interventions**	Intervention: 5 mg folic acid/day for not less than one menstrual period before conception until the 10th week of pregnancy. (i) fully supplemented (FS) who had followed a full regime of folic acid supplementation; (ii) partially supplemented (PS)patients, whose duration of supplementation fell short of the full regime Control: (iii) un supplemented (US) patients, who were in the early stage of pregnancySetting: Facility‐level Provincial Genetic Department Timing of intervention: preconception, periconceptional, prenatal Moderators delivering: NA
**Outcomes**	Primary: outcome of pregnancy (Recurrence of NTD, miscarriage) Secondary: previous reproductive history (total number of previous pregnancies, NTDs (%), spontaneous abortion (%))
**Notes**	Funding: NA Declaration of interest: NA

Risk of bias table

**Table:** 

**Bias**	**Authors' judgement**	**Support for judgement**
Random sequence generation (selection bias)	Unclear risk	
Allocation concealment (selection bias)	High risk	Quote: “The patients were classified into three groups: (i) fully supplemented (FS) who had followed a full regime of folic acid supplementation; (ii) partially supplemented (PS) patients, whose duration of supplementation fell short of the full regime; and (iii) un supplemented (US) patients, who were in the early stage of pregnancy (<15 weeks)” Comment: there was no centralized randomisation scheme—groups were analysed depending on their compliance to taking the supplement
Similar baseline characteristics	High risk	Quote: “There was a difference between the number of study pregnancies immediately preceded by a NTD in FS, 92.5 per cent; in PS, 100 per cent; and in US patients 80.6 per cent” Comment: not similar
Similar baseline outcome measurement	Unclear risk	Comment: insufficient information to permit judgement
Blinding of participants and personnel (performance bias)	Unclear risk	
Blinding of outcome assessment (detection bias)	Unclear risk	
Incomplete outcome data (attrition bias)	Unclear risk	Comment: not stated explicitly that no patient lost to follow up
Prevention of knowledge of allocated intervention	Low risk	Comment: the outcomes were objective
Protection against contamination	Unclear risk	Comment: insufficient information to permit judgement
Selective reporting (reporting bias)	Unclear risk	Comment: insufficient information to permit judgement and no protocol was found
Other bias	Low risk	Comment: study appears free from other bias

Walker et al. (2006)

**Table:** 

**Methods**	Cluster randomised controlled trial in Mexico (cluster RCT) Study conducted from 2001 to June 2003
**Participants**	10,954 10th grade to 12th grade high school students in Morelos, 15 years to 18 years, 48% male, 52% female. There were 7308 students at the one year follow up.
**Interventions**	Intervention: (1) HIV education, skills‐ building, cultural values, contraceptive promotion (condoms), (2) HIV education, skills‐ building, cultural values plus contraceptive education (EC plus condoms and their access) Control: biology‐ based sex education Setting: school‐based Timing of intervention: Moderators delivering: teachers
**Outcomes**	Primary: Initiation of intercourse, use of condom at last sex, use of hormonal contraceptive Secondary: knowledge and attitudes about HIV and emergency contraception; and attitudes and confidence about condom use.
**Notes**	Duration of follow up: 16 months Loss to follow up: 33.3% Two of the intervention schools were included in the control group because they did not teach the intervention course Funding: World Aids Foundation and Mexican National Institute of Public Health Declaration of interest: None declared

Risk of bias table

**Table:** 

Bias	Authors' judgement	Support for judgement
Random sequence generation (selection bias)	Low risk	Quote: “We selected schools and asked them to participate, on the basis of stratified random sampling (stratified by degree of urbanisation), with sampling proportional to school size” Comment: this was adequately done
Allocation concealment (selection bias)	Unclear risk	Comment: insufficient information to permit judgement
Similar baseline characteristics	Unclear risk	
Similar baseline outcome measurement	Unclear risk	
Blinding of participants and personnel (performance bias)	Low risk	Comment: participants (students) and personnel (teachers) could not be blinded to the intervention due to active participation
Blinding of outcome assessment (detection bias)	Unclear risk	Comment: insufficient information to permit judgement
Incomplete outcome data (attrition bias)	Unclear risk	Comment: per‐protocol analysis was done. But analysis took the cluster sample design into account
Prevention of knowledge of allocated intervention	Unclear risk	
Protection against contamination	Unclear risk	
Selective reporting (reporting bias)	Unclear risk	Comment: insufficient information to permit judgement and no protocol was found
Other bias	Low risk	Comment: the study appears to be free from other sources of bias

Wehby et al. (2012)

**Table:** 

**Methods**	Randomised, double‐blinded study in Brazil Study conducted from 2004 to 2007
**Participants**	2508 nonpregnant women of reproductive age from craniofacial clinics in Brazil who had nonsyndromic or isolated oral clefts or had at least 1 natural child with nonsyndromic or isolated oral clefts
**Interventions**	Intervention: assigned to receive either a single pill of 4000 μg (4 mg) folic acid or 400 μg (0.4 mg) of folic acid daily to be continued until the end of the first trimester. All study participants took folic acid. Control: historical control group. Setting: facility based, clinic Timing of intervention:taken on a daily basis during preconception and up to 3 months of pregnancy periconception, prenatal Moderators delivering:
**Outcomes**	Primary: difference in NSCL/P recurrence rates between the two groups and the associated confidence interval. Secondary: serum and/or red cell folate.
**Notes**	Funding: NIH/NICHD grant U01HD040561 awarded as part of the Global Network for Women's and Children's Health Research and by NIH/NIDCR grant U01 DE017958. Declaration of interest: no competing interests

Risk of bias table

**Table:** 

Bias	Authors' judgement	Support for judgement
Random sequence generation (selection bias)	Unclear risk	
Allocation concealment (selection bias)	Low risk	Quote: “The randomization sequence will link the treatment assignment (0.4 mg or 4 mg) to a sequential list of serial numbers to be used for the study pill boxes. The Data Center will generate the randomization sequence.” “All pills, regardless of their folic acid concentration, will be identical in size, shape, and color and will be provided in identical packaging.” Comment: adequately done
Similar baseline characteristics	Low risk	Comment: baseline outcomes were similar
Similar baseline outcome measurement	Low risk	Comment: baseline characteristics were similar
Blinding of participants and personnel (performance bias)	Unclear risk	
Blinding of outcome assessment (detection bias)	Unclear risk	
Incomplete outcome data (attrition bias)	High risk	Quote: “Of the randomised women, 913 chose to discontinue their participation in the study before pregnancy, and five pregnant women were lost to follow up.” Comment: in the 0.4 mg FA group a total of 1,106 withdrew or were lost to follow up, a similar amount of women dropped out of the study for the 4.0 mg FA group 1,093 (Figure 1). Therefore a substantial attrition rate was observed.
Prevention of knowledge of allocated intervention	Low risk	Quote: “The Data Center will maintain the randomisation sequence at RTI headquarters in NC. Revealing the individual assignment will be highly restricted and will only be done if deemed clinically necessary.” Comment: Probably done
Protection against contamination	Unclear risk	Comment: insufficient information to permit judgement
Selective reporting (reporting bias)	Low risk	Comment: all outcomes in the protocol were mentioned but not extensively discussed
Other bias	Unclear risk	Quote: “One limitation of the study is introducing some changes in recruitment strategies (such as limiting length of participation to 3 years) and inclusion/exclusion criteria (such as not including cleft palate only and excluding women using injectable contraceptives) while the study was ongoing” Comment: other biases were present

Ybarra et al. (2013)

**Table:** 

**Methods**	Parallel‐group randomised controlled trial in Uganda (RCT) Study conducted from February 2011 to September 2011
**Participants**	Participants were 12 years of age and older and enrolled in one of four secondary schools. Participants were randomised to the intervention (*n* = 183) or control (*n* = 183) arm; 91 intervention participants were further randomised to the booster. The mean age of participants was 16.1 years (SD = 1.4).
**Interventions**	Intervention: CyberSenga, was a 5‐h online healthy sexuality program. Half of the intervention group was further randomised to receive a booster at 4 months post‐intervention. This programme included: (1) Information about HIV; (2) Decision Making and Communication; (3) Motivations to be healthy; (4) How to use a condom to be healthy; (5) Healthy relationships and (6) Review. Control: “treatment as usual” (i.e., school‐delivered sexuality programming). Setting: school‐level Timing of intervention: Moderators delivering: media, research assistants
**Outcomes**	Primary: (1) condom use and (2) abstinence in the past three months at 6‐months' post‐intervention. Secondary: abstinence at 3 month's post‐intervention; and 6‐month outcomes by booster exposure.
**Notes**	Small sample of females: 16.1% (*n* = 59) Funding: National Institute of Mental Health Declaration of interest: no competing interests

Risk of bias table

**Table:** 

Bias	Authors' judgement	Support for judgement
Random sequence generation (selection bias)	Low risk	Quote: “Youth were then randomly selected by the research team using randomizer.org.” Comment: random sequence generation was adequately done
Allocation concealment (selection bias)	Low risk	Quote: “Participants were randomised at the end of the baseline survey. As such, all participants were blind to their arm assignment at enrolment.” Comment: allocation concealment was adequate
Similar baseline characteristics	Unclear risk	
Similar baseline outcome measurement	Unclear risk	
Blinding of participants and personnel (performance bias)	Low risk	Comment: blinding was not possible due to the nature of the intervention
Blinding of outcome assessment (detection bias)	Unclear risk	Comment: insufficient information to permit judgement
Incomplete outcome data (attrition bias)	Low risk	Quote: “Ninety‐two percent of intervention and 93% of control participants provided six‐month follow‐up data” Comment: missing outcome data is balance across the groups
Prevention of knowledge of allocated intervention	Unclear risk	
Protection against contamination	Unclear risk	
Selective reporting (reporting bias)	Low risk	Quote: “The study design initially proposed to examine the effects of exposure to CyberSenga on unprotected sex over the six‐month follow‐up period. Based upon the decision to deliver the final module as a booster, the main outcome measure was modified, prior to study implementation, to be unprotected sex in the past three months at six‐months' post‐intervention.” Comment: outcome was modified but was adjusted appropriately with the changes to the methods.
Other bias	Low risk	Comment: insufficient information to permit judgement

Zhu et al. (2009)

**Table:** 

**Methods**	Cluster randomised‐controlled trial in China Study date not reported.
**Participants**	Eight pairs of hospitals available for data analysis. Total of 2336 women younger than 25 years (555 before and 555 after the simple intervention package; 634 before and 592 after the comprehensive intervention package).
**Interventions**	Intervention: Two post‐abortion family planning (FP) service packages. One FP service package included provision of limited information and referral to existing FP services. The other (2) was a more comprehensive package which consisted—in addition to the above simple package—of individual counselling, free provision of contraceptive materials, and involvement of the male partner. Control: comparison between two interventions Setting: facility level Timing of intervention: Moderators delivering: gynaecologists
**Outcomes**	Primary: contraceptive use, repeat abortion rate, pregnancies Secondary
**Notes**	Women were pregnant when enrolled. ICC value of 0.35, from Jewkes et al., 2006 used as they have the same setting (community). Funding: EU 6th Framework Programme Declaration of interest: reported no conflicts of interest

Risk of bias table

**Table:** 

Bias	Authors' judgement	Support for judgement
Random sequence generation (selection bias)	Low risk	Quote: “Randomization was done by coin tossing by a neutral person who was not involved in the study at one research centre.” Comment: adequately done
Allocation concealment (selection bias)	Low risk	Comment: since it was a cluster randomised trial, allocation concealment should not be an issue as in this design all the clusters are randomised.
Similar baseline characteristics	Unclear risk	
Similar baseline outcome measurement	Unclear risk	
Blinding of participants and personnel (performance bias)	High risk	Quote: The interviews were not blinded to interviewers regarding intervention package that the women had received.” Comment: not adequately done
Blinding of outcome assessment (detection bias)	Unclear risk	Comment: insufficient information to permit judgement
Incomplete outcome data (attrition bias)	Low risk	Quote: “they represented 59.0% of those who had been interviewed at the time of the abortion with no substantial differences in follow‐up rate between intervention packages and between before and after interventions.” Comment: adequately done
Prevention of knowledge of allocated intervention	Unclear risk	
Protection against contamination	Unclear risk	
Selective reporting (reporting bias)	Unclear risk	Comment: insufficient information to permit judgement and no protocol was found
Other bias	Unclear risk	Quote: “Before intervention, the rates of pregnancies, unwanted pregnancies, and induced abortions during the six‐month follow‐up period among women seeking abortion at hospitals allocated to Package B were already lower than those of hospitals allocated to Package A. We believe this must be due to chance since these data were collected before the randomisation. Shanghai had already had some integration of FP within abortion services at hospitals, while Beijing and Zhengzhou had little or no integrated FP at hospitals when the trial started.” Quote: possible baseline outcome differences

### Characteristics of excluded studies

**Table:** 

Aarons et al. (2000)	
**Reason for exclusion**	This trial took place in USA which is a high income country and so, out of the scope of this review.
Agha et al. (2004)	
**Reason for exclusion**	The study was conducted in high‐income country
Aguayo et al. (2013)	
**Reason for exclusion**	Study design is not appropriate (programme evaluation and summary of previous studies).
Ahmed et al. (2005)	
**Reason for exclusion**	This study had the same dosage of iron‐folic acid in all arms.
Ahmed et al. (2010)	
**Reason for exclusion**	This study had the same dosage of iron‐folic acid in all arms.
**Allen (** 1997 **)**	
**Reason for exclusion**	This trial took place in USA which is a high income country and so, out of the scope of this review.
Anderson et al. (1999)	
**Reason for exclusion**	This trial took place in USA which is a high income country and so, out of the scope of this review.
Angeles‐Adgeppa et al. (2005)	
**Reason for exclusion**	The intervention was of iron and folic acid with same concentration of folic acid in all groups.
Angeles‐Agdeppa et al. (1997)	
**Reason for exclusion**	The intervention arm provided Vitaminc C and retinol
**Antunes (** 2002 **)**	
**Reason for exclusion**	This trial was not relevant to delaying pregnancy.
Badger (1981)	
**Reason for exclusion**	This trial took place in USA which is a high income country and so, out of the scope of this review.
Bandiera et al. (2012)	
**Reason for exclusion**	This trial was not concerned with prolonging interpregnancy intervals
**Baptiste (2005)**	
**Reason for exclusion**	This trial had no intervention to delay pregnancy.
Barnet et al. (2010)	
**Reason for exclusion**	This trial took place in UK which is a high income country and so, out of the scope of this review.
Basen‐Engquist (2001)	
**Reason for exclusion**	This trial took place in USA which is a high income country and so, out of the scope of this review.
Beasley (2000)	
**Reason for exclusion**	This study only has iron supplementation without folic acid supplementation.
Black et al. (2006)	
**Reason for exclusion**	This trial took place in USA which is a high income country and so, out of the scope of this review.
Blake et al. (2001)	
**Reason for exclusion**	This trial took place in USA which is a high income country and so, out of the scope of this review.
Boekeloo et al. (1999)	
**Reason for exclusion**	This trial took place in USA which is a high income country and so, out of the scope of this review.
Bonell et al. (2005)	
**Reason for exclusion**	This trial took place in UK which is a high income country and so, out of the scope of this review.
Bonell et al. (2013)	
**Reason for exclusion**	This trial took place in UK which is a high income country and so, out of the scope of this review.
Borgia et al. (2005)	
**Reason for exclusion**	This trial took place in Italy which is a high income country and so, out of the scope of this review.
Brieger (2001)	
**Reason for exclusion**	This was a ecologic study
Buston et al. (2007)	
**Reason for exclusion**	This trial took place in UK which is a high income country and so, out of the scope of this review.
**Calvo (** 2008 **)**	
**Reason for exclusion**	Flour fortification was done which is not included in this review.
Cave et al. (1993)	
**Reason for exclusion**	This trial took place in USA which is a high income country and so, out of the scope of this review.
**Chen (** 2008 **)**	
**Reason for exclusion**	The study was conducted in high‐income country
Chen (2010)	
**Reason for exclusion**	The trial compared folic acid and other micronutrients to a control group.
Chesney et al. (2003)	
**Reason for exclusion**	This trial took place in USA which is a high income country and so, out of the scope of this review.
**Chung‐Park (2003)**	
**Reason for exclusion**	This trial took place in USA which is a high income country and so, out of the scope of this review.
Clark et al. (2005)	
**Reason for exclusion**	This trial took place in USA which is a high income country and so, out of the scope of this review.
Cowan et al. (2008)	
**Reason for exclusion**	This trial was excluded as it only presented baseline data, the impact of the program was not assessed in this paper.
Coyle et al. (1999)	
**Reason for exclusion**	This trial took place in USA which is a high income country and so, out of the scope of this review.
Coyle et al. (2004)	
**Reason for exclusion**	This trial took place in USA which is a high income country and so, out of the scope of this review.
Coyle et al. (2006)	
**Reason for exclusion**	This trial took place in USA which is a high income country and so, out of the scope of this review.
Crape et al. (2005)	
**Reason for exclusion**	This study has no control group.
Czeizel and Dobo (1994)	
**Reason for exclusion**	This study took place in Hungary which is a high income country and so, out of the scope of this review.
Danielson et al. (1990)	
**Reason for exclusion**	This trial took place in USA which is a high income country and so, out of the scope of this review.
**Decat (** 2015 **)**	
**Reason for exclusion**	This study was a cross sectional survey and there was no intervention taking place.
Deshmukh (2008)	
**Reason for exclusion**	This study has no control group.
DiClemente et al. (2004)	
**Reason for exclusion**	This trial took place in USA which is a high income country and so, out of the scope of this review.
**Dilorio (2006)**	
**Reason for exclusion**	This trial took place in USA which is a high income country and so, out of the scope of this review.
**Dilorio (2007)**	
**Reason for exclusion**	This trial took place in USA which is a high income country and so, out of the scope of this review.
Dongre et al. (2011)	
**Reason for exclusion**	This study has no control group.
Doubova et al. (2017)	
**Reason for exclusion**	The study was conducted in high‐income country
Downs et al. (2004)	
**Reason for exclusion**	This trial took place in USA which is a high income country and so, out of the scope of this review.
Drayton 2000	
**Reason for exclusion**	Abstract only
Eisen (1987)	
**Reason for exclusion**	This trial took place in USA which is a high income country and so, out of the scope of this review.
Eisen et al. (1990)	
**Reason for exclusion**	This trial took place in USA which is a high income country and so, out of the scope of this review.
**El Bassel (** 2003 **)**	
**Reason for exclusion**	This trial took place in USA which is a high income country and so, out of the scope of this review.
**Elliot (2012)**	
**Reason for exclusion**	This trial took place in UK which is a high income country and so, out of the scope of this review.
Elster et al. (1987)	
**Reason for exclusion**	This trial took place in USA which is a high income country and so, out of the scope of this review.
Fawole et al. (1999)	
**Reason for exclusion**	The study was conducted in high‐income country
Ferguson (1998)	
**Reason for exclusion**	This trial took place in USA which is a high income country and so, out of the scope of this review.
Field et al. (1982)	
**Reason for exclusion**	This trial took place in USA which is a high income country and so, out of the scope of this review.
García et al. (2012)	
**Reason for exclusion**	This trial included female sex workers as participants.
**Gaughran (2013)**	
**Reason for exclusion**	This trial was a KAP (knowledge, attitudes and practices) study.
Gonzalez‐Rosendo (2002)	
**Reason for exclusion**	This study only has iron supplementation without folic acid supplementation.
Graham et al. (2002)	
**Reason for exclusion**	This trial took place in UK which is a high income country and so, out of the scope of this review.
Guilamo‐Ramos et al. (2011)	
**Reason for exclusion**	This trial took place in USA which is a high income country and so, out of the scope of this review.
Gunaratna et al. (2015)	
**Reason for exclusion**	All arms in this study received the same dose of folic acid.
Hahn et al. (1994)	
**Reason for exclusion**	This trial took place in USA which is a high income country and so, out of the scope of this review.
Harper et al. (2009)	
**Reason for exclusion**	This trial took place in USA which is a high income country and so, out of the scope of this review.
**Henderson (2006)**	
**Reason for exclusion**	This trial took place in Scotland which is a high income country and so, out of the scope of this review.
Herceg‐Baron et al. (1986)	
**Reason for exclusion**	This trial took place in USA which is a high income country and so, out of the scope of this review.
**Herceg‐Brown (1989)**	
**Reason for exclusion**	This trial took place in USA which is a high income country and so, out of the scope of this review.
Horjus et al. (2005)	
**Reason for exclusion**	Both comparison groups recieved supplementation, one for 5 months and another for 8 months.
Howard and McCabe (1990)	
**Reason for exclusion**	This trial took place in USA which is a high income country and so, out of the scope of this review.
Hubacher (2012)	
**Reason for exclusion**	Compared sub dermal implants with short acting harmonal method
Hulton (2007)	
**Reason for exclusion**	This trial took place in USA which is a high income country and so, out of the scope of this review.
ICMR (2000)	
**Reason for exclusion**	The study given folic acid with used other micronutrients as well with folic acid in the intervention arm
Jay et al. (1984)	
**Reason for exclusion**	This trial took place in USA which is a high income country and so, out of the scope of this review.
Jayatissa (1999)	
**Reason for exclusion**	Both Daily and weekly intervention groups recieved Vitamin C and compared with placebo.
**Jemmot (** 1998 **)**	
**Reason for exclusion**	This trial took place in USA which is a high income country and so, out of the scope of this review.
**Jemmot (** 2005 **)**	
**Reason for exclusion**	This trial took place in USA which is a high income country and so, out of the scope of this review.
**Jemmot (** 2010 **)**	
**Reason for exclusion**	This trial took place in USA which is a high income country and so, out of the scope of this review.
**Jemmott III (** 2010 **)**	
**Reason for exclusion**	The study was conducted in high‐income country
Jennings (2014)	
**Reason for exclusion**	This trial took place in USA which is a high income country and so, out of the scope of this review.
Jennings (2016)	
**Reason for exclusion**	The study did not disaggregated data based on sex.
**Kamali (** 2002 **)**	
**Reason for exclusion**	This trial did not have any intervention to delay pregnancy.
Kan (2012)	
**Reason for exclusion**	This trial took place in USA which is a high income country and so, out of the scope of this review.
Katz et al. (2011)	
**Reason for exclusion**	This trial took place in USA which is a high income country and so, out of the scope of this review.
Kelsey et al. (2001)	
**Reason for exclusion**	This trial took place in USA which is a high income country and so, out of the scope of this review.
Key et al. (2001)	
**Reason for exclusion**	This trial took place in USA which is a high income country and so, out of the scope of this review.
**Kianfar (** 2000 **)**	
**Reason for exclusion**	This study only has iron supplementation without folic acid supplementation.
Kiene and Barta (2006)	
**Reason for exclusion**	This trial took place in USA which is a high income country and so, out of the scope of this review.
**Kim (** 2001 **)**	
**Reason for exclusion**	The study was conducted in high‐income country
Kinsler et al. (2004)	
**Reason for exclusion**	The study was conducted in high‐income country
Kirby et al. (1997)	
**Reason for exclusion**	This trial took place in USA which is a high income country and so, out of the scope of this review.
**Kirby (** 2004 **)**	
**Reason for exclusion**	This trial took place in USA which is a high income country and so, out of the scope of this review.
Kirke (1992)	
**Reason for exclusion**	This trial took place in Ireland which is a high income country and so, out of the scope of this review.
Kogan et al. (2012)	
**Reason for exclusion**	This trial took place in USA which is a high income country and so, out of the scope of this review.
Koniak‐Griffin et al. (2003)	
**Reason for exclusion**	This trial took place in USA which is a high income country and so, out of the scope of this review.
Koo et al. (2011)	
**Reason for exclusion**	This trial took place in USA which is a high income country and so, out of the scope of this review.
**Kotecha (** 2009 **)**	
**Reason for exclusion**	This study compares baseline data to post intervention and has no comparison group.
Kyrychenko (2006)	
**Reason for exclusion**	The study was conducted in high‐income country
Laurence et al. (1981)	
**Reason for exclusion**	This trial took place in UK which is a high income state and so, out of the scope of this review.
Leenstra et al. (2009)	
**Reason for exclusion**	This study only has iron supplementation without folic acid supplementation.
Lopes (1999)	
**Reason for exclusion**	This study only has iron supplementation without folic acid supplementation.
López‐Camelo et al. (2005)	
**Reason for exclusion**	Fortified wheat flour was used which is out of the scope of this review.
Magnani et al. (2005)	
**Reason for exclusion**	This trial did not have a control group.
Manizheh et al. (2009)	
**Reason for exclusion**	The women in this study received the intervention in early pregnancy instead of in the periconceptional period.
Marcell et al. (2013)	
**Reason for exclusion**	This trial had participants consisting of young adult males only.
Markham et al. (2012)	
**Reason for exclusion**	This trial took place in USA which is a high income country and so, out of the scope of this review.
Mathews et al. (2016)	
**Reason for exclusion**	The study was conducted in high‐income country
Mba et al. (2007)	
**Reason for exclusion**	The study was conducted in high‐income country
Meekers et al. (2005)	
**Reason for exclusion**	This trial did not directly assess the impact of the intervention. It compared 2000 and 2002 data from a reproductive health survey.
Minnis et al. (2014)	
**Reason for exclusion**	This trial took place in USA which is a high income country and so, out of the scope of this review.
Mitchell‐DiCenso et al. (1997)	
**Reason for exclusion**	This trial took place in Canada which is a high income country and so, out of the scope of this review.
Moberg and Piper (1998)	
**Reason for exclusion**	This trial took place in USA which is a high income country and so, out of the scope of this review.
Morrison‐Beedy et al. (2013)	
**Reason for exclusion**	This trial took place in USA which is a high income country and so, out of the scope of this review.
Mozaffari‐Khosravi et al. (2010)	
**Reason for exclusion**	This study only has iron supplementation without folic acid supplementation.
**MRC (1991)**	
**Reason for exclusion**	This study took place in UK, Israel, Hungary, Australia and Canada which are a high income countries and so, out of the scope of this review.
Munodawafa et al. (1995)	
**Reason for exclusion**	The study was conducted in high‐income country
Nguyen et al. (2008)	
**Reason for exclusion**	Only proovided folic acid and iron to women with severe anemia.
Nguyen et al. (2012)	
**Reason for exclusion**	This study examines the use of vouchers to avail maternal health services post delivery and hence, is out of the scope of this review.
Norton et al. (2012)	
**Reason for exclusion**	This trial took place in USA which is a high income country and so, out of the scope of this review.
**Ozcebe (** 2004 **)**	
**Reason for exclusion**	This trial did not have a true control group, instead the post‐intervention outcomes were only compared with the baseline data.
**O'Donnell (1998)**	
**Reason for exclusion**	This trial took place in USA which is a high income country and so, out of the scope of this review.
O'Donnell et al. (2002)	
**Reason for exclusion**	This trial took place in USA which is a high income country and so, out of the scope of this review.
O'Sullivan and Jacobsen (1992)	
**Reason for exclusion**	This trial took place in USA which is a high income country and so, out of the scope of this review.
Palermo et al. (2016)	
**Reason for exclusion**	This trial focused on childbearing. The participants had already been pregnant and given birth by the time of the trial.
**Pasricha (2009)**	
**Reason for exclusion**	This study compares baseline data to post intervention and has no comparison group.
Pereira et al. (2017)	
**Reason for exclusion**	This trial was a cross‐sectional study and just assessed data at one point in time without an intervention.
Peskin et al. (2015)	
**Reason for exclusion**	This trial took place in USA which is a high income country and so, out of the scope of this review.
**Philiber (2001)**	
**Reason for exclusion**	This trial took place in USA which is a high income country and so, out of the scope of this review.
Pinkleton et al. (2008)	
**Reason for exclusion**	This trial took place in USA which is a high income country and so, out of the scope of this review.
Polit and Kahn (1985)	
**Reason for exclusion**	This trial took place in USA which is a high income country and so, out of the scope of this review.
**Quinlivan (** 2003 **)**	
**Reason for exclusion**	This trial took place in USA which is a high income country and so, out of the scope of this review.
Quint et al. (1997)	
**Reason for exclusion**	This trial took place in USA which is a high income country and so, out of the scope of this review.
Raine et al. (2005)	
**Reason for exclusion**	This trial took place in USA which is a high income country and so, out of the scope of this review.
Raymond (2006)	
**Reason for exclusion**	This trial took place in USA which is a high income country and so, out of the scope of this review.
**Rocha (2004)**	
**Reason for exclusion**	This trial could not be interpreted clearly given that only a rough English translation of the originally Spanish article could be obtained in which the tables were still in Spanish.
Roschnik et al. (2004)	
**Reason for exclusion**	Results are not segregated by gender in this study.
Roschnik et al. (2008)	
**Reason for exclusion**	Study design is not appropriate (summary of numerous studies).
Rosenberg et al. (2015)	
**Reason for exclusion**	This was a cohort study
Sayed et al. (2008)	
**Reason for exclusion**	This study took place in South Africa which is a high income country and so, out of the scope of this review.
Sebastian et al. (2012)	
**Reason for exclusion**	This trial was related to birth spacing and not delaying pregnancy.
Shrier et al. (2001)	
**Reason for exclusion**	This trial took place in USA which is a high income country and so, out of the scope of this review.
Sieving et al. (2011)	
**Reason for exclusion**	This trial took place in USA which is a high income country and so, out of the scope of this review.
Sims and Luster (2002)	
**Reason for exclusion**	This trial took place in USA which is a high income country and so, out of the scope of this review.
Smith (1994)	
**Reason for exclusion**	This trial took place in USA which is a high income country and so, out of the scope of this review.
Solomon and Liefeld (1998)	
**Reason for exclusion**	This trial took place in USA which is a high income country and so, out of the scope of this review.
Stephenson et al. (2004)	
**Reason for exclusion**	This trial took place in UK which is a high income country and so, out of the scope of this review.
Taylor et al. (2001)	
**Reason for exclusion**	Results are not segregated by gender in this study.
Taylor et al. (2014)	
**Reason for exclusion**	The study is from high income setting
Tee 1999	
**Reason for exclusion**	All arms in this study received the same dose of folic acid.
Trenholm et al. (2007)	
**Reason for exclusion**	This trial took place in USA which is a high income country and so, out of the scope of this review.
Villarruel et al. (2006)	
**Reason for exclusion**	This trial took place in USA which is a high income country and so, out of the scope of this review.
Villarruel et al. (2008)	
**Reason for exclusion**	This trial had parents as the target participants and the outcome was communication.
Vyas et al., (2010)	
**Reason for exclusion**	Compared daily group of supplementation with leafy concentrate supplementation group.
**Wagner (** 1999 **)**	
**Reason for exclusion**	This trial took place in USA which is a high income country and so, out of the scope of this review.
Westphal et al. (2004)	
**Reason for exclusion**	In this study, an unspecified amount of folic acid was given. None of the outcomes reported were relevant to this review.
Wiggins et al. (2005)	
**Reason for exclusion**	This trial took place in the UK, which is a high income country and so, out of the scope of this review.
Wight et al. (2002)	
**Reason for exclusion**	This trial took place in UK which is a high income country and so, out of the scope of this review.
Wu et al. (2003)	
**Reason for exclusion**	This trial took place in USA which is a high income country and so, out of the scope of this review.
Yusoff et al. (2012)	
**Reason for exclusion**	Results are not segregated by gender in this study.
Zavaleta et al. (2000)	
**Reason for exclusion**	This study only has iron supplementation without folic acid supplementation.

## DATA AND ANALYSES

**1 Delay in age of first pregnancy—Education on sexual health and contraception versus no intervention**

**Table:** 

Outcome or subgroup	Studies	Participants	Statistical method	Effect estimate
1.1 Unintended pregnancy	2	490	Risk Ratio (M‐H, Random, 95% CI)	0.42 [0.07, 2.36]
1.2 Knowledge ‐ Pregnancy prevention	5	1433	Risk Ratio (M‐H, Random, 95% CI)	1.02 [0.87, 1.21]
1.3 initiation of sexual activity	4		Risk Ratio (M‐H, Random, 95% CI)	Subtotals only
1.3.1 3 month follow up	1	56	Risk Ratio (M‐H, Random, 95% CI)	0.43 [0.04, 4.51]
1.3.2 6 month follow up	2	1443	Risk Ratio (M‐H, Random, 95% CI)	1.02 [0.57, 1.83]
1.3.3 12 month follow up	1	1387	Risk Ratio (M‐H, Random, 95% CI)	0.70 [0.49, 0.99]
1.3.4 After 3 years	2	1153	Risk Ratio (M‐H, Random, 95% CI)	0.95 [0.79, 1.14]
1.4 Use of any contraception	3	2991	Risk Ratio (M‐H, Random, 95% CI)	2.45 [1.19, 5.06]
1.4.1 current use	1	2080	Risk Ratio (M‐H, Random, 95% CI)	4.69 [3.22, 6.83]
1.4.2 ever use	2	911	Risk Ratio (M‐H, Random, 95% CI)	1.71 [1.42, 2.05]
1.5 Use of a modern method	2	1028	Risk Ratio (M‐H, Random, 95% CI)	2.12 [0.64, 7.07]
1.5.1 current use	1	405	Risk Ratio (M‐H, Random, 95% CI)	1.21 [0.99, 1.49]
1.5.2 ever use	1	623	Risk Ratio (M‐H, Random, 95% CI)	3.89 [2.28, 6.63]
1.6 Use of a traditional method	1	623	Risk Ratio (M‐H, Random, 95% CI)	1.70 [0.94, 3.07]
1.7 Use of condoms	8		Risk Ratio (M‐H, Random, 95% CI)	Subtotals only
1.7.1 current use	5	1175	Risk Ratio (M‐H, Random, 95% CI)	0.93 [0.81, 1.06]
1.7.2 ever use	6	1604	Risk Ratio (M‐H, Random, 95% CI)	1.54 [1.08, 2.20]
1.8 Use of pills	1	288	Risk Ratio (M‐H, Random, 95% CI)	1.34 [0.89, 2.01]
1.9 Use of depot/injectable methods	1	288	Risk Ratio (M‐H, Random, 95% CI)	1.58 [1.26, 1.98]
1.10 Abortions	1	80	Risk Ratio (M‐H, Random, 95% CI)	1.11 [0.07, 17.06]

**2 Delay in age of first pregnancy—Education on sexual health and contraception versus no intervention (Study design)**

**Table:** 

Outcome or subgroup	Studies	Participants	Statistical method	Effect estimate
2.1 Knowledge ‐ Pregnancy prevention	5	1433	Risk Ratio (M‐H, Random, 95% CI)	1.02 [0.87, 1.21]
2.1.1 RCT	2	178	Risk Ratio (M‐H, Random, 95% CI)	1.49 [1.10, 2.03]
2.1.2 Quasi RCT	3	1255	Risk Ratio (M‐H, Random, 95% CI)	0.94 [0.79, 1.12]

**3 Delay in age of first pregnancy—Education on sexual health + provision of contraceptives versus no intervention**

**Table:** 

Outcome or subgroup	Studies	Participants	Statistical method	Effect estimate
3.1 Use of any contraception	1	1908	Risk Ratio (M‐H, Random, 95% CI)	1.49 [0.81, 2.75]
3.1.1 regular use	1	954	Risk Ratio (M‐H, Random, 95% CI)	1.90 [1.71, 2.10]
3.1.2 ever use	1	954	Risk Ratio (M‐H, Random, 95% CI)	1.17 [1.12, 1.22]
3.2 Condom use	1	954	Risk Ratio (M‐H, Random, 95% CI)	1.14 [1.09, 1.19]

**4 Optimising interpregnancy interval—Education on sexual health and contraception versus no intervention**

**Table:** 

Outcome or subgroup	Studies	Participants	Statistical method	Effect estimate
4.1 use of any contraception	2		Risk Ratio (M‐H, Random, 95% CI)	Subtotals only
4.1.1 Education + involvement of male partner and Provision of contraceptive	1	338	Risk Ratio (M‐H, Random, 95% CI)	1.83 [1.26, 2.66]
4.1.2 Education alone	2	2385	Risk Ratio (M‐H, Random, 95% CI)	2.72 [0.88, 8.40]
4.2 use of a modern method	1		Risk Ratio (M‐H, Random, 95% CI)	Subtotals only
4.2.1 Education + involvement of male partner and Provision of contraceptive	1	338	Risk Ratio (M‐H, Random, 95% CI)	2.25 [1.29, 3.93]
4.2.2 Education alone	1	338	Risk Ratio (M‐H, Random, 95% CI)	2.45 [1.42, 4.24]

**5 Optimising interpregnancy interval—Education on sexual health + provision of contraception + involvement of male partner versus education on sexual health alone**

**Table:** 

Outcome or subgroup	Studies	Participants	Statistical method	Effect estimate
5.1 Unintended pregnancies	1	45	Risk Ratio (M‐H, Random, 95% CI)	0.32 [0.01, 7.45]
5.2 use of any contraceptive method	1	39	Risk Ratio (M‐H, Random, 95% CI)	1.05 [0.91, 1.21]
5.3 use of condoms, OCs, IUDs and implants	1	39	Risk Ratio (M‐H, Random, 95% CI)	1.05 [0.88, 1.26]
5.4 induced abortion	1	45	Risk Ratio (M‐H, Random, 95% CI)	0.32 [0.01, 7.45]

**6 Periconceptional folic acid supplementation versus placebo**

**Table:** 

Outcome or subgroup	Studies	Participants	Statistical method	Effect estimate
6.1 Neural tube defects	2	248,056	Risk Ratio (M‐H, Random, 95% CI)	0.53 [0.41, 0.67]
6.1.1 Non RCTs	2	248,056	Risk Ratio (M‐H, Random, 95% CI)	0.53 [0.41, 0.67]
6.2 Neural tube defects	2	248,056	Risk Ratio (M‐H, Random, 95% CI)	0.53 [0.41, 0.67]
6.2.1 400 μg/0.4 mg	1	247,831	Risk Ratio (M‐H, Random, 95% CI)	0.53 [0.42, 0.68]
6.2.2 5 mg	1	225	Risk Ratio (M‐H, Random, 95% CI)	0.14 [0.01, 2.50]

**7 Periconceptional iron folic acid supplementation versus placebo**

**Table:** 

Outcome or subgroup	Studies	Participants	Statistical method	Effect estimate
7.1 Anemia	6	3430	Risk Ratio (M‐H, Random, 95% CI)	0.66 [0.53, 0.81]
7.1.1 RCTs	6	3430	Risk Ratio (M‐H, Random, 95% CI)	0.66 [0.53, 0.81]
7.2 Anemia	6		Risk Ratio (M‐H, Random, 95% CI)	Subtotals only
7.2.1 Weekly supplementation	6	2661	Risk Ratio (M‐H, Random, 95% CI)	0.70 [0.55, 0.88]
7.2.2 Daily supplementation	2	1532	Risk Ratio (M‐H, Random, 95% CI)	0.49 [0.21, 1.12]
7.3 Anemia	6	2661	Risk Ratio (M‐H, Random, 95% CI)	0.70 [0.55, 0.88]
7.3.1 8 weeks of weekly supplementation	1	159	Risk Ratio (M‐H, Random, 95% CI)	1.17 [0.83, 1.67]
7.3.2 10 weeks of weekly supplementation	1	552	Risk Ratio (M‐H, Random, 95% CI)	0.75 [0.64, 0.88]
7.3.3 12 weeks of weekly supplementation	1	145	Risk Ratio (M‐H, Random, 95% CI)	0.39 [0.27, 0.57]
7.3.4 14 weeks of weekly supplementation	1	139	Risk Ratio (M‐H, Random, 95% CI)	0.21 [0.11, 0.39]
7.3.5 16 weeks of weekly supplementation	1	1386	Risk Ratio (M‐H, Random, 95% CI)	0.89 [0.79, 0.99]
7.3.6 24 weeks of weekly supplementation	1	280	Risk Ratio (M‐H, Random, 95% CI)	0.85 [0.77, 0.94]
7.4 Anemia	6	3430	Risk Ratio (M‐H, Random, 95% CI)	0.66 [0.53, 0.81]
7.4.1 School	4	3005	Risk Ratio (M‐H, Random, 95% CI)	0.66 [0.51, 0.86]
7.4.2 Work	2	425	Risk Ratio (M‐H, Random, 95% CI)	0.59 [0.24, 1.43]
7.5 Adverse effects	1	280	Risk Ratio (M‐H, Random, 95% CI)	0.63 [0.38, 1.05]

## SOURCES OF SUPPORT

### INTERNAL SOURCES

No sources of support provided

### EXTERNAL SOURCES

Bill and Melinda Gates Foundation, USA.

## APPENDIX A

### Search strategy

**Pubmed (NIH)‐ Delay/Interval (Search date July 31, 2019)**

**[1]** "Preconception Care"[Mesh] OR (("preconception"[Text Word] OR "preconception care"[Text Word] OR "periconception"[Text Word] OR "pre‐pregnancy"[Text Word])) OR ("preconception"[Title/Abstract] OR "preconception care"[Title/Abstract] OR "periconception"[Title/Abstract] OR "pre‐pregnancy"[Title/Abstract])

**[2]** (("pregnancy interval*"[Text Word] OR "birth spacing"[Text Word] OR "first birth interval"[Text Word] OR "interpregnancy interval"[Text Word] OR "intergenesic interval"[Text Word] OR "pregnancy spacing"[Text Word] OR "family planning"[Text Word] OR "delaying childbearing"[Text Word] OR "delaying pregnancy"[Text Word] OR "delay pregnancy"[Text Word]) OR ("pregnancy interval*"[Title/Abstract] OR "birth spacing"[Title/Abstract] OR "first birth interval"[Title/Abstract] OR "interpregnancy interval"[Title/Abstract] OR "intergenesic interval"[Title/Abstract] OR "pregnancy spacing"[Title/Abstract] OR "family planning"[Title/Abstract] OR "delaying childbearing"[Title/Abstract] OR "delaying pregnancy"[Title/Abstract] OR "delay pregnancy"[Title/Abstract])) OR birth intervals[MeSH Terms]

**[3]** ("reproductive aged women"[Text Word] OR "adolescen*"[Text Word] OR "teen*"[Text Word] OR women[Text Word]) OR (("Adolescent"[Mesh])

**[1] AND [2]**

**[1] AND [2] AND [3]**

**PubMed/Medline (NIH): Folic/iron acid (Search date July 31, 2019)**

**[1]** "Preconception Care"[Mesh] OR (("preconception"[Text Word] OR "preconception care"[Text Word] OR "periconception"[Text Word] OR "pre‐pregnancy"[Text Word])) OR ("preconception"[Title/Abstract] OR "preconception care"[Title/Abstract] OR "periconception"[Title/Abstract] OR "pre‐pregnancy"[Title/Abstract])

**[2]** (((((("iron folic acid"[Text Word]) OR "iron folic acid"[Title/Abstract]) OR "folic acid"[MeSH Major Topic]) OR "folic acid"[Text Word]) OR "folic acid"[Title/Abstract]) OR "iron folic acid supplementation") OR "folic acid supplementation"

**[3]** ((("reproductive aged women" OR "adolescen*" OR "teen*" OR women)) OR ((("Adolescent"[Mesh]) OR "Menstruation"[Mesh]) OR "Puberty"[Mesh]))

**[1] AND [2] AND [3**

**1] AND [2] AND [3] AND NOT “pregnant”**

**Embase ‐ Delay/Interval (Elsevier) (Search date July 31, 2019)**

**[1]** 'prepregnancy care' OR 'preconception' OR 'periconception' OR 'preconception care'/exp

**[2]** 'pregnancy interval*' OR 'birth spacing' OR 'first birth interval' OR 'interpregnancy interval' OR 'intergenesic interval' OR 'pregnancy spacing' OR 'family planning'/exp OR 'delaying childbearing' OR 'delaying pregnancy' OR 'delay pregnancy' OR 'reproductive life plan*'

**OR** with:ab.ti

**[3]** 'reproductive aged women' OR 'teen*' OR 'adoles*' OR 'girl*' OR adolescent/exp

**[1] AND [2] AND [3]**

**[1] AND [2]**

**Embase: Iron/Folic Acid (Elsevier) (Search date July 31, 2019)** ‐

**[1]** 'prepregnancy care' OR 'preconception' OR 'periconception' OR 'preconception care'/exp

**[2]** 'folic acid'/exp OR 'iron folic acid' OR 'folic acid supplementation' OR 'iron folic acid supplementation' OR iron/exp OR folic acid/exp

**[3]** "reproductive aged women" OR "teen*" OR "adolesc*" OR "girl" OR "pubescent girl*" OR "menstruating girl*" OR "menstruating women"

[1] AND [2] AND [3]

**CINAHL: Delay/Interval (Ebsco) (Search date July 31, 2019)**

**[1]** (MM "Prepregnancy Care") OR "preconception" OR "preconception care" OR "periconception"

**[2]** (MM "Birth Intervals") OR TX "birth intervals" OR TX "birth spacing" OR TX “pregnancy interval” OR TX "interpregnancy interval" OR TX "pregnancy spacing" OR TX "delaying pregnancy" OR TX "delaying childbearing" OR TX "delay pregnancy" OR TX "reproductive life plan" = 484

**[3]** "reproductive aged women" OR (MH "Maternal Age 35 and Over") OR (MH "Adolescent Mothers") OR (MH "Expectant Mothers") OR (MH "Multiparas") OR (MH "Primiparas") OR (MH "Pregnancy in Adolescence") OR "teen* OR (MH "Adolescence") OR TX "girl" OR TX "expectant mother*" OR TX "adolescent mother*"

**[1] AND [2] AND [3]**

**[1] AND [2]**

**CINAHL: Iron/Folic Acid (Ebsco) (Search date July 31, 2019)**

**[1]** (MM "Prepregnancy Care") OR TX "preconception" OR TX "preconception care" OR TX "periconception"

**[2]** (MH "Folic Acid") OR TX "iron folic acid" OR TX "folic acid supplementation" OR TX "folic acid supplement" OR

**[3]** "reproductive aged women" OR "teenager" OR (MH "Adolescence") OR "girl" (MH "Puberty") OR (MH "Menarche") OR TX "pubescent" OR (MH "Puberty") OR (MH "Menarche") OR TX "pubescent girl*" OR TX "menstruating girl*" OR TX "menstruating women"

[1] AND [2] AND [3]

**PsycINFO: Delay/Interval (Ebsco) (Search date July 31, 2019)**

**[1]** ("preconception care" or "prepregnancy care" or "preconception" or "periconception").af.

**[2]** exp family planning/or birth control/or condoms/or delayed parenthood/OR ("birth interval*" or "birth spacing" or "pregnancy interval" or "inter‐pregnancy interval" or "pregnancy spacing" or "delaying pregnancy" or "delaying childbearing" or "delay pregnancy" or "delaying pregnancies" or "delay pregnancies" or "reproductive life plan").af.** = 10,500**

**[3]** adolescent pregnancy/or adolescent mothers/OR

("reproductive aged women" or "teenage girl*" or "adolescent girl*" or "women" or "girl*").af.

**[1] AND [2] AND [3]**

**[1] AND [2] AND [3]**

**PsycINFO: iron/folic acid (Ebsco) (Search date July 31, 2019)**

**[1]** (or "prepregnancy care" or "preconception" or "periconception").af.

**[2]** folic acid/OR "iron folic acid" OR "folic acid supplementation" OR "folic acid supplement" OR "iron folic acid supplement"

**[3]** ("reproductive aged women" or "teenage girl*" or "adolescent girl*" or "women" or "girl*" or "pubescent girl*" or "menstruating girl*" or "menstruating women" or "menarche").af.

**[1] AND [2] AND [3**

**1] AND [2] AND [3] AND COUNTRY FILTERS**

**ERIC: Delay/Interval (ProQuest) (Search date July 31, 2019)**

**[1]** "preconception" OR "periconception" OR "pre‐pregnancy" OR "preconception care" OR "prepregnancy care" (All text words)

**[2]** "family planning" OR "reproductive plan*" OR "reproductive life plan*" OR "contraception" OR "birth control" OR "condom*" OR "birth interval" OR "birth spacing" OR "pregnancy interval" OR "intergenesic interval" OR "pregnancy spacing" OR "inter pregnancy interval" OR "sexual abstinence" OR "sterilisation" OR "delaying pregnanc*" OR "delay pregnanc*" OR "delay childbearing" We only used the ones mentioned.

**[3]** "reproductive aged women" OR "adolescen*" OR "teen*" OR "teenage girl*" OR "girl*" OR "women"

**[1] AND [2] AND [3**

**1] AND [2]**

**ERIC: Iron/Folic acid (ProQuest) (Search date July 31, 2019)**

**[1]** "preconception" OR "periconception" OR "pre‐pregnancy" OR "preconception care" OR "prepregnancy care" (all text words)

**[2]** "iron folic acid" OR "folic acid" OR "folate" OR "iron folic acid supplement*" OR "folic acid supplement*"

**[3]** "reproductive aged women" OR "adolescen*" OR "teen*" OR "teenage girl*" OR "girl*" OR "women"

**[1] AND [2**

**1] AND [2] AND [3]**

**Clinical Trials gov—Delay/Interval**

"Family planning" OR "birth spacing" OR "pregnancy interval" AND "preconception" AND "adolescent"

**Clinical trials gov—Iron/Folic Acid**

"folic acid" OR "iron folic acid" AND "preconception" AND "adolescent"

**CABI Global Health: Delay/Interval (ProQuest) (Search date July 31, 2019) (all text words)**

**[1]** "preconception" OR "periconception" OR "pre‐pregnancy" OR "preconception care" OR "prepregnancy care"

**[2]** "family planning" OR "contraception" OR "birth control" OR "condom*" OR "birth interval" OR "birth spacing" OR "pregnancy interval" OR "pregnancy spacing" OR "inter pregnancy interval" OR "delaying pregnanc*" OR "delay pregnanc*" OR "delay childbearing"

**[3]** "reproductive aged women" OR "adolescen*" OR "teen*" OR "teenage*" OR "girl*" OR "women"

**[1] AND [2] AND [3**

**1] AND [2]**

**CABI Global Health: Iron/Folic acid (ProQuest) (Search date July 31, 2019) (all text words)**

**[1]** "preconception" OR "periconception" OR "pre‐pregnancy" OR "preconception care" OR "prepregnancy care"

**[2]** "iron folic acid" OR "folic acid" OR "folate" OR "iron folic acid supplement*" OR "folic acid supplement*"

**[3]** "reproductive aged women" OR "adolescen*" OR "teen*" OR "girl*" OR "women"

**[1] AND [2**

**1] AND [2] AND [3]**

**Epistemonikos: Delay/Interval ‐ Search date July 31, 2019** (all text words)

**[1]** "Preconception Care" OR "periconception" OR "pre‐pregnancy" OR "preconception"

**[2]** "pregnancy interval*" OR "birth spacing" OR "first birth interval" OR "interpregnancy interval" OR "pregnancy spacing" OR "family planning" OR "delaying childbearing" OR "delaying pregnancy" OR "delay pregnancy"

**[3]** "reproductive aged women" OR "adolescen*" OR "teen*" OR women

**[1] AND [2]**

[1] AND [2] AND [3]

**Epistemonikos: Iron/Folic acid‐ Search date July 31, 2019** (all text words)

**[1]** "Preconception Care" OR "periconception" OR "pre‐pregnancy" OR "preconception"

**[2]** "iron folic acid" OR "iron folic acid" OR "folic acid"

**[3]** "reproductive aged women" OR "adolescen*" OR "teen*" OR women

[1] AND [2] AND [3]

**Scopus (Elsevier): Delay/Interval ‐ Search date July 31, 2019** (all text words)

**[1]** "Preconception Care" OR "periconception" OR "pre‐pregnancy" OR "preconception"

**[2]** "pregnancy interval*" OR "birth spacing" OR "first birth interval" OR "interpregnancy interval" OR "pregnancy spacing" OR "family planning" OR "delaying childbearing" OR "delaying pregnancy" OR "delay pregnancy"

**[3]** "reproductive aged women" OR "adolescen*" OR "teen*" OR women

**[1] AND [2]**

[1] AND [2] AND [3]

**Scopus (Elsevier): Iron/Folic acid ‐ Search date July 31, 2019** (all text words)

**[1]** "Preconception Care" OR "periconception" OR "pre‐pregnancy" OR "preconception"

**[2]** "iron folic acid" OR "iron folic acid" OR "folic acid"

**[3]** "reproductive aged wom*" OR "adolescen*" OR "teen*" OR wom*

[1] AND [2] AND [3]

Differed search terms were used for HMIC, Popline (the database retired in 2019),Dissertation Abstracts International, WHO's Global Health Library, WHO Reproductive Health Library, and the WHO nutrition databases, Journal of Librarianship and Information Science, International Food Policy Research Institute, National Bureau of Economic Research, United States Agency for International Development, World Bank, and Google Scholar. The last search was performed on July 31, 2019, and there was no restriction placed on publication date.
